# Tuning the stability of alkoxyisopropyl protection groups

**DOI:** 10.3762/bjoc.15.70

**Published:** 2019-03-21

**Authors:** Zehong Liang, Henna Koivikko, Mikko Oivanen, Petri Heinonen

**Affiliations:** 1 Department of Chemistry, University of Helsinki, P.O.Box 55 (A.I.Virtasen aukio 1), FIN-00014 Helsinki, Finland

**Keywords:** acetal, hydrolysis, protecting groups

## Abstract

Five different 2-alkoxypropan-2-yl groups are introduced as acid-labile protecting groups for the 5’- and 3’-hydroxy groups of a 2’-deoxynucleoside. All studied protecting groups were readily introduced with good to excellent yields using the appropriate enol ether as a reagent and 0.5 to 1 mol % *p-*toluenesulfonic acid as a catalyst. The protected compounds could be purified by silica gel column chromatography without degradation. The compatibility of these protecting groups in parallel use with benzoyl and silyl groups was verified. The stabilities of the different alkoxy acetal protecting groups were compared by following the kinetics of their hydrolysis at 25.0 °C in buffered solutions through an HPLC method. In the pH range 4.94 to 6.82 the hydrolysis reactions are of first order in the hydronium ion. The rate of hydrolysis correlates with the electron-donating or electron-withdrawing ability of the corresponding alkoxy group. The studied 2-alkoxypropan-2-yl groups and the relative rate constants for their cleavage from the 5’-hydroxy group of 2’-deoxythymidine were: cyclohexyloxy (*k*_rel_ = 7.7), isopropoxy (7.4), methoxy (1), benzyloxy (0.6) and 2,2,2-trifluoroethyloxy (0.04). The attachment of the same groups to the 3’-hydroxy group are from 1.3 to 1.9-fold more stable. The most reactive of these acetone-based acetal groups are faster removed than a dimethoxytrityl group, and they are easier to cleave completely in solution. The structural variation allows steering of the stability and lipophilicity of the compounds in some range.

## Introduction

Acetals form one of the most common types of protecting groups for hydroxy functions. They are easily introduced by a rapid acid-catalyzed reaction and they are also readily cleaved under mild acidic hydrolytic conditions. These properties make the acetal structures usable even in other applications, where easily cleavable linkers are needed [1]. The stability and properties of acetals/ketals widely varies depending on their structure, which widens the scope of their applicability. The acid lability is increased markedly by attaching electron-donating alkyl groups on the acetal carbon. The simplest of the formaldehyde-type acetals, methoxymethyl (MOM) [2], is rather stable to acidic hydrolysis. The acetaldehyde acetals, 1-ethoxyethyl (EE) [2–3] and tetrahydropyranyl (THP) [2] are widely used as protecting groups, but they have a disadvantage of generating an additional asymmetric center. Some structural modifications have been introduced to overcome the chirality problem, e.g., the methoxytetrahydropyranyl group suggested in 1970’s for a substitute of THP in nucleoside chemistry [4–5]. For protection of highly sensitive compounds acetone-based acetals can be applied. However, of these only the 2-methoxypropan-2-yl protecting group (MIP) has been adopted in use [2] and the alternative, 2-benzyloxypropan-2-yl, introduced by Mukaiyama in the 1980’s [6] has not gained popularity.

The MIP group has been used, e.g., in solution-phase oligonucleotide synthesis for the primary 5’-hydroxy group of a nucleoside [7]. The acid lability of MIP is comparable to that of the dimethoxytrityl group (DMTr) that is frequently used to protect this position. The DMTr group works more or less perfectly in solid-phase synthesis, however, in solution-phase processes there are several cases where cleaving of DMTr fails. This is due to the equilibrium formed between the protected and deprotected compounds. During our search for a highly acid-labile protection for the 5’-hydroxy group of a nucleoside we noticed that neither the DMTr nor the MIP are fully satisfactory. Whereas MIP is easily removable, has good atom economy and has the notable advantage of giving volatile hydrolysis products, the small size of this group in some cases leaves the protected compound unnecessarily hydrophilic. This causes solubility problems and complicates isolation and purification steps in the synthetic procedures [7].

We realized that the lability of the acetal group can be easily steered by varying the alkoxy group, while preserving other beneficial properties of the protecting groups. On the other hand, structural modifications can also affect the lipophilicity of the protected compounds. The present study aimed at tuning the properties of acetone-based acetal protecting groups at nucleoside hydroxy functions. We have carried out a systematic kinetic study on the hydrolytic lability of five different 2-alkoxypropan-2-yl groups installed in the 5’- and 3’-hydroxy groups of a 2’-deoxynucleoside. We are well aware of the fact that hydrolysis of acetals has been widely studied over decades and the mechanisms are commonly agreed. However, it appeared that more comprehensive and systematic data are needed to allow the analysis of structural effects in detail.

## Results and Discussion

**Figure 1:**
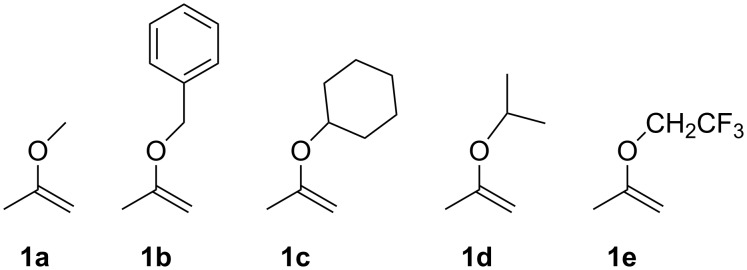
Reagents for acetal protections.

Five acetone-based acetal protecting groups for nucleoside hydroxy groups were introduced by an acid-catalyzed acetalization into compounds **1a–e** (Figure 1). Three of those (**1c–e**) have not been earlier used for acetal protections, although **1c** and **1d** have been synthesized previously [8]. The acetal protecting groups were inserted either to the primary 5’-hydroxy or the secondary 3’-hydroxy group in 2’-deoxythymidine. The labilities of the protecting groups were compared by determining the first order rate constants for the hydrolysis of the protected compounds in buffered solutions between pH 4 to 6.

### Protecting reagents and protection

Two general routes have been reported for the preparation of **1b–e** [6,8]. The 2-benzyloxypropene (**1b**) was synthesized starting from methyl lactate, which was deprotonated and then alkylated with benzyl bromide. Hydrolysis, reduction and elimination yielded **1b** [6]. A shorter route starts from chlorocrotonic acid, in which the substitution of the chloride with the appropriate alkoxide is followed by a thermal decarboxylation step to afford the desired propene derivative [8]. We followed this route to prepare **1c–e** and details of the syntheses are given in Supporting Information File 1.

**Scheme 1:**
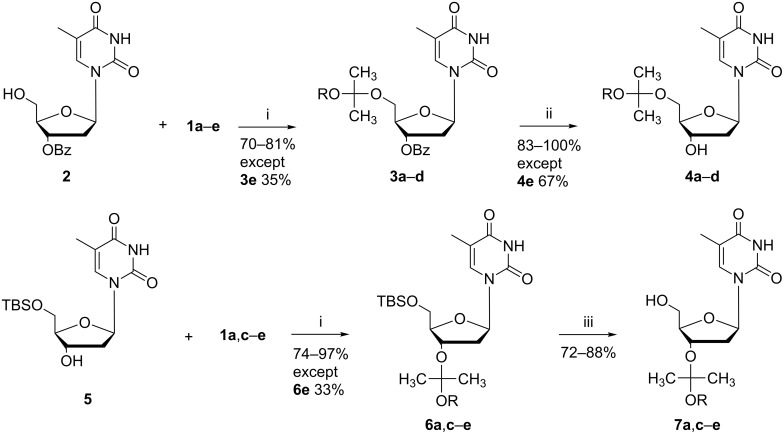
Synthesis of 2-alkoxyprop-2-yl-protected thymidines. Reagents and conditions (i) 7 equiv **1a–e**, 0.5 mol % TsOH in dry THF, 5 min (**1a**–**d**), 18 h (**1e**); (ii) 1:1 conc. aq NH_3_/MeOH, 18 h; (iii) 2 equiv TBAF in dry THF, 1.5 h. Notation: a: R = Me, b: R = Bn, c: R = cyclohexyl, d: R = isopropyl, e: R = CH_2_CF_3_.

The 5’- and 3’-acetal-protected 2’-deoxythymidines were prepared using 7 equiv of the appropriate reagent **1a–e** with the suitably protected thymidine **2** or **5** (Scheme 1) in the presence of *p*-toluenesulfonic acid as the acid catalyst. A suitable amount of the acid catalyst is 0.5 to 1 mol %, with which the reaction takes place practically instantaneous at room temperature with reagents **1a–d**. In order to avoid formation of the vinyl ether analog as a side product, the reaction time should not exceed 5 minutes. The isolated yields of the protected nucleosides are typically between 70–90%. However, reagent **1e** forms an exception, as the electron-withdrawing trifluoroethyl group reduces the reactivity and the reaction time had to be increased to several hours. In addition, the yields of the protected products **3e** and **6e** also remained rather low, around 35%.

**Figure 2:**
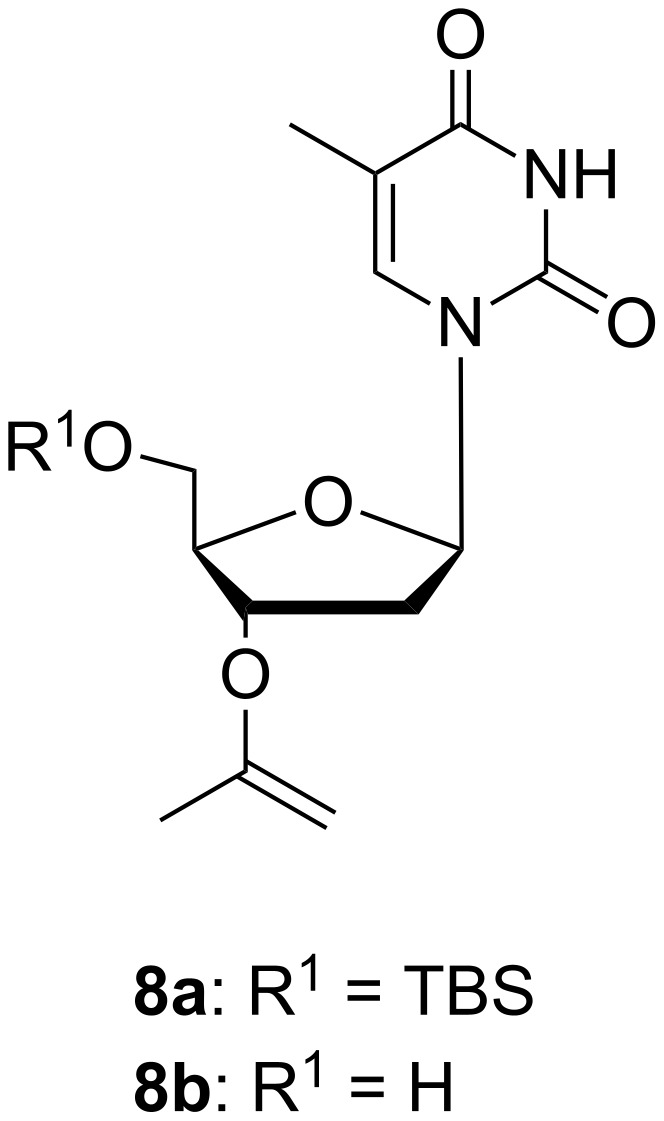
Enol ether **8a:** R^1^ = TBS and **8b:** R^1^ = H.

If a larger amount than 1 mol % of the acid catalyst is used and the reaction time extended, an enol ether formation takes place during the acetalization. We followed the acetal formations by ^1^H NMR spectroscopy, and it was shown that the formed acetal product starts to degrade in a slower consecutive step, which leads to release of the enol ether derivative, e.g., **8a** (Figure 2). Recently, the same kind of enol ether formation was considered in connection to an optimization of the conditions for MIP protection on secondary and benzylic hydroxy groups of mandelonitrile derivatives in a flow reactor process [9].

All of the studied acetals are stable toward basic conditions used to remove benzoyl protecting groups and toward tetrabutylammonium fluoride used to remove the *tert*-butyldimethylsilyl (TBS) protection. The products could be purified by silica gel column chromatography without degradation when 0.5% triethylamine was added to the eluent.

### Hydrolytic stability

The first-order rate constants for the hydrolysis of the acetal protecting groups in the pH range 4.94 to 6.82 at 25.0 °C were determined by an HPLC method and the results are collected in Table 1 as half-life data. Details of kinetic analyses are given in Supporting Information File 1, including a figure of pH–rate profiles. A strictly first-order dependence of the rate on hydronium ion concentration is observed for each acetal derivative in this pH region. The methoxy and benzyloxy acetals show rather similar stabilities, whereas the isopropyloxy and cyclohexyloxy derivatives are on average 7.5 and 7.7-fold faster cleaved than the MIP derivative. The more electron-withdrawing trifluoroethoxy group strongly stabilizes the acetal, making it 30-fold more stable compared to MIP protection.

**Scheme 2:**
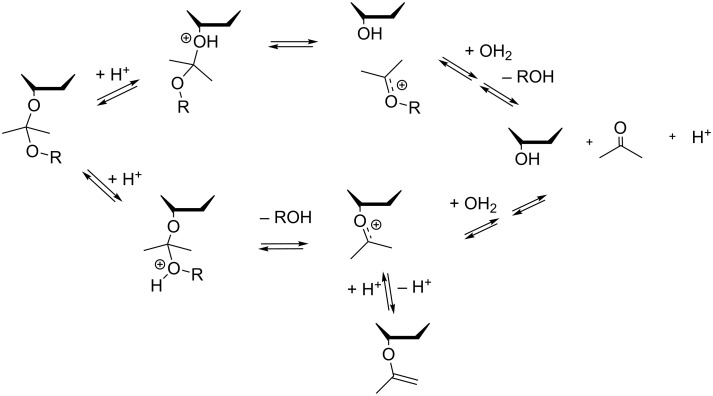
Proposed acetal hydrolysis pathways.

Only minor differences are observed between the stabilities of acetal protection on the primary 5’- and on the secondary 3’-hydroxy functions. Steric effects on the hydrolysis rate are supposed not to be important, because the rate-controlling step of the hydrolysis is suggested to be the unimolecular degradation of the protonated substrate. This releases the stabilized oxocarbenium ion as an intermediate (Scheme 2). Experimental data on the acidity of the 5’- and 3’-hydroxy groups of thymidine are not available, but computed NBO charges suggest that the electronic properties of both hydroxy functions are close to each other [10]. Our DFT level calculations indicated that the 3’-hydroxy function is slightly more acidic than the 5’-hydroxy group, but absolute values for the acidity constants could not be reliably determined.

The hydrolysis of the acetals is suggested to follow most often the A-1 mechanism via formation of an oxocarbenium ion [11]. Polarity effects and the stability of the formed oxocarbenium ion determine, which one of the alkoxy groups is protonated and released as an alcohol. The two proposed competing pathways are illustrated in Scheme 2. Salomaa has shown earlier [12] with formaldehyde acetals that the structural effects on the stability of the oxocarbenium intermediate are directly reflected by the observed hydrolysis rate, whereas the changes in polarity of the leaving alcohol do not give marked changes on the rate. The latter is due to the fact that the kinetic effects on the protonation step and the leaving group ability largely cancel each other.

In our study, the observed rate constants are proportional to the acidity of the alcohol moieties corresponding to the varied stems of the acetal structures: the hydrolysis rate steadily decreases with increasing electron-withdrawing ability of the alkyl group. A comparison of this observation with the results of Salomaa [12] can be taken as support for the upper pathway outlined in Scheme 2, involving protonation of the nucleoside oxygen and departure of it as alcohol. However, it must be noted that comparative structural effects were not studied over a very wide range, and the correlation order may change with using more electron-withdrawing groups than trifluoroethanol. Nevertheless, our interest was in studying the protecting group abilities, and the studied compounds fit the most relevant reactivity area in that sense.

As mentioned above, enol ether derivatives (as **8a**) were formed under more acidic conditions of acetal synthesis. We also prepared the corresponding derivative **8b** of thymidine and studied its hydrolytic stability separately. This is of some interest for discussion of mechanisms of acetal hydrolysis, since the vinyl ether intermediate **8b** could probably be formed on the pathway shown in the lower part of Scheme 2. However, accumulation of **8b** was not observed during our kinetic studies. Compound **8b** was found to be about 15-fold more stable than the cyclohexyl derivative **7c** at pH 4.94, which means that it should have been accumulating as an intermediate, if formed in a significant amount during hydrolysis of **7c** under these conditions. While going towards neutral conditions, the rate of the vinyl ether hydrolysis does not show a linear dependence on the acidity of the solution (see Suppporting Information File 1). Thus, at pH 6.8 the rate of hydrolysis of **8b** is close to that of **7c** and **7d**. The vinyl ether hydrolysis has earlier shown to be even a subject of general acid catalysis [13–14].

It is useful to compare the stabilities of the acetal protections to those of the 4,4’-dimethoxytrityl protecting group used in oligonucleotide synthesis. Directly comparable data are hard to find, since hydrolysis in water solution is not often a very practical method for detritylation, due to hydrophobicity of the trityl derivative. Nevertheless, half-times for the deprotection of 5’-*O*-dimethoxytrityl oligonucleotides have been determined under several different conditions [15]. The values reported were 10 minutes in an acetate buffer at pH 4.18 and approximately 4 minutes at pH 3.22. These values are close to an order of magnitude longer than what we determined for MIP **4a**, and two orders of magnitude longer than those of the cyclohexyl and isopropyl analogs **4c**, **4d** (Table 1). It must be noted, however, that the removal of the DMTr protection is very dependent on the conditions, especially the solvent composition [15]. In solution chemistry the removal of trityl protection is sometimes problematic, since the cleavage reaction via a trityl cation may become reversible, leading to an equilibrium between the protected and deprotected substrates and thus incomplete deprotection [16]. In those cases an acetal protection may offer a more useful solution.

**Table 1:** Half-lives of 5’-acetal (**4a–e**) and 3’-acetal-protected (**7a,c–e**) 2’-deoxythymidines, and 2’-deoxy-3’-*O*-propen-2-ylthymidine (**8b**) in acetate (pH 4.94), citrate (pH 5.61) and phosphate (pH 6.82) buffers at 25.0 °C. The ionic strength was adjusted to 0.3 M with KCl and detailed kinetic data with statistical error limits are given in Supporting Information File 1.

Compound	*t*_1/2_		

	pH 4.94	pH 5.61	pH 6.82

**4a**	6 min	28 min	7 h
**4b**	10 min	50 min	12 h
**4c**	48 s	3 min	1 h
**4d**	50 s	4 min	52 min
**4e**	3 h	14 h	8 days
**7a**	14 min	45 min	11 h
**7c**	57 s	5 min	1 h
**7d**	1.4 min	6 min	1.5 h
**7e**	6 h	27 h	16 days
**8b**	15 min	18 min	1.6 h

We verified also the rapid cleavage of the acetal protections in organic solvents. All the acetals studied were readily removed under the conditions typically applied in oligonucleotide synthesis, i.e., 3% dichloroacetic acid in acetonitrile or in methanol. In most cass the reactions were complete after a couple of minutes. With the trifluoroethyl acetal derivative **4e** the reaction was slow enough that the half-life could be measured, and it was shown to be 4 minutes in methanol and 7 minutes in acetonitrile. The polarity of the solvent has a significant effect on the stability. Slower reactions were observed using 3% acetic acid in dioxane, where the half-lives for **4a** and **4c** were 80 minutes and 5 minutes, respectively. These results indicate that the proposed protecting groups can be applied, for instance, in solution-phase oligonucleotide synthesis without depurination becoming a major problem. Further studies of the applications will be published later.

### Properties of the protections

The recovery of the 5’-acetal-protected nucleoside from aqueous work-up after ammonolysis of the 3’-*O*-benzoyl protecting groups correlates inversely to the size, and hence the hydrophobicity of the acetal protection. The MIP-protected compound **4a** gives 83% isolated yield whereas the cyclohexyloxy acetal-protected derivative **4c** gives a quantitative isolated yield. The isolated yield of the isopropyloxy acetal-protected compound **4d** lies between these giving still good 89% recovery. The 2-isopropyloxypropan-2-yl acetal is as easily removed as the cyclohexyl derivative, but gives volatile hydrolysis products, isopropanol and acetone, thus retaining this advantage of MIP-protection. On the other hand, the 2-cyclohexyloxypropan-2-yl substituent gives the benefit of better solubility in organic solvents.

## Conclusion

The hydrolytic stability of five different “acetone-based” acetal protecting groups, i.e., 2-alkoxypropan-2-yl groups introduced at the 5’- and 3’-hydroxy groups of a 2’-deoxynucleoside were determined. The systematic quantitative reactivity data help to select a suitable protecting group, when a highly acid-labile protection for a primary or secondary hydroxy group is needed. The protecting reagents are readily prepared, introduced on nucleoside hydroxy gropus in a few minutes’ reaction, and the isolation of the protected compounds proceeds smoothly. The variation of the structure of the protecting groups allows steering of the reactivities over a wide range, and also solubility effects can be considered.

## Supporting Information

File 1 Experimental details and analytical data.

File 2 Copies of ^1^H NMR and ^13^C NMR spectra.
