# Supplementary material for: Tuning the stability of alkoxyisopropyl protection groups
Source: Beilstein J Org Chem. 2019 Mar 21;15:746–51. doi: 10.3762/bjoc.15.70 (PMC6444389; doi:10.3762/bjoc.15.70)
Supplement: File 1 — Experimental details and analytical data. [file Beilstein_J_Org_Chem-15-746-s001.pdf]

BEILSTEIN JOURNAL OF ORGANIC CHEMISTRY

Supporting Information

for

Tuning the stability of alkoxyisopropyl protection groups

Zehong Liang, Henna Koivikko, Mikko Oivanen and Petri Heinonen

Beilstein J. Org. Chem. **2019,** *15,* 746–751. doi:10.3762/bjoc.15.70

Experimental details and analytical data

License and Terms: This is a supporting information file under the terms of the Creative Commons Attribution License (http://creativecommons.org/licenses/by/4.0). Please note that the reuse, redistribution and reproduction in particular requires that the authors and source are credited.
The license is subject to the *Beilstein Journal of Organic Chemistry* terms and conditions: (https://www.beilstein-journals.org/bjoc)

Table of Contents

1. General methods S1

2. Experimental details and analytical data...S1

3. References..S14

4. Appendix for SI: Calculation of first order rate constants by linear regression analysis of the chromatographic data..S15

1. General methods

The NMR spectra were recorded on Varian INOVA 500 MHz, Varian Gemini 300 MHz, Bruker Avance Neo 500 MHz or Bruker Avance Neo 600 MHz NMR spectrometers. The chemical shifts are given in ppm using the solvent signal for calibration of the ppm scale. The high-resolution mass spectra were recorded on a Bruker Micro-TOF mass spectrometer with ESI-ionization or JEOL JMS-700 spectrometer with EI-ionization (compound **1e**).

2. Experimental details and analytical data

Kinetic methods.

The composition of samples withdrawn from reaction solutions at appropriate intervals were determined by RP HPLC. The reaction vials were incubated in a water bath, temperature of which was maintained at 25.0 °C ± 0.1 °C. The buffer solutions of known pH and concentration of 50 mM were prepared from acetic acid and sodium acetate (pH 4.94), citric acid partially neutralized with KOH (pH 5.61) and potassium dihydrogen phosphate partially neutralized with KOH (pH 6.82). The ionic strength of the solutions was adjusted with KCl to 0.30 M. The kinetic measurement was started by mixing the substrate sample (200 μL in DMSO) into the pre-thermostated reaction

solution (5 mL). The initial substrate concentration in the reaction solutions was 0.15 mg/mL. The first sample was withdrawn immediately after the mixing. The hydrolysis reaction was quenched by pipetting the withdrawn sample onto 50 mM sodium bicarbonate solution kept in an ice bath. The RP HPLC analyses were performed using commercial ODS Hypersil (250 x 4 mm, 5μ) column eluted with an isocratic mixture of acetonitrile and 0.050 M phosphate buffer at pH 7.00. The acetonitrile percentage was adjusted for each studied compound so that the total HPLC analysis time of a sample was around 10 min.

Table SI-1. First-order rate constants for hydrolysis of the acetal derivatives of 2'-deoxythymidines **4a–e**, **7a,c–e** and the enol ether derivative **8b** in pH region 4.92–6.82 at 25.0 °C. The ionic strength of the solutions was adjusted to 0.3 M with KCl.

Compound	k_{obs} /s^{-1}		
	pH 4.94	pH 5.61	pH 6.82
**4a**	$(1.89 \pm 0.01) \cdot 10^{-3}$	$(4.08 \pm 0.04) \cdot 10^{-4}$	$(2.72 \pm 0.02) \cdot 10^{-5}$
**4b**	$(1.20 \pm 0.02) \cdot 10^{-3}$	$(2.42 \pm 0.02) \cdot 10^{-4}$	$(1.57 \pm 0.02) \cdot 10^{-5}$
**4c**	$(1.45 \pm 0.02) \cdot 10^{-2}$	$(3.50 \pm 0.07) \cdot 10^{-3}$	$(2.05 \pm 0.02) \cdot 10^{-4}$
**4d**	$(1.40 \pm 0.02) \cdot 10^{-2}$	$(3.23 \pm 0.03) \cdot 10^{-3}$	$(2.21 \pm 0.03) \cdot 10^{-4}$
**4e**	$(6.03 \pm 0.17) \cdot 10^{-5}$	$(1.55 \pm 0.04) \cdot 10^{-5}$	$(9.9 \pm 1.2) \cdot 10^{-7}$
**7a**	$(8.38 \pm 0.17) \cdot 10^{-4}$	$(2.58 \pm 0.07) \cdot 10^{-4}$	$(1.80 \pm 0.01) \cdot 10^{-5}$
**7c**	$(1.22 \pm 0.03) \cdot 10^{-2}$	$(2.25 \pm 0.05) \cdot 10^{-3}$	$(1.66 \pm 0.03) \cdot 10^{-4}$
**7d**	$(8.45 \pm 0.08) \cdot 10^{-3}$	$(1.92 \pm 0.03) \cdot 10^{-3}$	$(1.33 \pm 0.04) \cdot 10^{-4}$
**7e**	$(3.23 \pm 0.10) \cdot 10^{-5}$	$(7.07 \pm 0.10) \cdot 10^{-6}$	$(5.0 \pm 0.4) \cdot 10^{-7}$
**8b**	$(7.51 \pm 0.10) \cdot 10^{-4}$	$(6.49 \pm 0.04) \cdot 10^{-4}$	$(1.19 \pm 0.02) \cdot 10^{-4}$

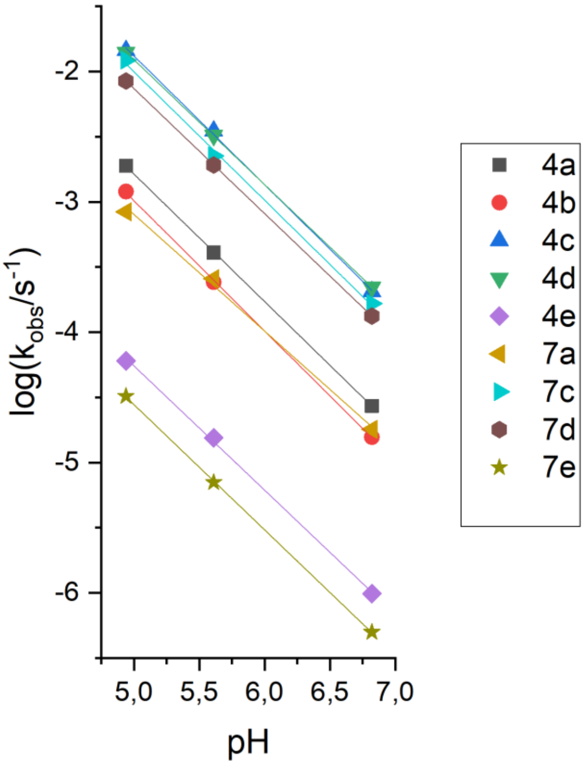

Figure S1. pH–rate profiles for the acetal hydrolysis of **4a–e**, **7a**, and **7b–e**. For conditions see Table S1.

Synthesis of compounds and analytical data.

Compound **1a** was purchased from Sigma-Aldrich. Compounds **1b** [1], and **1c** and **1d** [2] were prepared according to the published methods.

*Synthesis of the protecting group **1e**.*

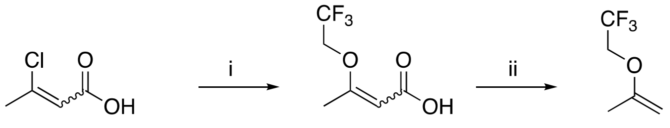

Scheme S1. Synthesis of 2-trifluoroethoxypropene **1e**. (i) CF_3CH_2ONa, THF; (ii) heating.

3-(2,2,2-Trifluoroethoxy)but-2-enoic acid (**10**). To the solution of 2,2,2-trifluoroethanol (14.0 ml, 0.140 mol) in dry THF (100 ml) was added NaH (7.0 g, 0.292 mol) in small portions. A mixture of (*E*/*Z*)-3-chlorobut-2-enoic acid (**9**) and 3,3-dichlorobutanoic acid (7.71 g, containing 5.94 g, 0.049 mol compound **9**) [2] was added dissolved in dry THF. The reaction mixture was refluxed under argon for 18 h and the reaction was monitored by ^{19}F NMR. Water (150 ml) was carefully added and the mixture was washed with ether. The aqueous phase was acidified with conc. aqueous HCl and the product extracted with ether (3 x 150 ml). The ethereal phases were combined, dried over $MgSO_4$ and the solvent removed by rotary evaporation. The white precipitate dried in high vacuum (9.06 g, 0.049 mol) was used without further purification. An analytical sample was recrystallized from methanol. Melting point 116.7 °C. ^1H-NMR (500 MHz, DMSO-d_6) δ 11.80 (s, 1H, COOH), 5.13 (s, 1H), 4.56 (q, $J_{F\text{-}H}$ = 8.7 Hz, 2H, OC**H**$_2$CF$_3$), 2.22 (s, 3H, CH_3). ^{13}C NMR (75 MHz, DMSO-d_6) δ 168.78, 167.79, 123.5 (q, $J_{C\text{-}F}$ = 276 Hz), 94.04, 64.26 (q, $J_{C\text{-}F}$ = 35 Hz), 17.78. HRMS (ESI -): $[M\text{-}H^+]^-$ calculated for $C_6H_6F_3O_3^-$ 183.0275, found 183.0279.

2-(2,2,2-Trifluoroethoxy)prop-1-ene (**1e**). Compound **10** (3.45 g, 18.7 mmol) was placed in a micro distillation apparatus connected to an argon flow. The distillation flask was heated with a heating gun (300 °C) and the product collected distilling at 149 °C. Yield 2.24 g, 16 mmol, 86 %. ^{1}H-NMR (500 MHz, DMSO-d_6) δ 4.33 (q, $J_{F\text{-}H}$ = 8.5 Hz, 2H, OCH_2CF_3), 4.05 (m, 2H, =CH_2), 1.80 (s, 3H, CH_3). ^{13}C-NMR (125 MHz, DMSO-d_6) δ 157.7, 123.9 (q, $J_{F\text{-}C}$ = 276 Hz), 84.0, 64.0 (q, $J_{F\text{-}C}$ = 35 Hz), 19.8. ^{19}F-NMR (282 MHz, DMSO-d_6): δ -73.7 (t, $J_{F\text{-}H}$ = 8.5 Hz). HRMS (EI+): $[M]^+$ calculated for $C_5H_7F_3O^+$ 140.0449 found 140.0451.

Synthesis of the protected nucleosides.

3'-O-Benzoyl-5'-O-(2-methoxypropan-2-yl)-2'-deoxythymidine (**3a**). Compound **2** (0.50 g, 1.45 mmol) was dissolved in dry THF (10 ml) and 2-methoxypropene (Sigma-Aldrich, 0.97 ml, 10.2 mmol) was added. *p*-Toluenesulphonic acid monohydrate (0.5 mol %, 0.0073 ml, 0.1 M solution) was added dissolved in dry THF. The reaction was stopped after 4 min by addition of triethylamine (1 ml). The reaction mixture was diluted with dichloromethane (100 ml) and washed with aqueous $NaHCO_3$ (2 x 50 ml) and aqueous NaCl (50 ml). The combined aqueous phases were extracted with DCM (50 ml). The combined organic phases were dried over Na_2SO_4 and the solvent was removed by rotary evaporation. The silica gel chromatography (0–4% methanol in DCM containing 0.1% TEA) yielded 0.49 g (81%) of **3a** as solid foam. ^{1}H-NMR (300 MHz, $CDCl_3$): δ 8.13 (b, 1H, NH), 8.05 (m, 2H, Bz-*o*), 7.68 (m, 1H, H6), 7.61 (m, 1H, Bz-*p*). 7.48 (m, 2H, Bz-*m*), 6.50 (dd, J = 14.5 Hz, 3.3 Hz, 1H, H1′), 5.55 (td, J = 6.0 Hz, 3.3 Hz, 1H, H3′), 4.37 (m, 1H, H4′), 3,78 (m, 2H, H5′/5''), 3.28 (s, 3H, MIP-OMe), 2.60-2.52 (m, 1H, H2′), 2.38-2,27 (m, 1H, H2′′), 1.96 (d, J = 1.2 Hz, 3H, dTMe), 1.44 (s, 6H, 2*Me in ketal). ^{13}C-NMR (125 MHz, $CDCl_3$) δ 166.4, 164.1, 150.8, 135.6, 133.9, 130.0, 129.4, 128.8, 111.5, 100.7, 85.0, 84.3, 76.3, 61.5, 49.2, 38.3, 24.8, 24.7, 12.8. HRMS (ESI +): $[M+Na]^+$ calculated for $C_{21}H_{26}N_2O_7Na^+$ 441.1632 found 441.1619.

3'-O-Benzoyl-5'-O-(2-benzyloxypropan-2-yl)-2'-deoxythymidine (**3b**). Was prepared from **2** and **1b** (7 equiv) as described for **3a**. Yield 70.3%. ^{1}H-NMR (500 MHz, DMSO-d_6) δ 11.4 (s, 1, NH), 8.00 (m, 2H, Bz-*o*), 7.69 (m, 1H, Bz-*p*), 7.63 (s, 1H, H6), 7.54 (m, 2H, Bz-*m*), 7.3-7.2 (m, 5H, Bn-*o*, Bn-*p*, Bn-*m*), 6.30 (m, 1H, H1'), 4.46 (s, 2H, Bn-CH_2), 5.49 (m, 1H, H3'), 4,30 (m, 1H, H4'), 3.74 (m, 2H, H5'/5''), 2.50 (m, 2H, H2'/2''), 1.78 (s, 3H, dTMe), 1.42 (2*s, 6H, 2*Me in ketal). ^{13}C-NMR (125 MHz, DMSO-d_6): δ 165.2, 163.6, 150.4, 138.5, 135.6, 133.6, 129.3, 128.7,128.1, 127.5, 127.2, 109.7, 100.2, 83.9, 82.6, 75.3, 62.6, 61.1, 36.4, 24.7 (2 peaks), 12.1. HRMS (ESI +): $[M+Na]^+$ calculated for $C_{27}H_{30}N_2O_7Na^+$ 517.945 found 517.1952.

3'-O-Benzoyl-5'-O-(2-cyclohexyloxypropan-2-yl)-2'-deoxythymidine (**3c**). Was prepared from **2** and **1c** (7 equiv) as described for **3a**. Yield 78%. ^{1}H-NMR (500 MHz, Acetone-d_6): δ 9.99 (bs, 1H, NH), 8.09 (m, 2H, Bz-*o*), 7.73 (m, 1H, H6), 7.67 (m, 1H, Bz-*p*), 7.54 (m, 2H, Bz-*m*), 6.45 (m, 1H, H1'), 5.58 (m, 1H, H3'), 4.38 (m, 1H, H4'), 3.82-3.92 (m, 2H, H5'/H5''), 3.76 (m, 1H, Chx), 2.54 (m, 2H, H2'/H2''), 1.89 (m, 5H, dTMe and Chx), 1.68 (m, 2H, Chx), 1.44-1.51 (m, 7H, 2*Me in ketal and Chx), 1.10-1.35 (m, 5H, Chx). ^{13}C-NMR (125 MHz, Acetone-d_6): δ 165.7, 163.5, 150.6, 135.7, 130.2, 129.7, 128.8, 110.3, 100.7, 84.6, 84.0, 76.1, 69.5, 61.5, 37.6, 34.7, 34.7, 25.8, 25.7, 25.6, 24.5, 24.4, 12.1. HRMS (ESI +): $[M+Na]^+$ calculated for $C_{26}H_{34}N_2O_7Na^+$ 509.2258 found 509.2239.

3'-O-Benzoyl-5'-O-(2-isopropyloxypropan-2-yl)-2'-deoxythymidine (**3d**). The compound was prepared from **2** and **1d** (7 equiv) as described for **3a**. Yield 74%. ^{1}H-NMR (500 MHz, DMSO-d_6) δ 11.37 (s, 1H, NH), 8.02 (m, 2H, *o*-Bz), 7.70 (m, 1H, *p*-Bz), 7.62 (m, 1H, H6), 7.55 (m, 2H, *m*-Bz), 6.29 (m, 1H, H1'), 5.45 (m, 1H, H3'), 4.28 (m, 1H, H4'), 3.97 (m, 1H, C**H**Me_2), 3.71 (m, 2H, H5'), 2.44 – 2.51 (m, 2H, H2', overlapping with DMSO signal), 1.80 (m, 3H, dTMe), 1.34 (2*s,

6H, 2*Me), 1.08 (m, 6H, CH(C**H**$_3$)$_2$). ^{13}C-NMR (125 MHz, DMSO-d_6) δ 165.2, 163.6, 150.4, 135.6, 133.6, 129.4, 129.3, 129.2, 128.8, 109.6, 100.1, 83.9, 82.9, 75.4, 62.8, 61.2, 36.5, 25.6, 25.5, 24.2, 12.2. HRMS (ESI +): $[M+Na]^+$ calculated for $C_{23}H_{30}N_2O_7Na^+$ 469.1945 found 469.1943.

3'-O-Benzoyl-5'-O-(2-(2,2,2-trifluoroethoxy)propan-2-yl)-2'-deoxythymidine (**3e**). The compound was prepared from **2** and **1e** (7 equuiv) as described for **3a** except the reaction time was over night. Yield 35%. ^{1}H-NMR (500 MHz, DMSO-d_6): δ 11.36 (s, 1H, NH), 8.9 (m, 2H, Bz-*o*), 7.68 (m, 1H, Bz-p), (7.55, m, 3H, Bz-m and H6), 6.27 (dd, J = 8.1, 6.3 Hz, 1H, H1'), 5.45 (m, 1H, H3'), 4.26 (m, 1H, H4'), 3.99 (m, 2H, CF_3CH_2O), 3.72 (m, 2H, H5'/H5''), 2.41-2.56 (m, 2H, H2'/H2''), 1.80 (s, 3H, dTMe), 1.38 (s, 6H, 2*Me in ketal). ^{13}C-NMR (125 MHz, DMSO-d_6): δ 165.2, 163.6, 150.4, 135.7, 133.6, 129.3, 128.7, 124.6 (q, $J_{C\text{-}F}$ = 278 Hz), 109.7, 101.0, 83.8, 82.2, 74.9, 61.4, 58.7 (q, $J_{C\text{-}F}$ = 32 Hz), 36.1, 24.3, 24.2, 12.1. ^{19}F-NMR (282 MHz, DMSO-d_6): δ -73.0 (t, $J_{F\text{-}H}$ = 9.3 Hz). HRMS (ESI +): $[M+H]^+$ calculated for $C_{22}H_{25}N_2O_7H^+$ 487.1687 found 487.1697.

5'-O-(2-Methoxypropan-2-yl)-2'-deoxythymidine (**4a**). **3a** (2.7 g, 6.45 mmol) was dissolved in methanol (100 ml) and concentrated aqueous ammonia (100 ml) was added. The reaction mixture was stirred overnight, concentrated and evaporated several times with ethanol and toluene to remove ammonia and water. Purification by silica gel chromatography (3–5% methanol in DCM containing 0.1% TEA) yielded 1.68 g (83 %) of **4a** as a solid foam. ^{1}H-NMR (500 MHz, DMSO-d_6): δ 11.28 (s, 1H, NH), 7.54 (m, 1H, H5), 6.17 (m, 1H, H1), 5.28 (d, J = 4.3 Hz, 1H, 3'OH), 4.24 – 4.17 (m, 1H, H3'), 3.86 (m, 1H, H4'), 3.50 (m, 2H, H5'/H5''), 3.09 (s, 3H, MeO), 2.17 – 2.02 (m, 2H, H2'/H2''), 1.77 (d, J = 1.2 Hz, 3H, dTMe), 1.28 (s, 6H, 2*Me). ^{13}C-NMR (125 MHz, DMSO-d_6): δ 163.6, 150.4, 135.7, 109.3, 99.6, 85.4, 83.7, 70.7, 60.8, 47.9, 24.2, 24.2, 12.1. HRMS (ESI +): $[M+Na]^+$ calculated for $C_{14}H_{22}N_2O_6Na^+$ 337.1370 found 337.1365. Spectroscopic data of the compound match with the data reported earlier. [3]

5'-O-(2-Benzyloxypropan-2-yl)-2'-deoxythymidine (**4b**). The compound was prepared from **3b** as described for **4a**. Yield 98%. ^{1}H-NMR (500 MHz, DMSO-d_6): δ 11,28 (s, 1H, NH), 7.53 (m, 1H, H6), 7.31 (m, 2H, Bn-*o*), 7.31 (m, 1H, Bn-*p*), 7.24 (m, 2H, Bn-*m*), 6.18 (m 1H, H1'), 5.30 (d, *J*=5.0 Hz, 1H, 3'-OH), 4.43 (s, 2H, Bn-CH_2), 4.26 (m, 1H, H3'), 3.89 (m, 1H, H4'), 3.62 (m, 2H, H5'/5''), 2.13 (m, 2H, H2'/2''), 1.76 (s, 3H, dTMe), 1.39 (2*s, 6H, 2*Me). ^{13}C-NMR (125 MHz, DMSO-d_6): δ 163.6, 150.4, 138.6, 135.8, 128.1, 127.4, 127.1, 109.5, 100.0, 85.2, 83.6, 70.5, 62.4, 61.0, 39.2, 24.8, 24.7, 12.1. HRMS (ESI +): $[M+Na]^+$ calculated for $C_{20}H_{26}N_2O_6Na^+$ 413.1683 found 413.1696.

5'-O-(2-Cyclohexyloxypropan-2-yl)-2'-deoxythymidine (**4c**). Was prepared from **3c** as described for **4a**. Yield: quantitative. ^{1}H-NMR (500 MHz, DMSO-d_6): δ 11.27 (s, 1H, NH), 7.49 (s, 1H, H6), 6.16 (m, 1H, H1'), 5.27 (d, *J*=5.0 Hz, 1H, 3'-OH), 4.21 (m, 1H, H3'), 3.81 (m, 1H, H4'), 3.63-3.50 (m, 3H, H5'/5'' and Chx), 2.14 (m, 2H, H2'/2''), 1.79 (m, 5H, dTMe and Chx), 1.6 (m, 2H, Chx), 1.45 (m, 1H, Chx), 1.31 (s, 6H, 2*Me), 1.19-1.09 (m, 5H, Chx). ^{13}C-NMR (75 MHz, DMSO-d_6): δ 163.6, 150.4, 135.9, 109.3, 99.9, 85.2, 83.4, 70.3, 68.7, 60.9, 34.2, 25.7, 25.6, 25.1, 24.1, 12.2. HRMS (ESI +): $[M+Na]^+$ calculated for $C_{19}H_{30}N_2O_6Na^+$ 405.1996 found 405.1994.

5'-O-(2-Isopropyloxypropan-2-yl)-2'-deoxythymidine (**4d**). The compound was prepared from **3d** as described for **4a**. Yield 89%. ^{1}H-NMR (600 MHz, DMSO-d_6) δ 11.29 (s, 1H, NH), 7.51 (m, 1H, H6), 6.15 (m, 1H, H1'), 5.30 (d, *J* = 4.4 Hz, 1H, 3'OH) 4.21 (m, 1H, H3'), 3,93 (m, 1H, C**H**Me_2), 3.83 (m, 1H, H4'), 3.50 – 3.60 (m, 2H, H5'), 2.05 – 2.15 (m, 2H , H2'), 1.78 (m, 3H, dTMe), 1.30 (s, 6H, 2*Me), 1.08 (m, 6H, CH(C**H**$_3$)$_2$). ^{13}C-NMR (150 MHz, DMSO-d_6): δ 163.6, 150.4, 135.8, 109.3, 99.9, 85.4, 83.6, 70.6, 62.6, 61.1, 39.3*, 25.6, 25.6, 24.3, 24.2, 12.1. *C2' (39.3 ppm) under

the DMSO-d_6 signal verified by HSQC-spectrum. HRMS (ESI +): $[M+Na]^+$ calculated for $C_{16}H_{26}N_2O_6Na^+$ 365.1683 found 365.1697.

5'-O-(2-(2,2,2-Trifluoroethoxy)propan-2-yl)-2'-deoxythymidine (**4e**). The compound was prepared from **3e** as described for **4a**. Yield 67%. ^{1}H-NMR (500 MHz, DMSO-d_6): δ 11.28 (s, 1H, NH), 7.45 (m, 1H, H6), 6.16 (m, 1H, H1), 5.30 (d, J = 4.5 Hz, 1H, 3'OH), 4.24 – 4.19 (m, 1H, H3'), 3.98 – 3.90 (m, 2H, CF_3*CH_2*O), 3.84 - 3.80 (m, 1H, H4'), 3.63-3.50 (m, 2H, H5'/H5''), 2.20 – 2.05 (m, 2H, H2'/H2''), 1.77 (d, J = 1.2 Hz, 3H, Me), 1.36 (s, 6H, 2*Me). ^{13}C-NMR (125 MHz, DMSO-d_6): δ 163.6, 150.4, 135.8, 124.7 (q, J_{C-F} = 276 Hz), 109.5, 100.7, 84.8, 84.5, 70.3, 61.4, 58.6 (q, J_{C-F} = 34 Hz), 38.9, 24.3, 24.2, 12.1. ^{19}F-NMR (282 MHz, DMSO-d_6): δ -72.9 (t, J_{F-H} = 9.4 Hz). HRMS (ESI +): $[M+Na]^+$ calculated for $C_{15}H_{21}F_3N_2O_6Na^+$ 405.1244 found 405.1226.

3'-O-(2-Methoxypropan-2-yl)-5'-O-tert-butyldimethylsilyl-2'-deoxythymidine (**6a**). 5'-*tert*-Butyldimethylsilyl-2'-deoxythymidine, (**5**, 200 mg, 0.56 mmol) was dissolved in dry THF (2 mL). Then 2-methoxypropene (Sigma-Aldrich, 0.376 ml, 3.9 mmol, 7 equiv) was added, followed by *p*-toluenesulphonic acid monohydrate (28 μL, 0.1 M solution in dry THF, 2.8 μmol, 0.5 mol %). The reaction was monitored by TLC and when completed, triethylamine (0.2 ml) and dichloromethane (50 ml) were added. The mixture was washed with aqueous $NaHCO_3$ (2 x 25 ml) and brine (25 ml). The combined aqueous phases were extracted with DCM (25 ml). The combined organic phases were dried over Na_2SO_4 and the solvent was removed by rotary evaporation. The silica gel chromatography (20% EtOAc to 50% EtOAc in hexane containing 0.1% TEA) yielded 233 mg (97%) of **6a** as solid foam. ^{1}H-NMR (500 MHz, DMSO-d_6): δ 11.32 (s, 1H, NH), 7.42 (m, 1H, H6), 6.12 (m, 1H, H1'), 4.33 (m, 1H, H3'), 3.91 (m, 1H, H4'), 3.70-3.79 (m, 2H, H5'/H5''), 3.10 (s, 3H, MeO), 2.12-2.15 (m, 2H, H2'/H2''), 1.77 (d, J = 1.2 Hz, 3H, Me), 1.28 (s, 6H, 2*Me), 0.88 (s, 9H,

TBS), 0.07 (2*s, 6H, TBS). ^{13}C-NMR (125 MHz, DMSO-d_6): δ 163.5, 150.3, 135.3, 109.5, 85.3, 83.9, 70.1, 63.0, 48.2, 38.1, 25.7, 25.0, 24.9, 17.9, 12.2, -5.6, -5.6. $[M+Na]^+$ calculated for $C_{20}H_{36}N_2O_6SiNa^+$ 451.2235 found 451.2248.

3'-O-(2-Cyclohexyloxypropan-2-yl)-5'-O-tert-butyldimethylsilyl-2'-deoxythymidine (**6c**). The compound was prepared from **5** and **1c** as described for **6a**, yield 88%. ^{1}H-NMR (500 MHz, DMSO-d_6): δ 11.33 (s, 1H, NH), 7.45 (m, 1H, H6), 6.12 (dd, J = 9.1, 5.6 Hz, 1H, H1'), 4.42 (m, 1H, H3'), 3.96 (m, 1H, H4'), 3.71-3.79 (m, 2H, H5'/H5''), 3.61 (m, 1H, Chx), 2.04-2.16 (m, 2H, H2'/H2''), 1.61-1.76 (m, 7H, dTMe and Chx), 1.45 (m, 1H, Chx), 1.10-1.30 (m, 11H, 2*Me and Chx), 0.88 (s, 9H, TBS), 0.08 (2*s, 6H, TBS). ^{13}C-NMR (125 MHz, DMSO-d_6): δ 163.6, 150.4, 135.2, 109.5, 100.7, 85.9, 84.1, 70.7, 68.4, 63.2, 38.4, 34.3, 34.0, 26.6, 26.2, 25.7, 25.1, 24.0, 23.9, 18.0, 12.2, -5.5, -5.6. $[M+Na]^+$ calculated for $C_{25}H_{44}N_2O_6SiNa^+$ 519.2861 found 519.2860.

3'-O-(2-Isopropyloxypropan-2-yl)-5'-O-tert-butyldimethylsilyl-2'-deoxythymidine (**6d**). The compound was prepared from **5** and **1d** as described for **6a.** Yield 74%.

^{1}H-NMR (500 MHz, $CDCl_3$) δ 8,48 (bs, 1H, NH), 7,54 (m, 1H, H6), 6.35 (m, 1H, H1'), 4.50 (m, 1H, H3'), 4.07 (m, 1H, H4'), 4,00 (hept, *J* = 6.2 Hz, 1H, C**H**Me_2), 3.84 (m, 2H, H5'), 2.27 (m, 1H , H2'), 2.01 (m, 1H, H2''), 1.91 (m, 3H, dTMe), 1.34 (2*s, 6H, 2*Me), 1.13 (2*d, *J* = 6.1 Hz, 6H, $CH(C\mathbf{H_3})_2$), 0.92 (s, 9H, $SiC(CH_3)_3$), 0,12 (2*s, 6H, $Si(CH_3)_2$). ^{13}C-NMR (125 MHz, $CDCl_3$) δ 163.8, 150.5, 135.7, 111.0, 101.1, 87.3, 85.5, 71.5, 63.7, 63.4, 40.6, 27.1, 26.6, 26.1, 24.7, 24.5, 18.6, 12.7, -5.2, -5.3. HRMS (ESI +): $[M+H]^+$ calculated for $C_{22}H_{41}N_2O_6Si^+$ 457.2728 found 457.2741.

3'-O-(2-(2,2,2-Trifluoroethoxy)propan-2-yl)-5'-O-tert-butyldimethylsilyl-2'-deoxythymidine (**6e**). The compound was prepared from **5** and **1e** as described for **6a** except that the reaction time was 18h, yield 33%.

^{1}H-NMR (600 MHz, DMSO-d_6): δ 11.3 (bs, 1H, NH), 7.45 (m, 1H, H6), 6.12 (m, 1H, H1'), 4.38 (m, 1H, H3'), 3.93 – 4.05 (m, 3H, C**H**$_2$CF$_3$ and H4'), 3.70 – 3.78 (m, 2H, H5'/H5''), 2.12 – 2.20 (m, 2H, H2'/H2''), 1.77 (m, 3H, dTMe), 1.36 (s, 6H, 2*Me), 0.87 (s, 9H, SiC(CH$_3$)$_3$), 0.07 (2*s, 6H, Si(CH$_3$)$_2$). ^{13}C-NMR (150 MHz, DMSO-d_6): δ 164.1, 150.9, 135.8, 125.1 (q, J_{CF} = 277.5 Hz), 110.1, 102.0, 85.7, 84.4, 71.8, 63.5, 59.4 (q, J_{CF} = 33.4 Hz), 26.2, 25.5, 25.5, 18.4, 12.7, -5.1, -5.2. HRMS (ESI +): [M+H]$^+$ calculated for $C_{21}H_{36}F_3N_2O_6Si^+$ 497.2289 found 497.2306.

3'-O-(2-Methoxypropan-2-yl)-2'-deoxythymidine (**7a**). Compound **6a** (0.377 g, 0.88 mmol) was dissolved in dry THF (20 mL) and TBAF (0.53 g, 2.0 mmol) was added. The reaction was monitored by TLC and when completed (1.5 h), dichloromethane (150 ml) was added. The mixture was washed with aqueous NaHCO$_3$ (2 x 50 ml) and aqueous NaCl (50 ml). The combined aqueous phases were extracted with DCM (100 ml). The combined organic phases were dried over Na$_2$SO$_4$ and solvent was removed by rotary evaporation. The silica gel chromatography (from 1:1 EtOAc/hexane to EtOA containing 0.1% TEA) yielded 0.20 g (72%) of **7a** as solid foam. ^{1}H-NMR (500 MHz, DMSO-d_6): δ 11.27 (bs, 1H, NH), 7.67 (m, 1H, H6), 6.12 (m, 1H, H1'), 5.07 (t, J = 5.2 Hz, 1H, 5'OH), 4.37 (m, 1H, H3'), 43.86 (m, 1H, H4'), 3.53 – 3.87 (m, 2H, H5'/H5''), 3.09 (s, 3H, MeO), 2.08 – 2.20 (m, 2H, H2'/H2''), 1.77 (s, 3H, dTMe), 1.28 (s, 6H, 2*Me). ^{13}C-NMR (125 MHz, DMSO-d_6): 164.4, 151.2, 136.6, 110.1, 101.1, 86.6, 84.5, 70.9, 61.9, 48.9, 39.1, 25.8, 25.6, 12.9. HRMS (ESI +): [M+Na]$^+$ calculated for $C_{14}H_{22}N_2O_6Na^+$ 337.1370 found 337.1366.

3'-O-(2-Cyclohexyloxypropan-2-yl)-2'-deoxythymidine (**7c**). The compound was prepared from **6c** as described for **7a**. Yield 75%. ^{1}H-NMR (500 MHz, Acetone-d_6): δ 9.95 (bs, 1H, NH), 7.79 (m, 1H, H6), 6.29 (dd, J = 8.9 , 5.7 Hz, 1H, H1'), 4.62 (m, 1H, H3'), 4.33 (t, J = 4.9, 1H, 5'-OH), 4.02 (m, 1H, H4'), 3.80 (m, 2H, H5'/H5''), 3.70 (m, 1H, Chx), 2.15-2.31 (m, 2H, H2'/H2''), 1.81 (m, 5H, dTMe and Chx), 1,70 (m, 2H, Chx), 1.50 (m, 1H, Chx), 1.27-136 (m, 10H, 2*Me and Chx), 1.20 (m, 1H, Chx). ^{13}C-NMR (125 MHz, Acetone-d_6): δ 164.3, 151,5, 137.0, 110.8, 101.7, 87.9, 85.9, 72.2, 69.8, 62.9, 40.2, 35.5, 35.4, 27.2, 27.0, 26.4, 25.2, 25.1, 12.6. HRMS (ESI +): $[M+Na]^+$ calculated for $C_{19}H_{30}N_2O_6Na^+$ 405.1996 found 405.1993.

3'-O-(2-Isopropyloxypropan-2-yl)-2'-deoxythymidine (**7d**). The compound was prepared from **6d** as described for **7a**. Yield 85%. ^{1}H-NMR (500 MHz, Acetone-d_6) δ 10.0 (bs, 1H, NH), 7,82 (m, 1H, H6), 6.30 (m, 1H, H1'), 4.62 (m, 1H, H3'), 4,06 (m, 1H, C**H**Me$_2$) 4.04 (m, 1H, H4'), 3.82 (m, 2H, H5'), 2.19 – 2.33 (m, 2H, H2'/H2''), 1.82 (m, 3H, dTMe), 1.36 (2*s, 6H, 2*Me), 1.14 (m, 6H, CH(C**H**$_3$)$_2$). ^{13}C-NMR (125 MHz, Acetone-d_6) δ 163.4, 150.6, 136.1, 109.9, 100.7, 87.0, 85.0, 71.3, 62.8, 62.0, 39.4, 26.2, 26.0, 24.0, 23.9, 11.7. HRMS (ESI +): $[M+Na]^+$ calculated for $C_{16}H_{26}N_2O_6Na^+$ 365.1683 found 365.1671

3'-O-(2-(2,2,2-Trifluoroethoxy)propan-2-yl)-2'-deoxythymidine (**7e**). The compound was prepared from **6e** as described for **7a**. Yield 88%. ^{1}H-NMR (500 MHz, Acetone-d_6) δ 9.92 (bs, 1H, NH), 7.77 (m, 1H, H6), 6.28 (m, 1H, H1'), 4.63 (m, 1H, H3'), 4.36 (t, *J* = 5.0 Hz, 1H, 5'OH), 3.97 – 4.07 (m, 3H, OCH$_2$CF3 and H4'), 3.78 – 3.81 (m, 2H, H5'/H5"), 2.24 – 2.35 (m, 2H, H2'/H2"), 1.81 (m, 3H, dTMe), 1.45 (2*s, 6H, 2*Me). ^{13}C NMR (125 MHz, Acetone-d_6) δ 164.2, 164.1, 151.4, 151.3, 136.8, 125.7 (q, J_{CF} = 276.9 Hz), 110.8, 102.7, 87.3, 87.2, 85.6, 85.6, 72.8, 62.7, 62.6, 60.2 (q, J_{CF} = 34.1 Hz), 39.8, 25.6, 25.6, 12.6. ^{19}F NMR (470 MHz, Acetone-d_6): δ -74.5. HRMS (ESI +): $[M+Na]^+$ calculated for $C_{15}H_{21}F_3N_2O_6Na^+$ 405.1244 found 405.1244.

3'-O-Propen-2-yl-5'-O-tert-butyldimethylsilyl-2'-deoxythymidine (**8a**). The reaction of 5'-*tert*-butyldimethylsilyl-2'-deoxythymidine (**5**, 1.85 g, 5.2 mmol) in dry THF (15 mL) and 2-methoxypropene (3.5 ml, 36 mmol, 7 equiv) catalysed by *p*-toluenesulphonic acid monohydrate (364 µL, 1.0 M solution in dry THF, 0.36 mmol, 7.0 mol %) gives nearly a 1:1 mixture of **8a** and **6a**. The silica gel chromatography (60% EtOAc in hexane containing 0.1% TEA) yielded 1.0 g (48% from theoretical yield) of **8a** and 0.84 g (38%) of **6a**. Analytic data of **8a**: ^{1}H-NMR (600 MHz, DMSO-d_6): δ 11.35 (s, 1H, NH), 7.49 (m, 1H, H6), 6.12 (m, 1H, H1'), 4.55 (m, 1H, H3'), 4.04 (m, 1H, H4'), 3.97 (m, 1H, =CH_2), 3.90 (m, 1H, =CH_2), 3.76-3.83 (m, 2H, H5'/H5''), 2.25-2.28 (m, 2H, H2'/H2''), 1.77 (m, 6H, dTMe and CH_3 enol ether), 0.87 (s, 9H, TBS), 0.08 (2*s, 6H, TBS). ^{13}C-NMR (150 MHz, DMSO-d_6): δ 164.1, 157.5, 150.8, 135.8, 110.1, 84.5, 84.3, 84.0, 76.9, 63.7, 36.5, 26.2, 21.3, 18.4, 12.7, -5.0, -5.1. HRMS (ESI +): $[M+H]^+$ calculated for $C_{19}H_{33}N_2O_6Si^+$ 397.2153 found 397.2155.

3'-O-Propen-2-yl-2'-deoxythymidine (**8b**). The compound was prepared from **8a** as described for **7a**. Yield 81%. ^{1}H-NMR (600 MHz, DMSO-d_6) δ 11.3 (bs, 1H, NH), 7,69 (m, 1H, H6), 6.13 (m, 1H, H1'), 5.18 (t, *J*= 5.2 Hz, 1H, 5'-OH), 4.62 (m, 1H, H3'), 3.96 (m, 1H, H4'), 3.96 (m, 1H, =CH_2), 3.92 (m, 1H, =CH_2), 3.58-3.65 (m, 2H, H5'/H5''), 2.19-2.30 (m, 2H, H2'/H2''), 1.72 (m, 6H, dTMe and CH_3 enol ether), ^{13}C-NMR (151 MHz, DMSO-d_6) δ 163.7, 157.0, 150.5, 135.8, 109.6, 84.1, 83.9, 83.8, 76.6, 61.5, 36.0, 20.8, 12.2. HRMS (ESI +): $[M+H]^+$ calculated for $C_{13}H_{39}N_2O_5^+$ 283.1288 found 283.1293.

Table S2. Isolated yields of the protected 2'-deoxythymidines at the consecutive steps of the synthesis procedure. For notation, see Scheme 1.

Compound / Yield %	**3** (protection)	**6** (protection)	**4** (ammonolysis)	**7** (desilylation)
**a**	81	97	83	72
**b**	70	-	98	-
**c**	78	88	100	75
**d**	74	74	89	80
**e**	35	33	67	88

3. References

1. Mukaiyama, T.; Ohshima, M.; Murakami, M., *Chem. Lett.* **1984**, 265; Steiner, K., Graf, U., Hardegger, E., *Helv. Chim. Acta,* **1971**, *54*, 845.

2. Le Noble, W. J.; Crean, P. J., *J. Org. Chem*. **1962**, *27*, 3875.

3. Molina, A. G.; Kungurtsev, V.; Virta, P., Lönnberg, H. *Molecules* **2012**, *17*, 12102.

4. Appendix for SI: Calculation of first order rate constants by linear regression analysis of the chromatographic data

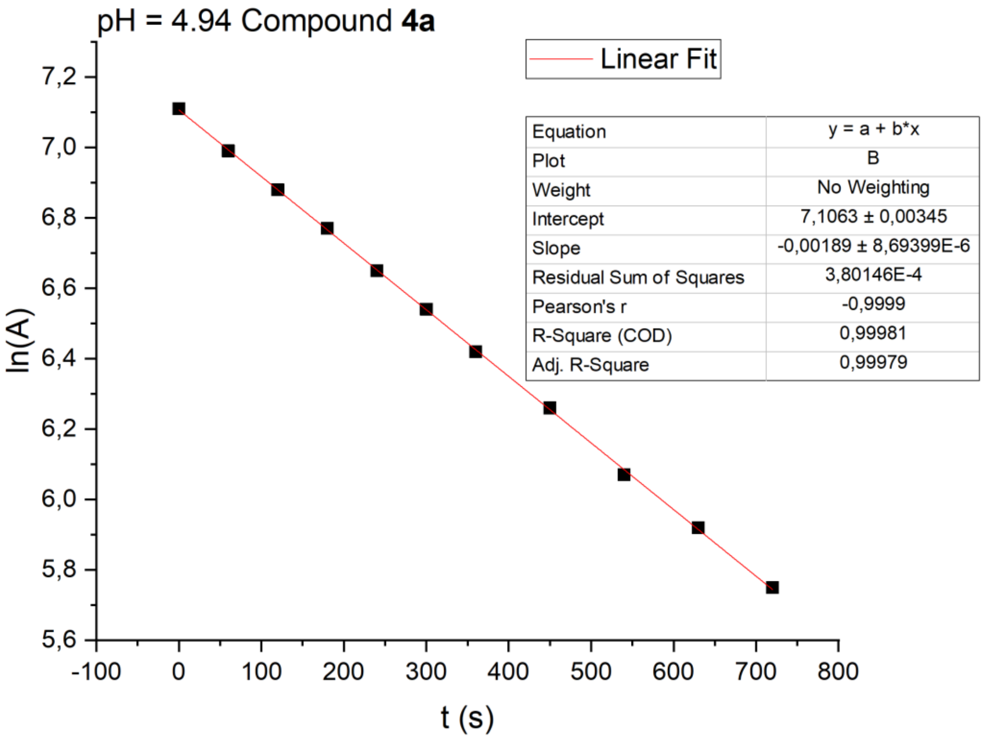

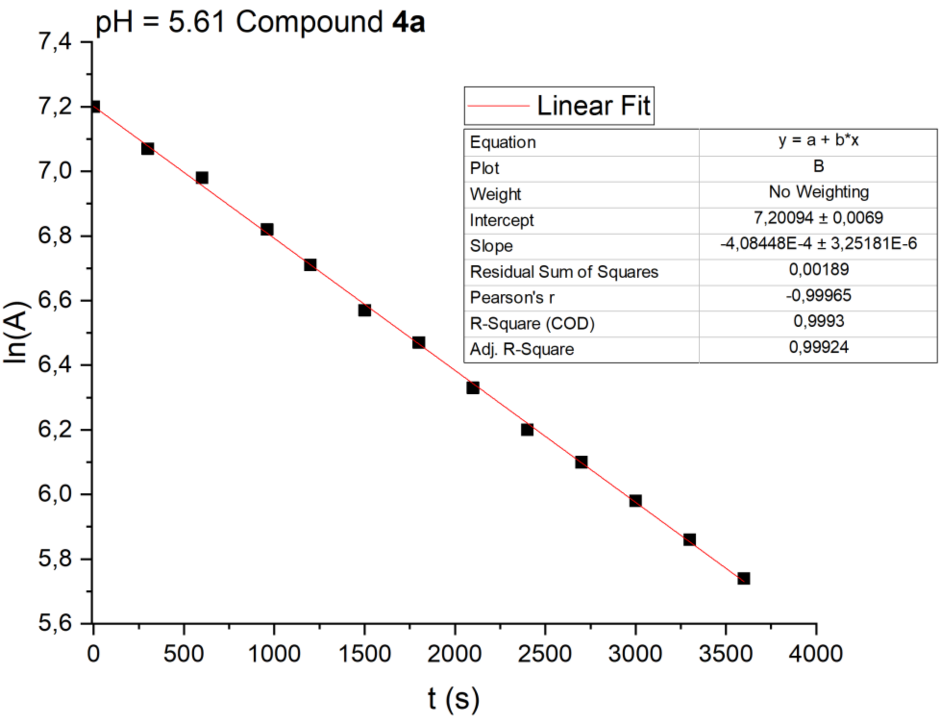

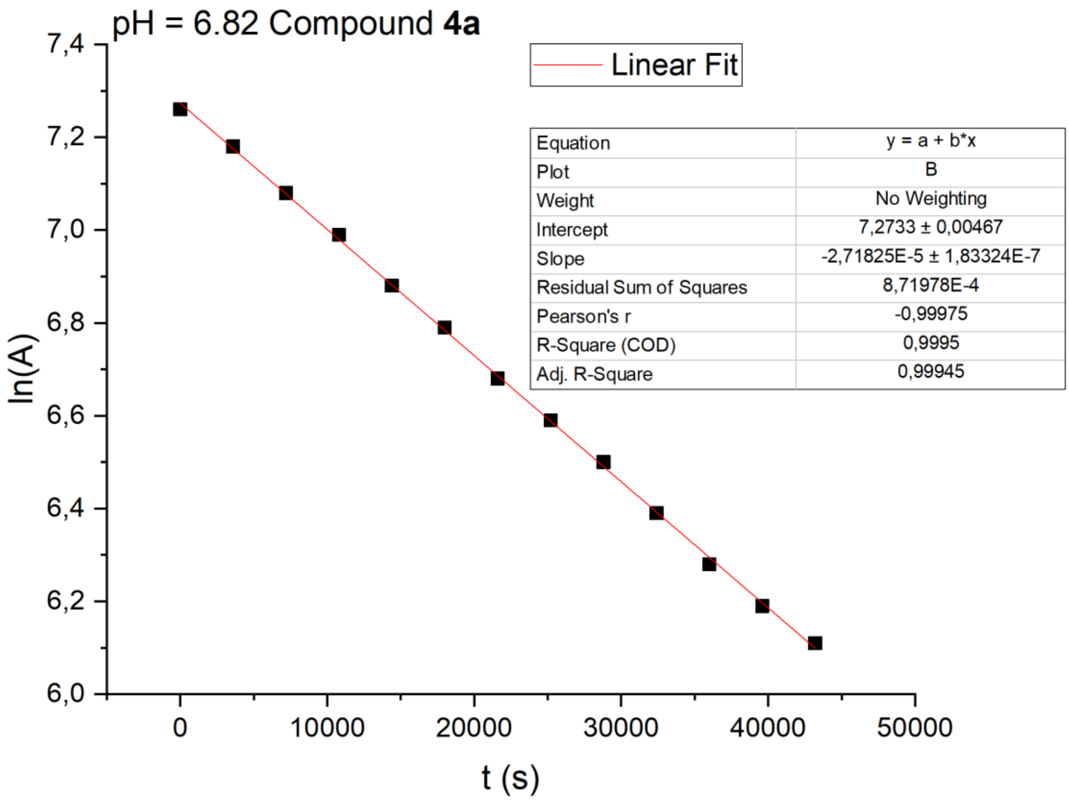
pH = 6.82 Compound 4a
Linear Fit
Equation y = a + b*x
Plot B
Weight No Weighting
Intercept 7,2733 ± 0,00467
Slope -2,71825E-5 ± 1,83324E-7
Residual Sum of Squares 8,71978E-4
Pearson's r -0,99975
R-Square (COD) 0,9995
Adj. R-Square 0,99945
ln(A)
t (s)
7,4
7,2
7,0
6,8
6,6
6,4
6,2
6,0
0
10000
20000
30000
40000
50000

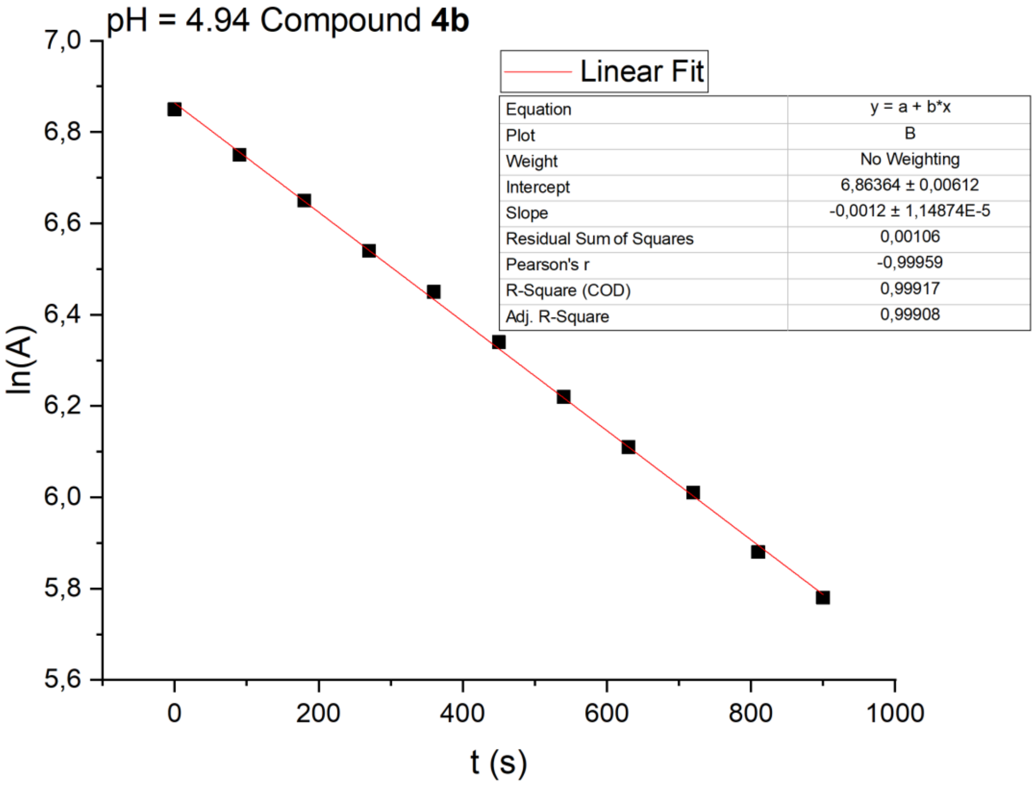
pH = 4.94 Compound 4b
Linear Fit
Equation y = a + b*x
Plot B
Weight No Weighting
Intercept 6,86364 ± 0,00612
Slope -0,0012 ± 1,14874E-5
Residual Sum of Squares 0,00106
Pearson's r -0,99959
R-Square (COD) 0,99917
Adj. R-Square 0,99908
ln(A)
t (s)
7,0
6,8
6,6
6,4
6,2
6,0
5,8
5,6
0
200
400
600
800
1000

pH = 5.61 Compound **4b**

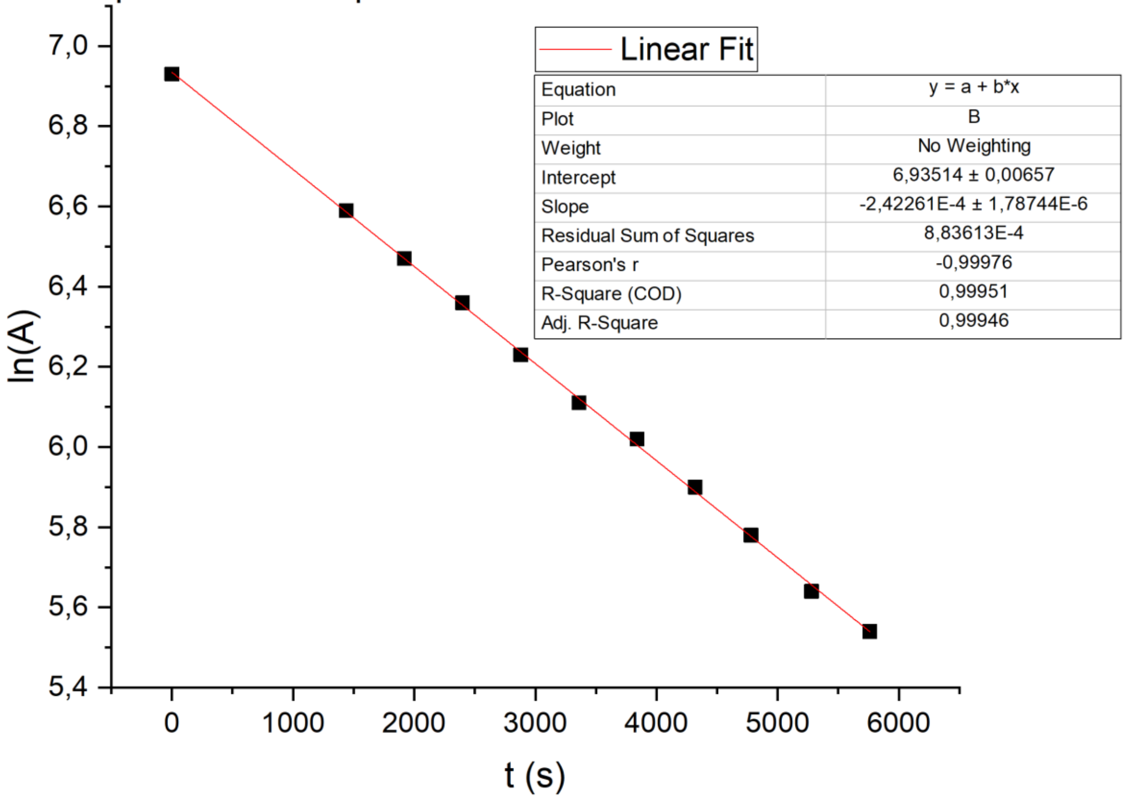

pH = 6.82 Compound **4b**

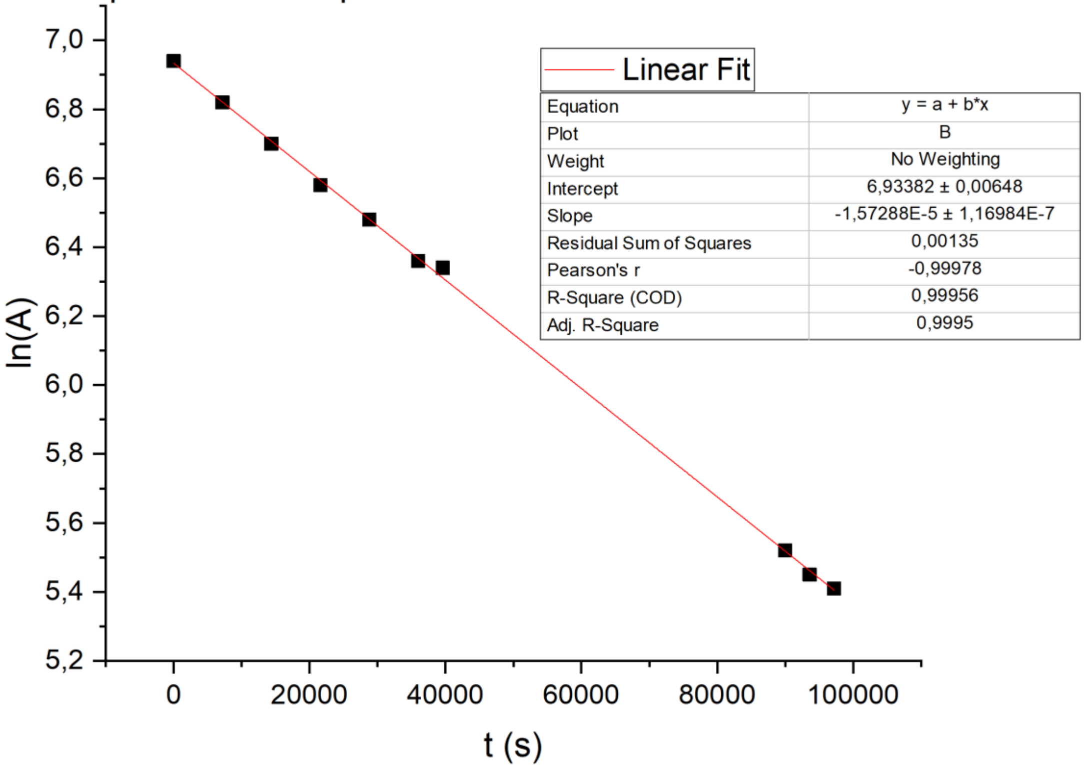

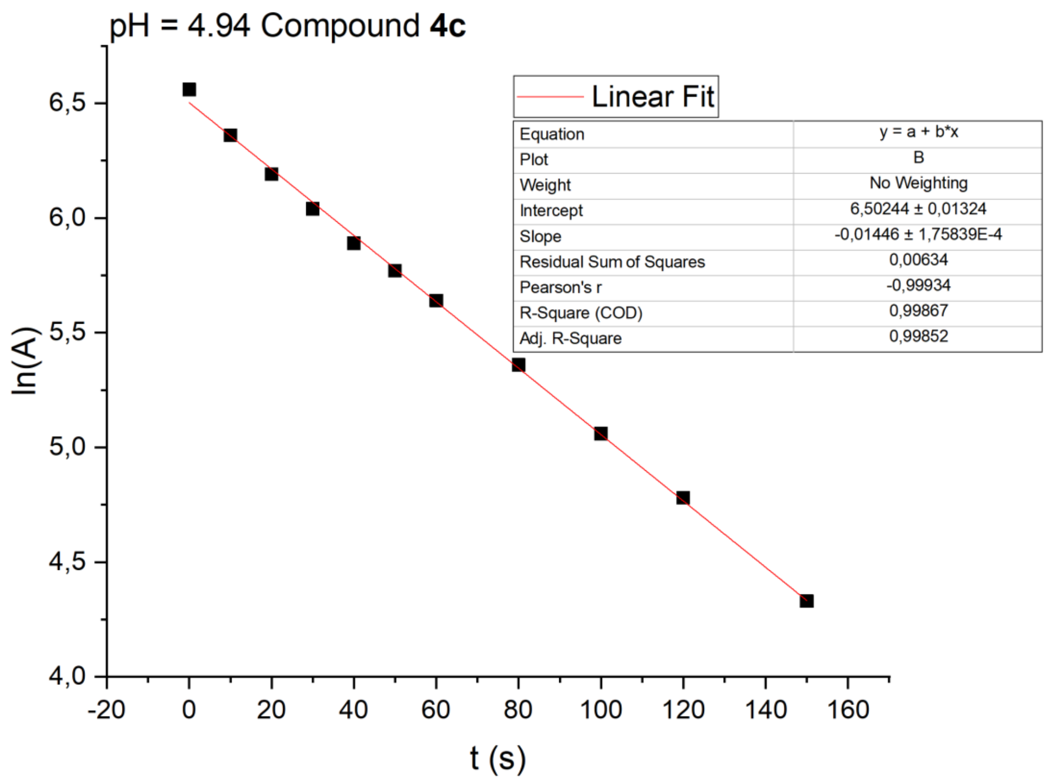
pH = 4.94 Compound 4c
Linear Fit
Equation y = a + b*x
Plot B
Weight No Weighting
Intercept 6,50244 ± 0,01324
Slope -0,01446 ± 1,75839E-4
Residual Sum of Squares 0,00634
Pearson's r -0,99934
R-Square (COD) 0,99867
Adj. R-Square 0,99852
ln(A)
t (s)
6,5
6,0
5,5
5,0
4,5
4,0
-20
0
20
40
60
80
100
120
140
160

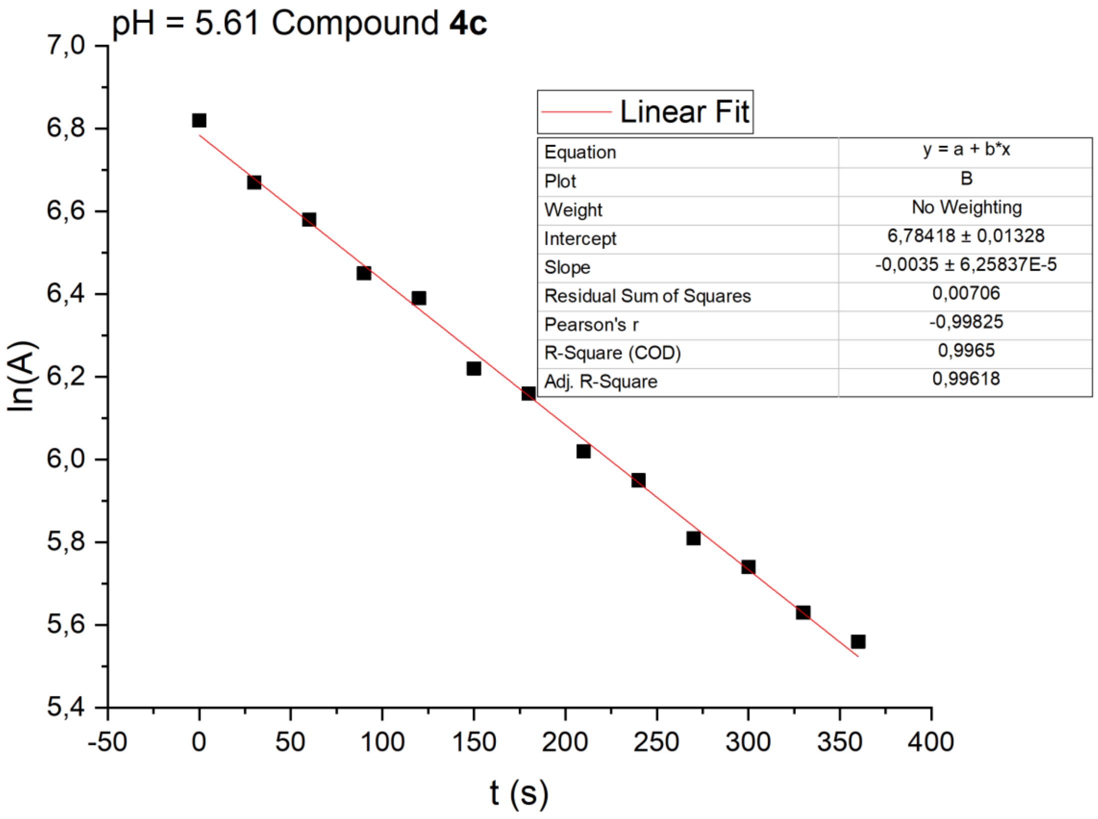
pH = 5.61 Compound 4c
Linear Fit
Equation y = a + b*x
Plot B
Weight No Weighting
Intercept 6,78418 ± 0,01328
Slope -0,0035 ± 6,25837E-5
Residual Sum of Squares 0,00706
Pearson's r -0,99825
R-Square (COD) 0,9965
Adj. R-Square 0,99618
ln(A)
t (s)
7,0
6,8
6,6
6,4
6,2
6,0
5,8
5,6
5,4
-50
0
50
100
150
200
250
300
350
400

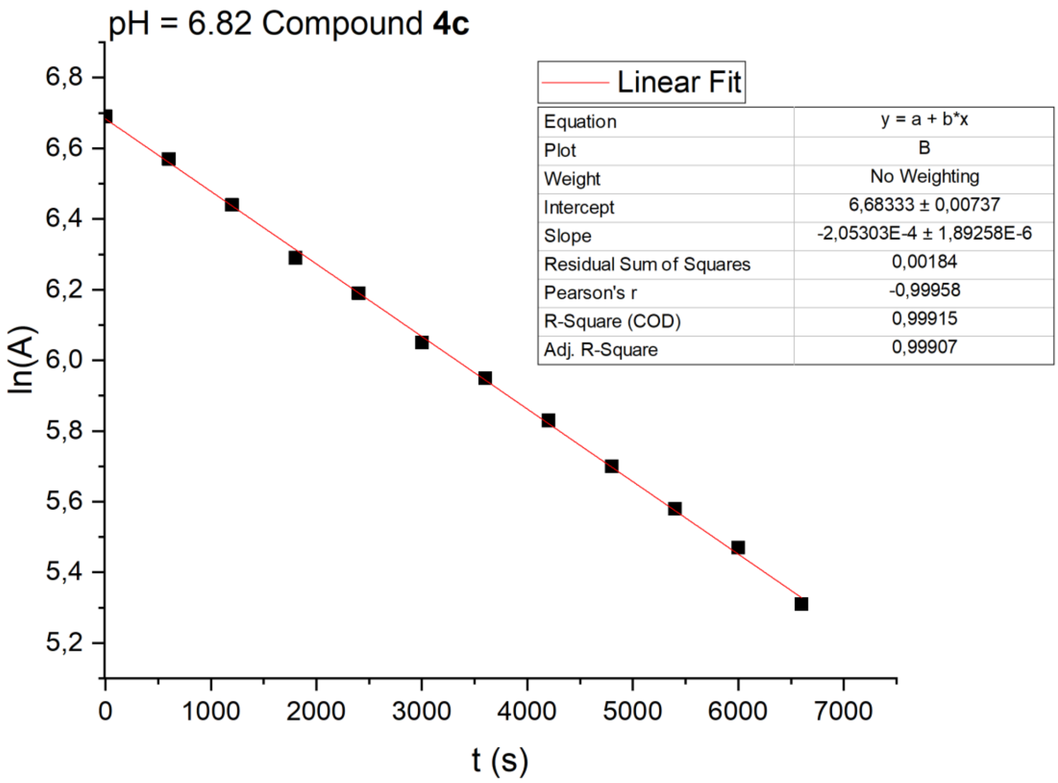
pH = 6.82 Compound 4c
Linear Fit
Equation | y = a + b*x
Plot | B
Weight | No Weighting
Intercept | 6,68333 ± 0,00737
Slope | -2,05303E-4 ± 1,89258E-6
Residual Sum of Squares | 0,00184
Pearson's r | -0,99958
R-Square (COD) | 0,99915
Adj. R-Square | 0,99907
ln(A)
t (s)

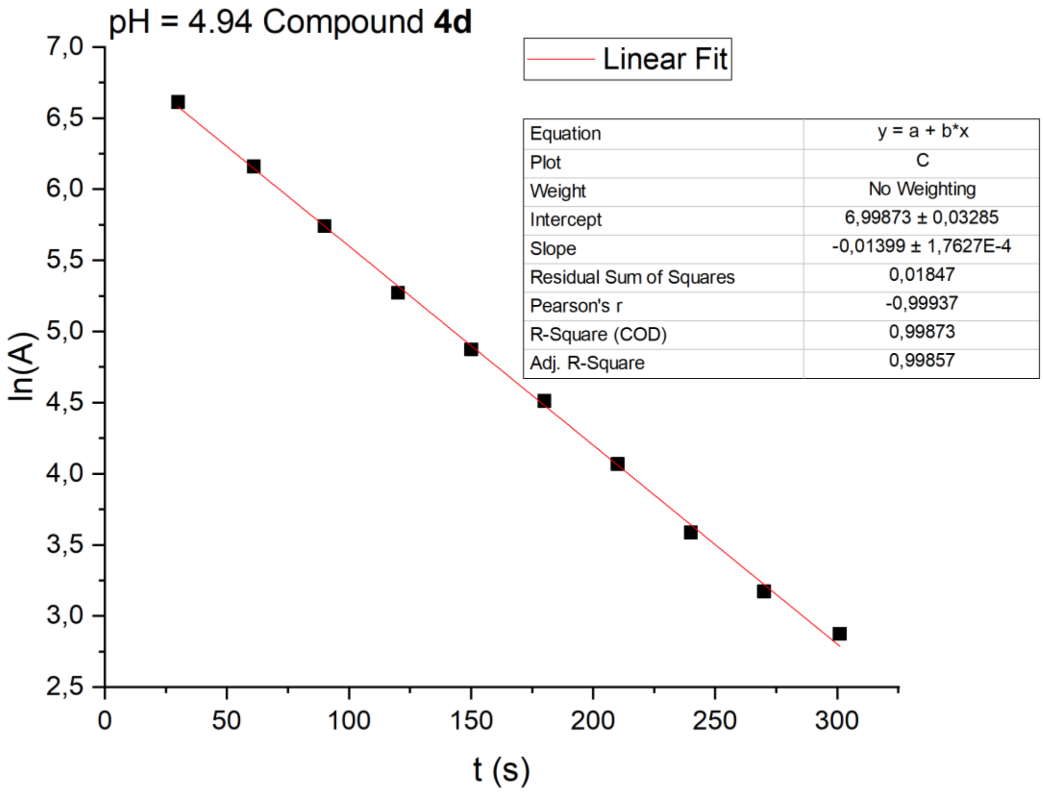
pH = 4.94 Compound 4d
Linear Fit
Equation | y = a + b*x
Plot | C
Weight | No Weighting
Intercept | 6,99873 ± 0,03285
Slope | -0,01399 ± 1,7627E-4
Residual Sum of Squares | 0,01847
Pearson's r | -0,99937
R-Square (COD) | 0,99873
Adj. R-Square | 0,99857
ln(A)
t (s)

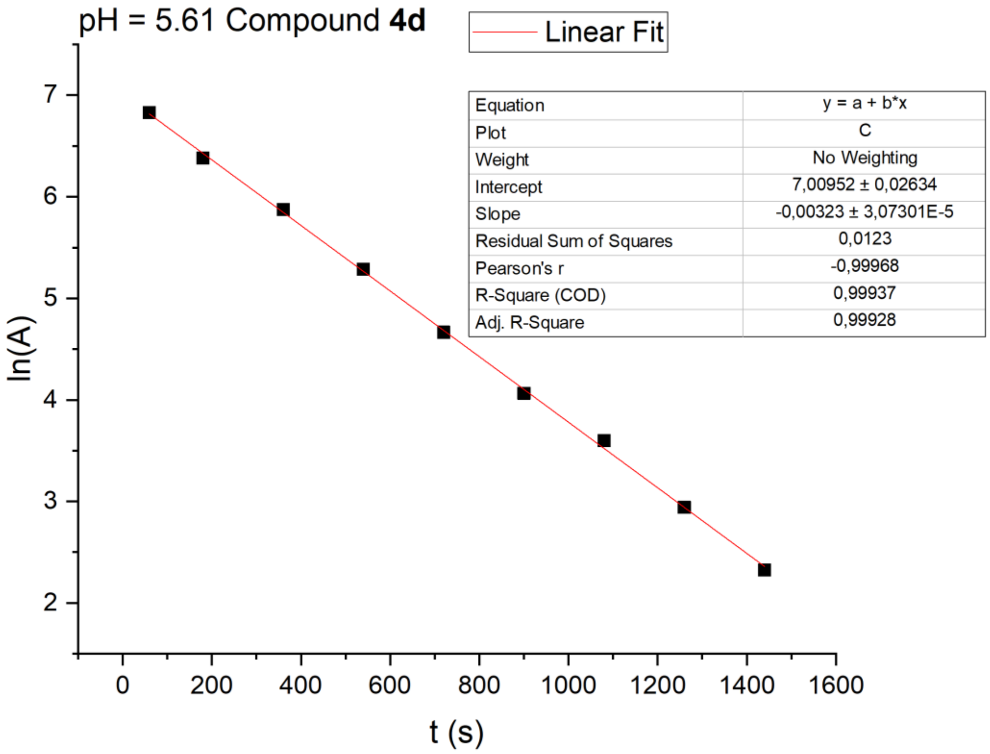
pH = 5.61 Compound **4d**
Linear Fit
Equation | y = a + b*x
Plot | C
Weight | No Weighting
Intercept | 7,00952 ± 0,02634
Slope | -0,00323 ± 3,07301E-5
Residual Sum of Squares | 0,0123
Pearson's r | -0,99968
R-Square (COD) | 0,99937
Adj. R-Square | 0,99928
ln(A)
7
6
5
4
3
2
0
200
400
600
800
1000
1200
1400
1600
t (s)

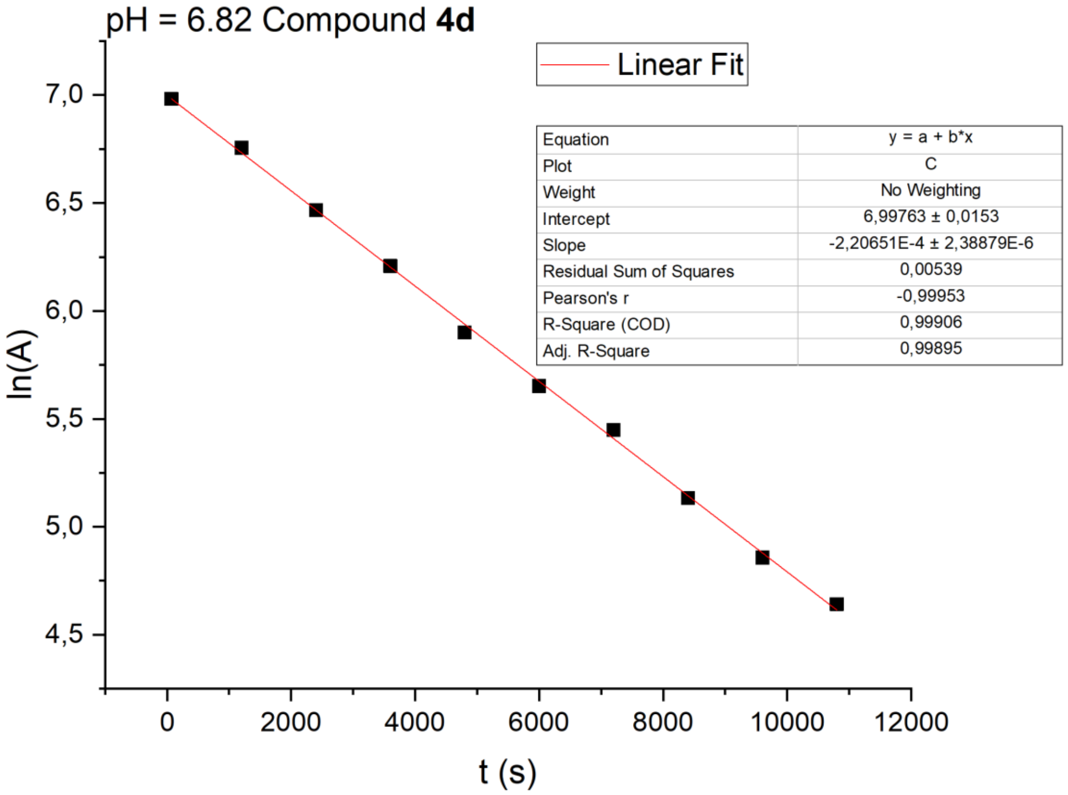
pH = 6.82 Compound **4d**
Linear Fit
Equation | y = a + b*x
Plot | C
Weight | No Weighting
Intercept | 6,99763 ± 0,0153
Slope | -2,20651E-4 ± 2,38879E-6
Residual Sum of Squares | 0,00539
Pearson's r | -0,99953
R-Square (COD) | 0,99906
Adj. R-Square | 0,99895
ln(A)
7,0
6,5
6,0
5,5
5,0
4,5
0
2000
4000
6000
8000
10000
12000
t (s)

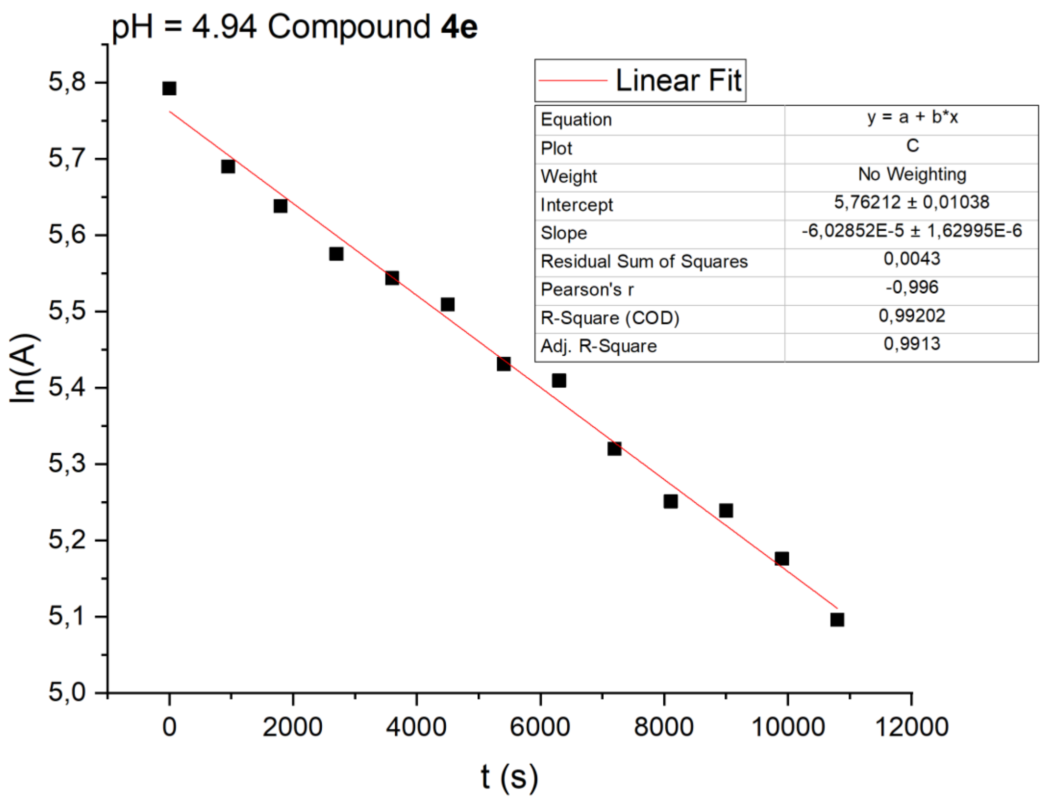
pH = 4.94 Compound 4e
Linear Fit
Equation y = a + b*x
Plot C
Weight No Weighting
Intercept 5,76212 ± 0,01038
Slope -6,02852E-5 ± 1,62995E-6
Residual Sum of Squares 0,0043
Pearson's r -0,996
R-Square (COD) 0,99202
Adj. R-Square 0,9913
ln(A)
t (s)
5,0
5,1
5,2
5,3
5,4
5,5
5,6
5,7
5,8
0
2000
4000
6000
8000
10000
12000

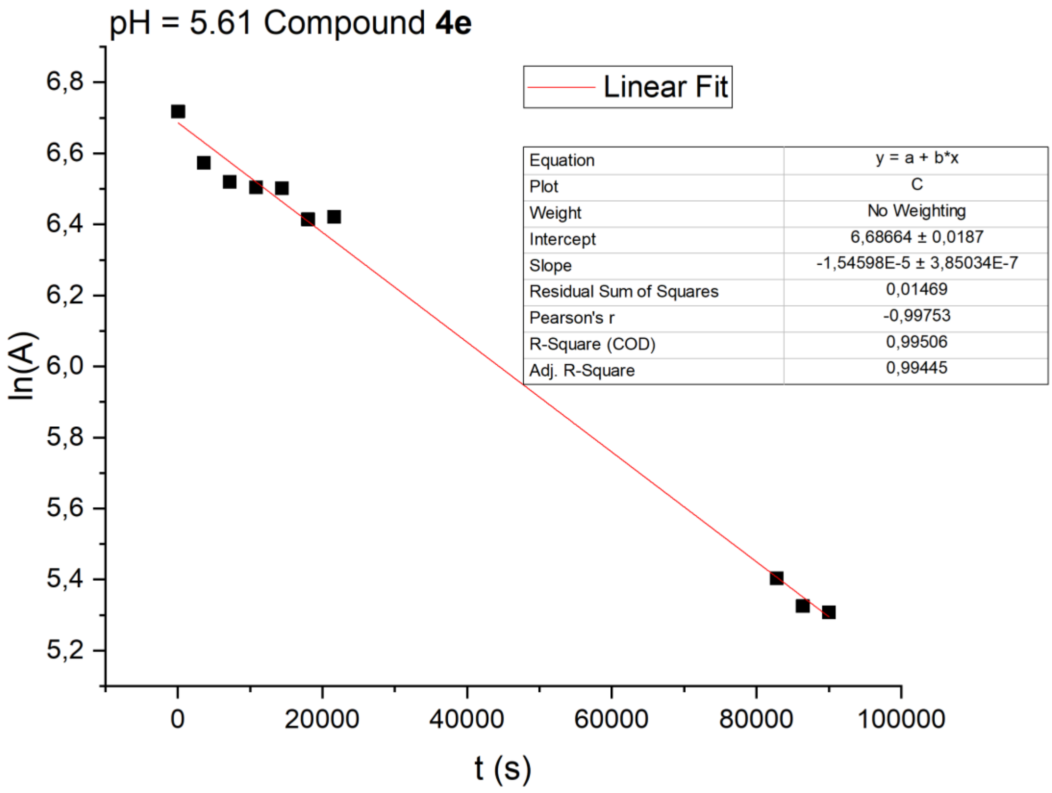
pH = 5.61 Compound 4e
Linear Fit
Equation y = a + b*x
Plot C
Weight No Weighting
Intercept 6,68664 ± 0,0187
Slope -1,54598E-5 ± 3,85034E-7
Residual Sum of Squares 0,01469
Pearson's r -0,99753
R-Square (COD) 0,99506
Adj. R-Square 0,99445
ln(A)
t (s)
5,2
5,4
5,6
5,8
6,0
6,2
6,4
6,6
6,8
0
20000
40000
60000
80000
100000

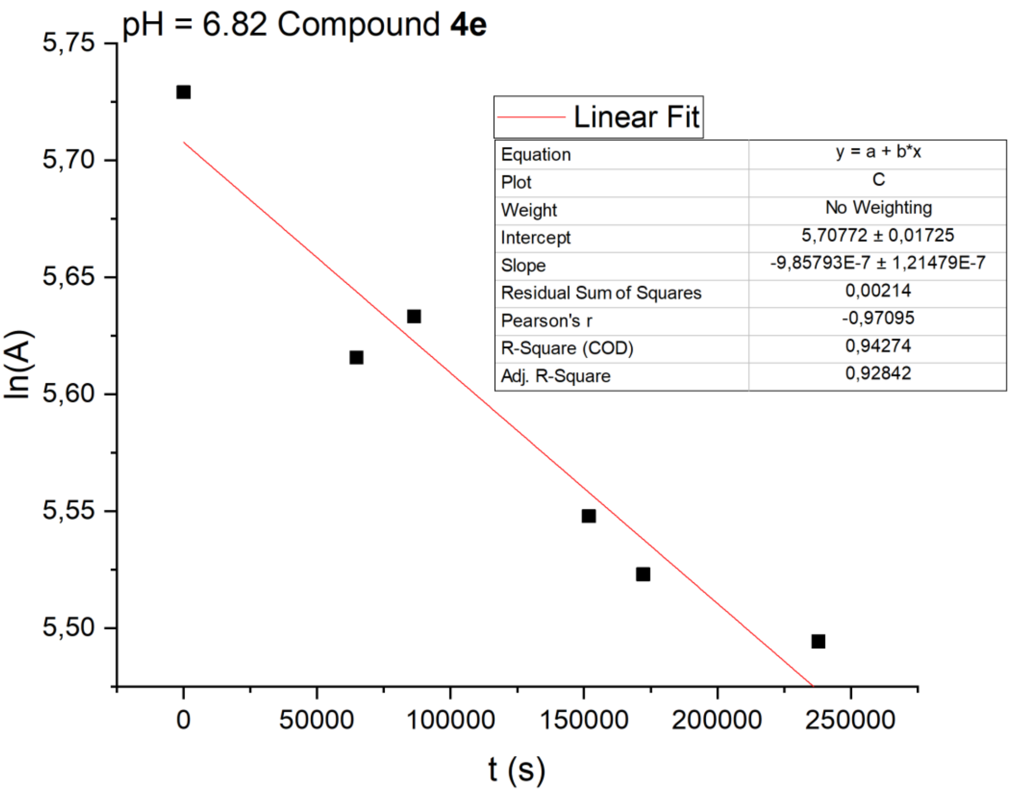
pH = 6.82 Compound 4e
Linear Fit
Equation | y = a + b*x
Plot | C
Weight | No Weighting
Intercept | 5,70772 ± 0,01725
Slope | -9,85793E-7 ± 1,21479E-7
Residual Sum of Squares | 0,00214
Pearson's r | -0,97095
R-Square (COD) | 0,94274
Adj. R-Square | 0,92842
ln(A)
5,75
5,70
5,65
5,60
5,55
5,50
0
50000
100000
150000
200000
250000
t (s)

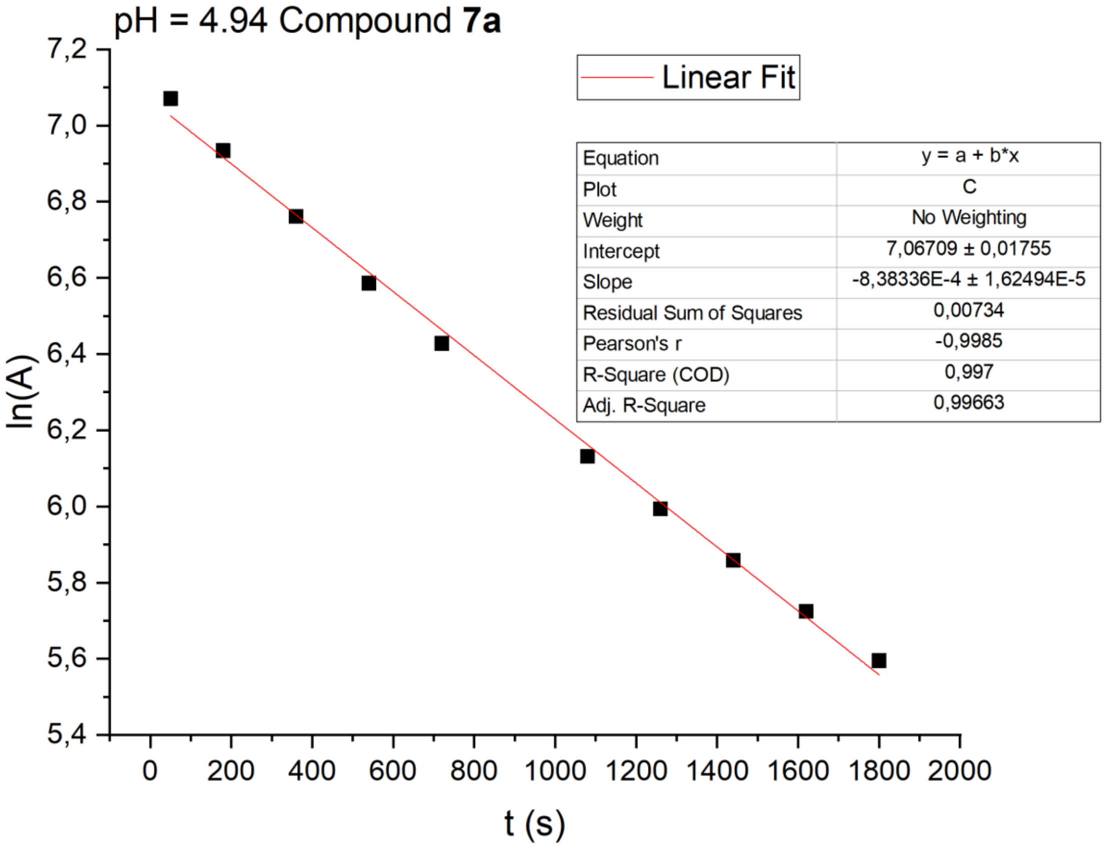
pH = 4.94 Compound 7a
Linear Fit
Equation | y = a + b*x
Plot | C
Weight | No Weighting
Intercept | 7,06709 ± 0,01755
Slope | -8,38336E-4 ± 1,62494E-5
Residual Sum of Squares | 0,00734
Pearson's r | -0,9985
R-Square (COD) | 0,997
Adj. R-Square | 0,99663
ln(A)
7,2
7,0
6,8
6,6
6,4
6,2
6,0
5,8
5,6
5,4
0
200
400
600
800
1000
1200
1400
1600
1800
2000
t (s)

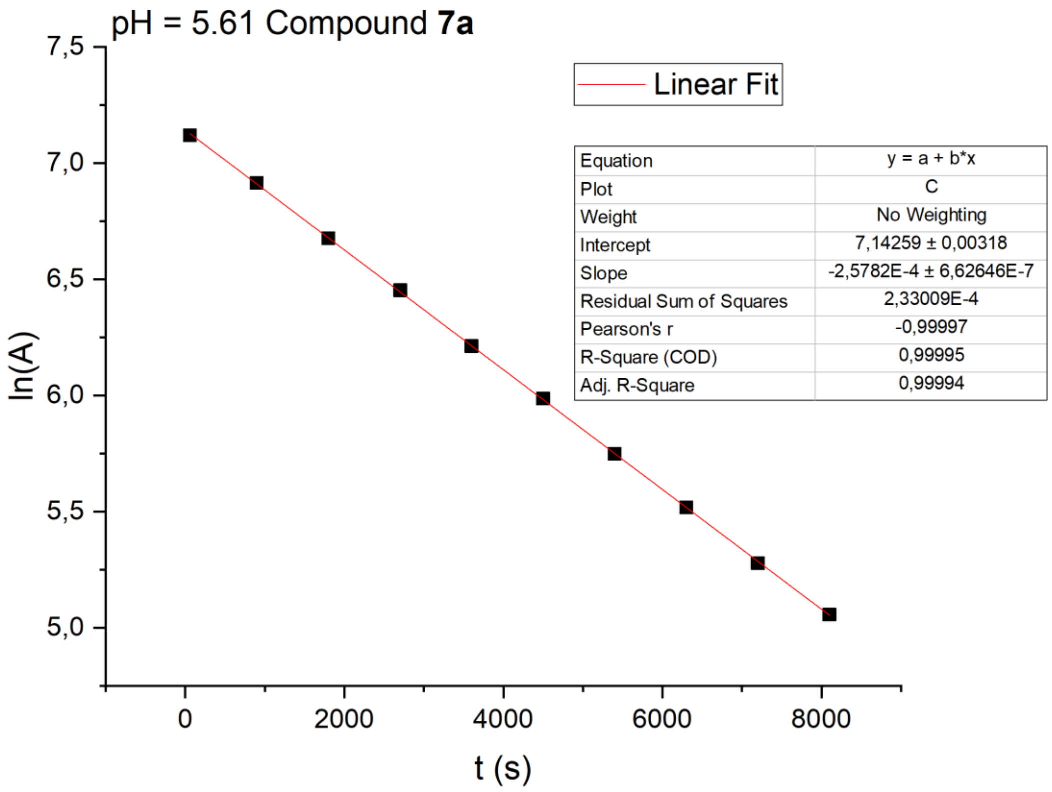
pH = 5.61 Compound 7a
Linear Fit
Equation | y = a + b*x
Plot | C
Weight | No Weighting
Intercept | 7,14259 ± 0,00318
Slope | -2,5782E-4 ± 6,62646E-7
Residual Sum of Squares | 2,33009E-4
Pearson's r | -0,99997
R-Square (COD) | 0,99995
Adj. R-Square | 0,99994
ln(A)
t (s)

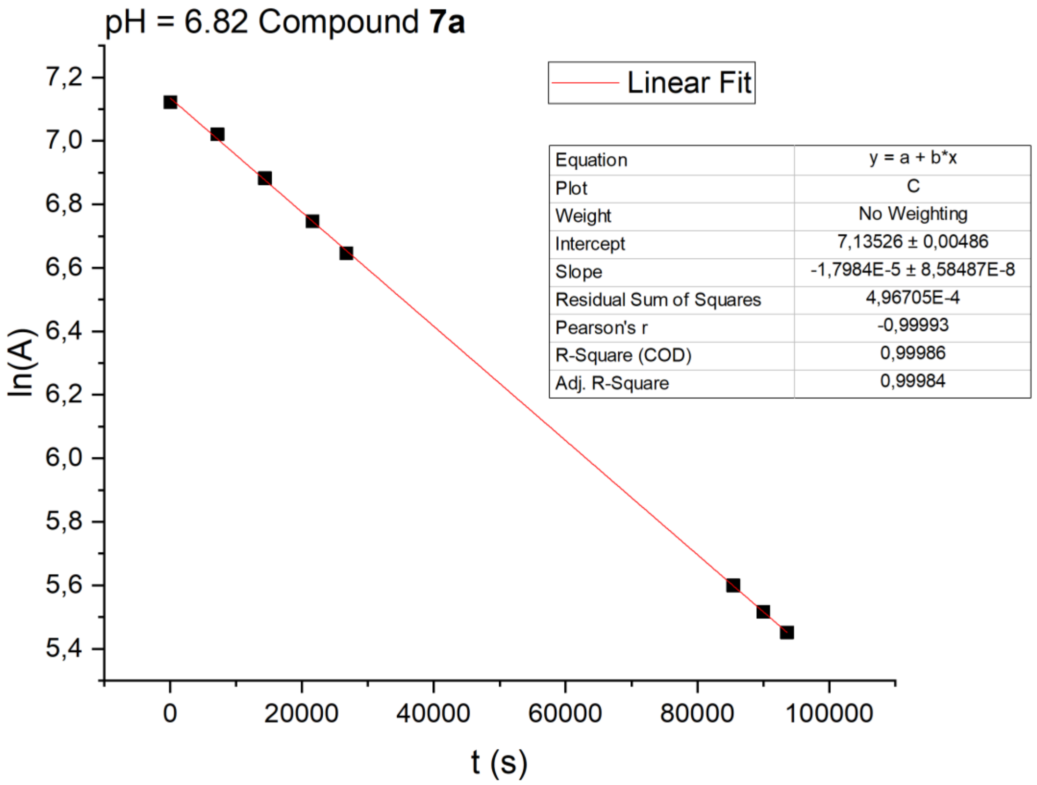
pH = 6.82 Compound 7a
Linear Fit
Equation | y = a + b*x
Plot | C
Weight | No Weighting
Intercept | 7,13526 ± 0,00486
Slope | -1,7984E-5 ± 8,58487E-8
Residual Sum of Squares | 4,96705E-4
Pearson's r | -0,99993
R-Square (COD) | 0,99986
Adj. R-Square | 0,99984
ln(A)
t (s)

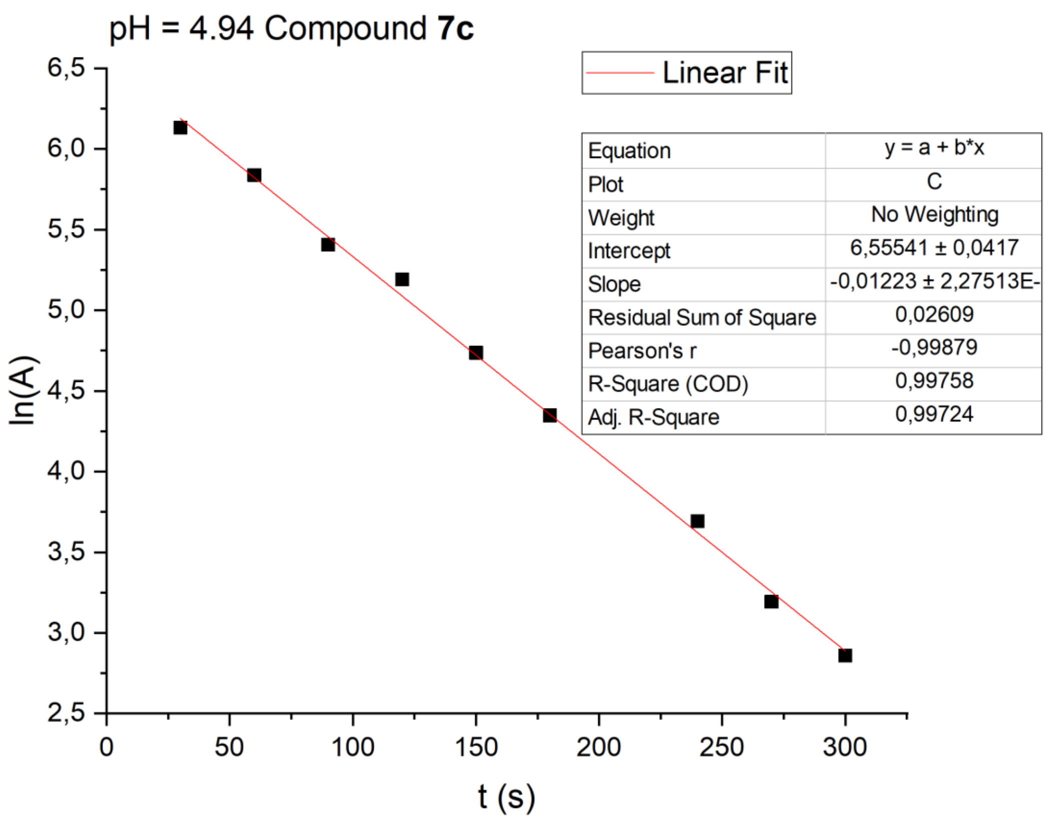
pH = 4.94 Compound 7c
Linear Fit
Equation y = a + b*x
Plot C
Weight No Weighting
Intercept 6,55541 ± 0,0417
Slope -0,01223 ± 2,27513E-
Residual Sum of Square 0,02609
Pearson's r -0,99879
R-Square (COD) 0,99758
Adj. R-Square 0,99724
ln(A)
t (s)

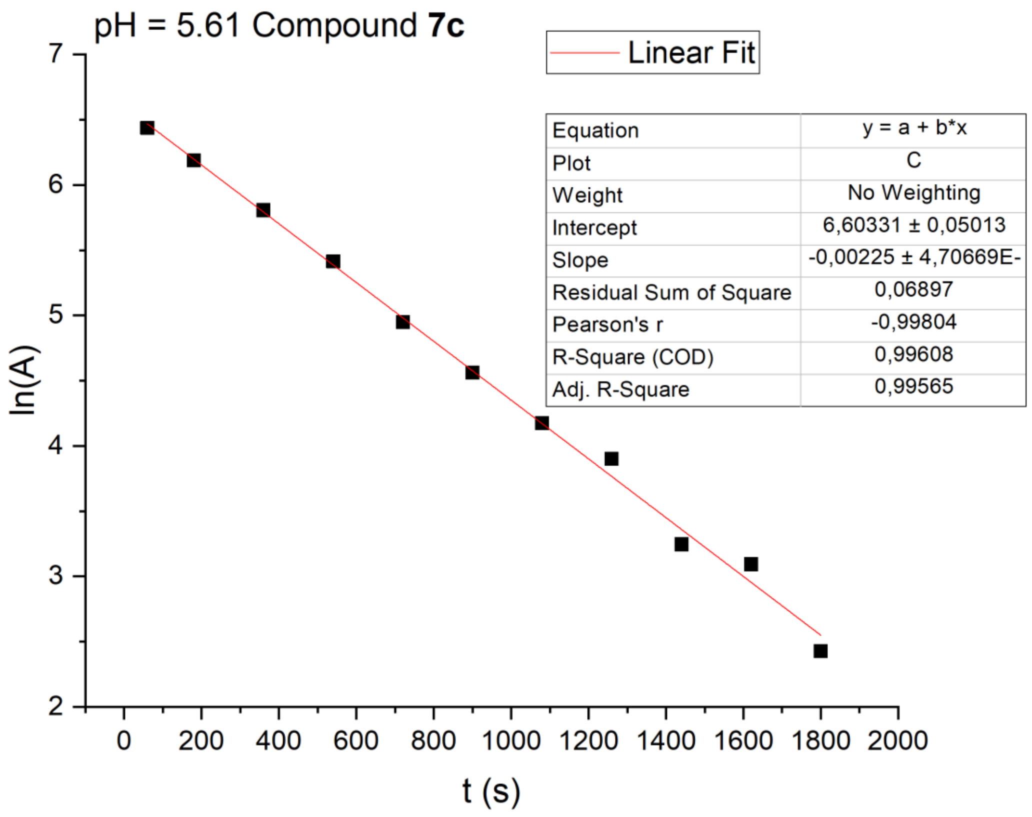
pH = 5.61 Compound 7c
Linear Fit
Equation y = a + b*x
Plot C
Weight No Weighting
Intercept 6,60331 ± 0,05013
Slope -0,00225 ± 4,70669E-
Residual Sum of Square 0,06897
Pearson's r -0,99804
R-Square (COD) 0,99608
Adj. R-Square 0,99565
ln(A)
t (s)

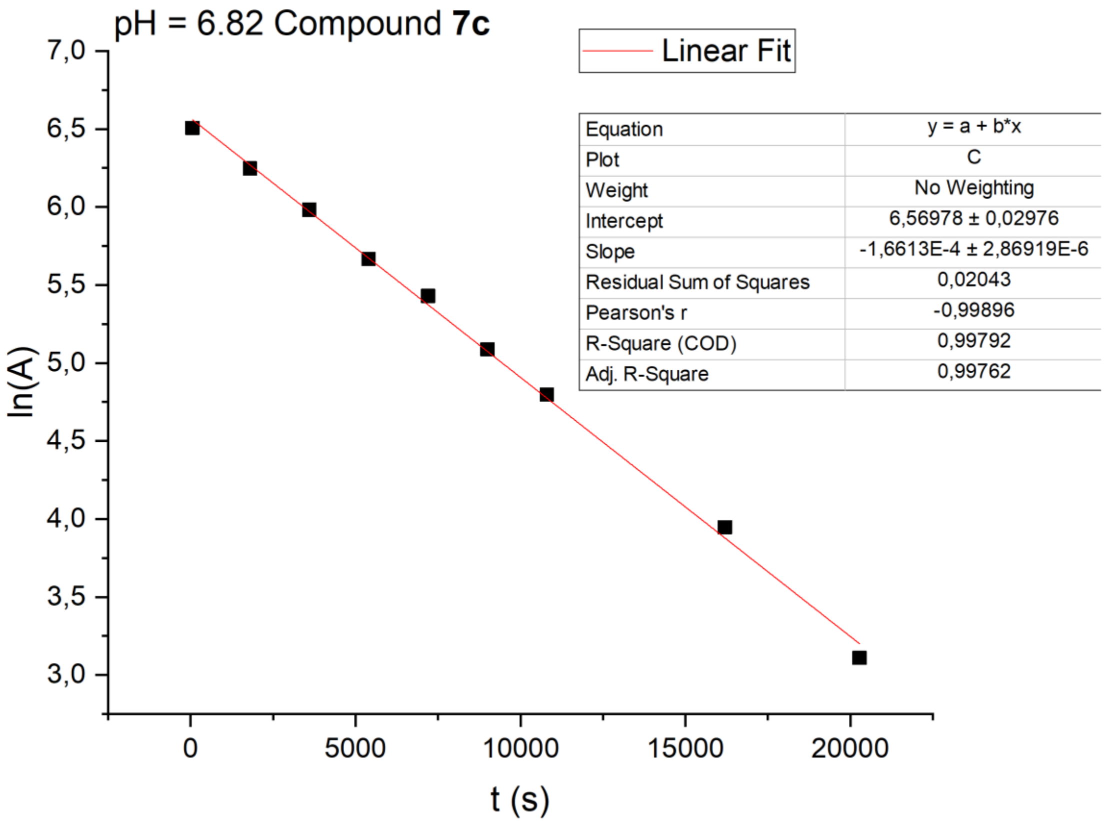
pH = 6.82 Compound 7c
Linear Fit
Equation | y = a + b*x
Plot | C
Weight | No Weighting
Intercept | 6,56978 ± 0,02976
Slope | -1,6613E-4 ± 2,86919E-6
Residual Sum of Squares | 0,02043
Pearson's r | -0,99896
R-Square (COD) | 0,99792
Adj. R-Square | 0,99762
ln(A)
t (s)

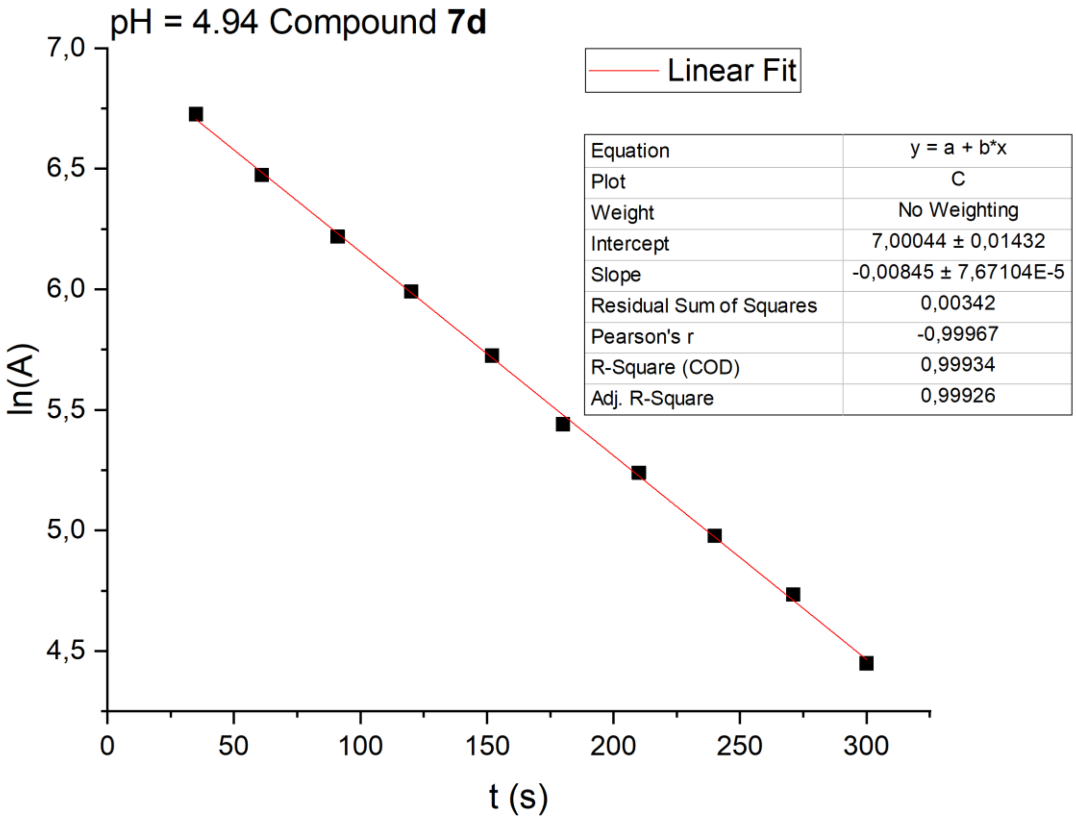
pH = 4.94 Compound 7d
Linear Fit
Equation | y = a + b*x
Plot | C
Weight | No Weighting
Intercept | 7,00044 ± 0,01432
Slope | -0,00845 ± 7,67104E-5
Residual Sum of Squares | 0,00342
Pearson's r | -0,99967
R-Square (COD) | 0,99934
Adj. R-Square | 0,99926
ln(A)
t (s)

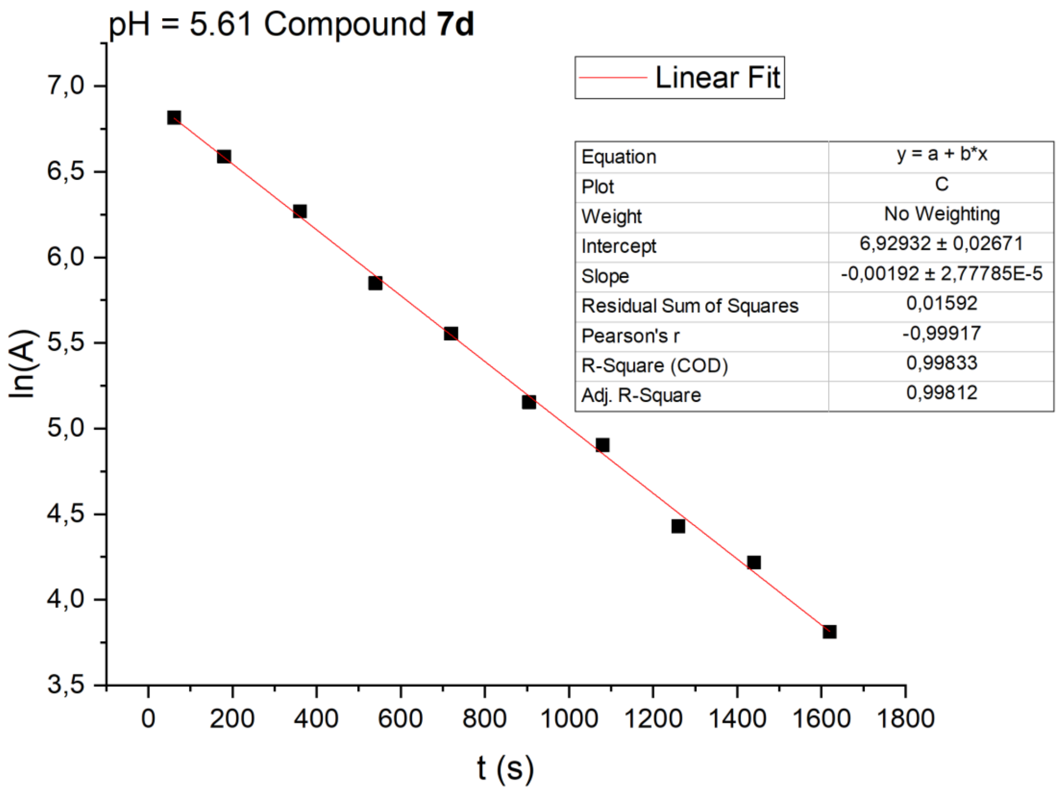
pH = 5.61 Compound **7d**
Linear Fit
Equation | y = a + b*x
Plot | C
Weight | No Weighting
Intercept | 6,92932 ± 0,02671
Slope | -0,00192 ± 2,77785E-5
Residual Sum of Squares | 0,01592
Pearson's r | -0,99917
R-Square (COD) | 0,99833
Adj. R-Square | 0,99812
ln(A)
t (s)

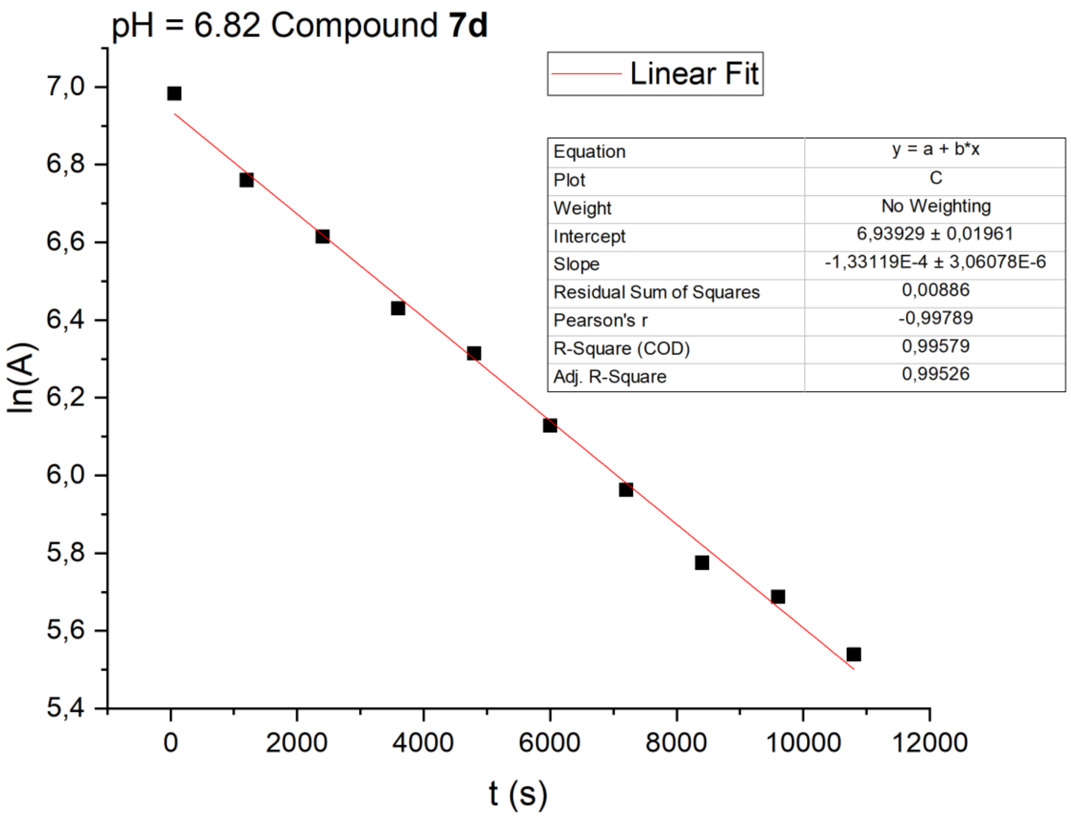
pH = 6.82 Compound **7d**
Linear Fit
Equation | y = a + b*x
Plot | C
Weight | No Weighting
Intercept | 6,93929 ± 0,01961
Slope | -1,33119E-4 ± 3,06078E-6
Residual Sum of Squares | 0,00886
Pearson's r | -0,99789
R-Square (COD) | 0,99579
Adj. R-Square | 0,99526
ln(A)
t (s)

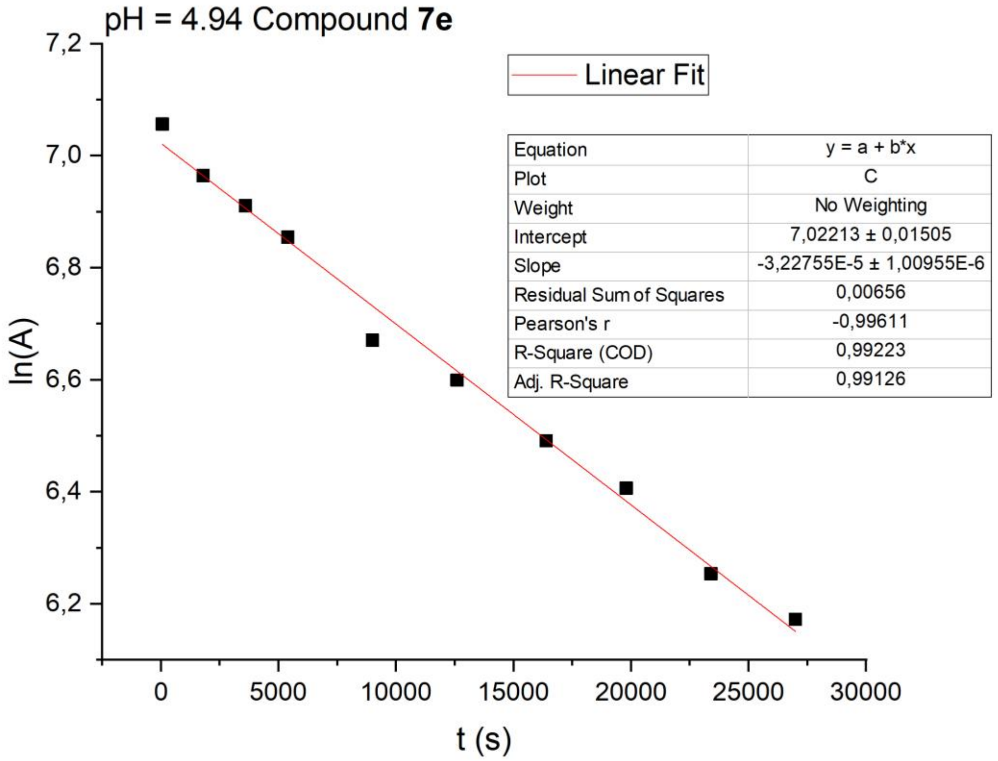
pH = 4.94 Compound 7e
Linear Fit
Equation | y = a + b*x
Plot | C
Weight | No Weighting
Intercept | 7,02213 ± 0,01505
Slope | -3,22755E-5 ± 1,00955E-6
Residual Sum of Squares | 0,00656
Pearson's r | -0,99611
R-Square (COD) | 0,99223
Adj. R-Square | 0,99126
ln(A)
t (s)

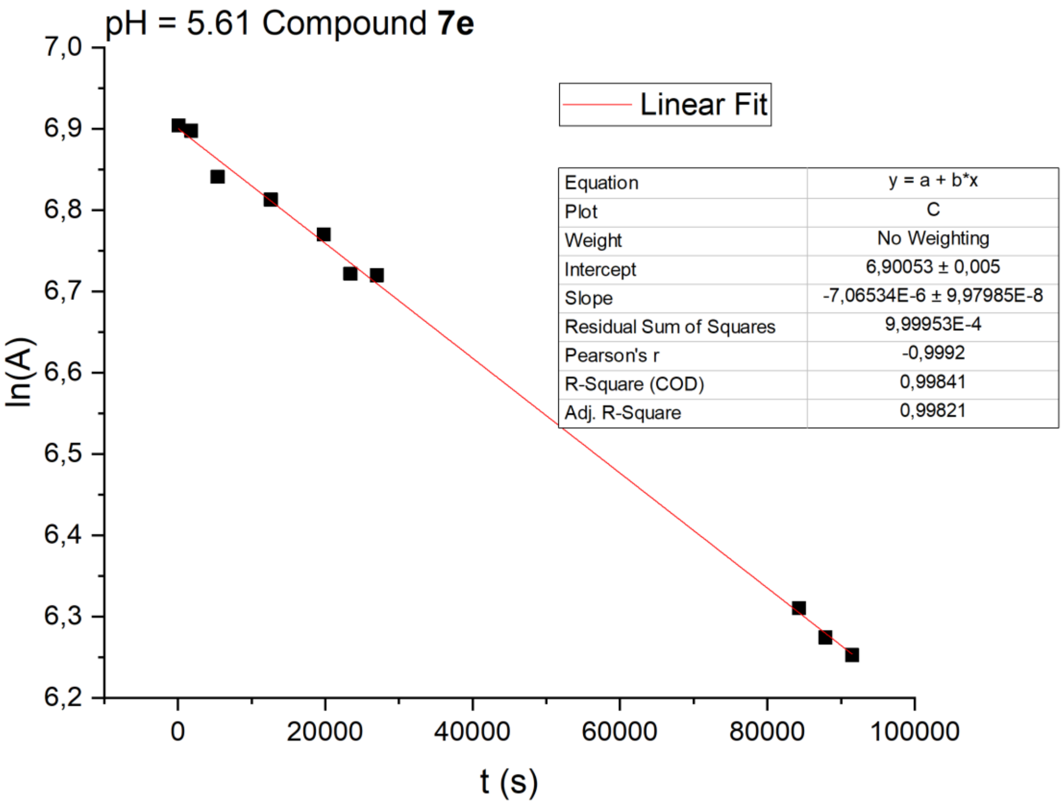
pH = 5.61 Compound 7e
Linear Fit
Equation | y = a + b*x
Plot | C
Weight | No Weighting
Intercept | 6,90053 ± 0,005
Slope | -7,06534E-6 ± 9,97985E-8
Residual Sum of Squares | 9,99953E-4
Pearson's r | -0,9992
R-Square (COD) | 0,99841
Adj. R-Square | 0,99821
ln(A)
t (s)

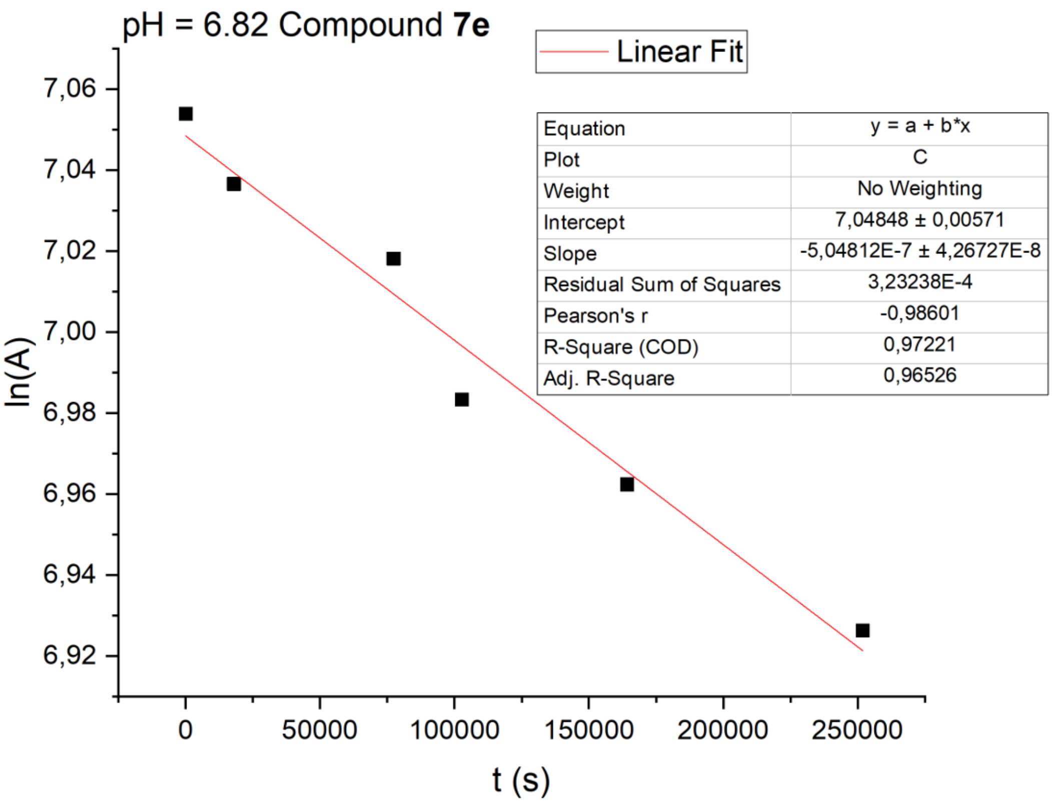
pH = 6.82 Compound 7e
Linear Fit
Equation y = a + b*x
Plot C
Weight No Weighting
Intercept 7,04848 ± 0,00571
Slope -5,04812E-7 ± 4,26727E-8
Residual Sum of Squares 3,23238E-4
Pearson's r -0,98601
R-Square (COD) 0,97221
Adj. R-Square 0,96526
ln(A)
7,06
7,04
7,02
7,00
6,98
6,96
6,94
6,92
0
50000
100000
150000
200000
250000
t (s)

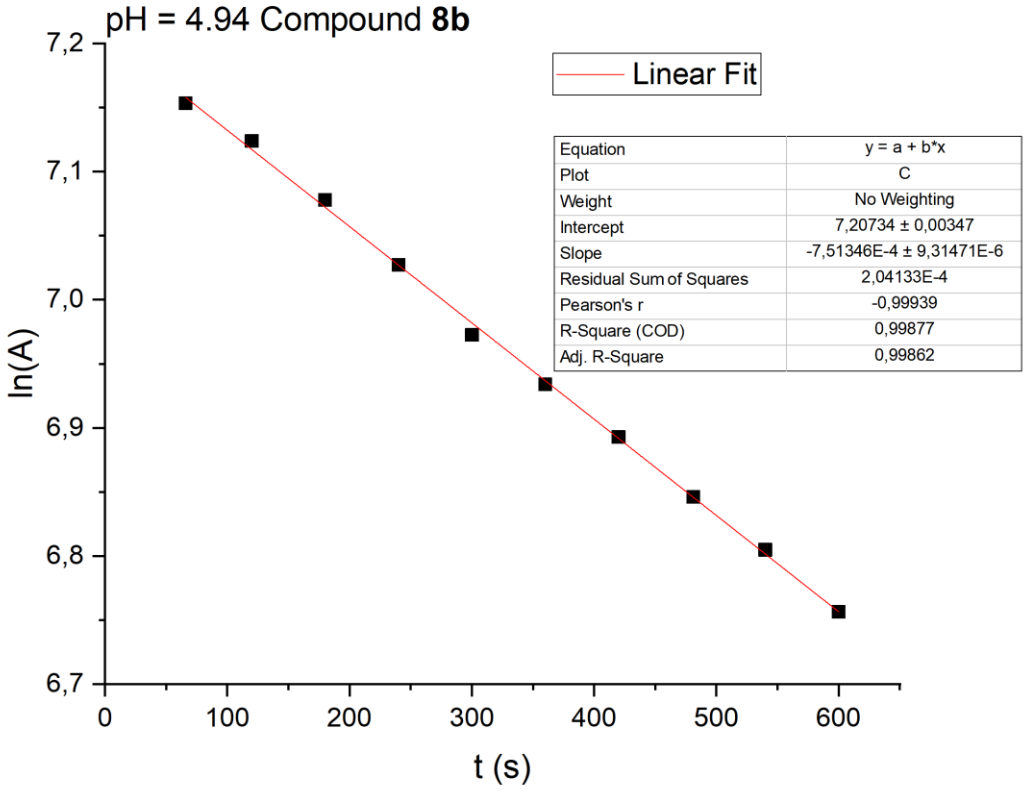
pH = 4.94 Compound 8b
Linear Fit
Equation y = a + b*x
Plot C
Weight No Weighting
Intercept 7,20734 ± 0,00347
Slope -7,51346E-4 ± 9,31471E-6
Residual Sum of Squares 2,04133E-4
Pearson's r -0,99939
R-Square (COD) 0,99877
Adj. R-Square 0,99862
ln(A)
7,2
7,1
7,0
6,9
6,8
6,7
0
100
200
300
400
500
600
t (s)

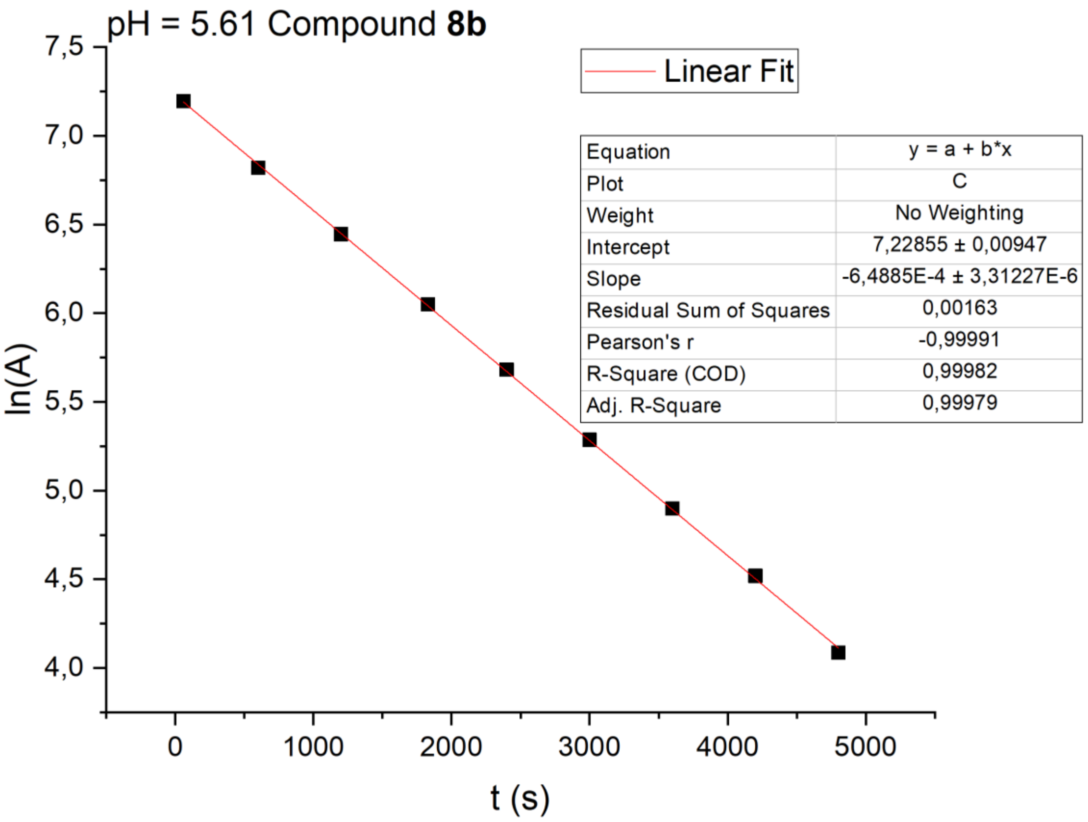
pH = 5.61 Compound **8b**
Linear Fit
Equation y = a + b*x
Plot C
Weight No Weighting
Intercept 7,22855 ± 0,00947
Slope -6,4885E-4 ± 3,31227E-6
Residual Sum of Squares 0,00163
Pearson's r -0,99991
R-Square (COD) 0,99982
Adj. R-Square 0,99979
ln(A)
t (s)

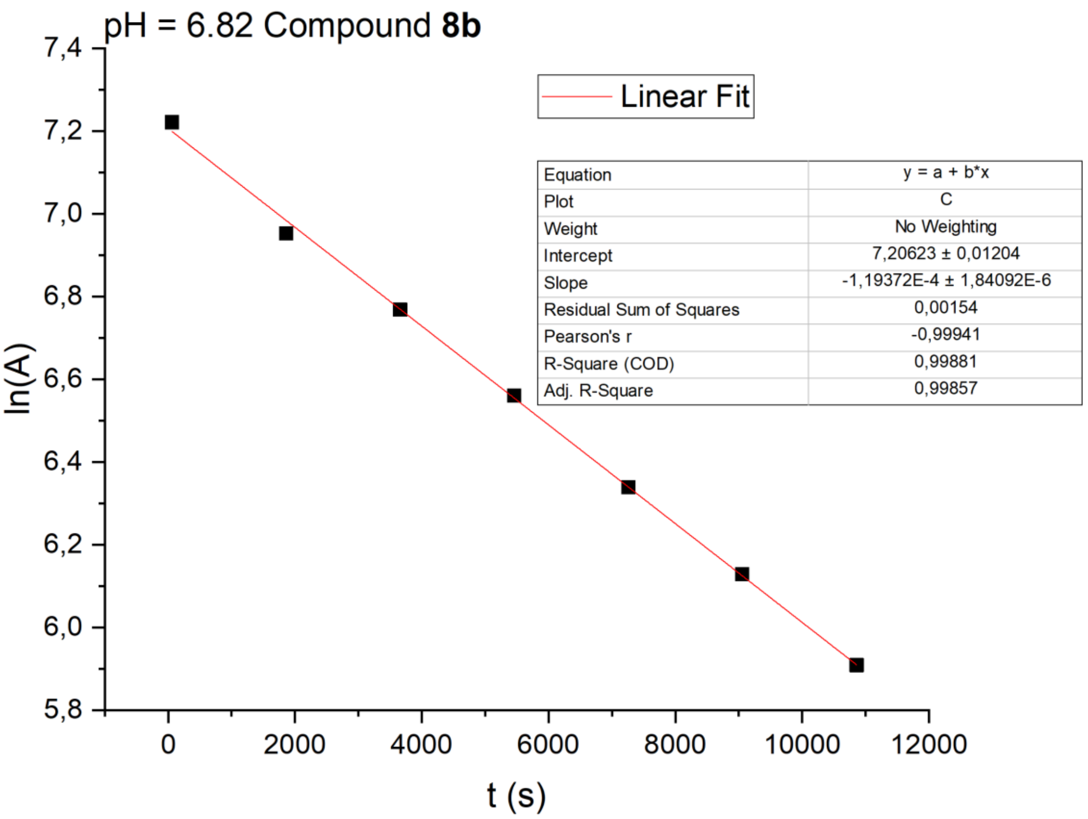
pH = 6.82 Compound **8b**
Linear Fit
Equation y = a + b*x
Plot C
Weight No Weighting
Intercept 7,20623 ± 0,01204
Slope -1,19372E-4 ± 1,84092E-6
Residual Sum of Squares 0,00154
Pearson's r -0,99941
R-Square (COD) 0,99881
Adj. R-Square 0,99857
ln(A)
t (s)
