# Supplementary material for: Tuning the stability of alkoxyisopropyl protection groups
Source: Beilstein J Org Chem. 2019 Mar 21;15:746–51. doi: 10.3762/bjoc.15.70 (PMC6444389; doi:10.3762/bjoc.15.70)

BEILSTEIN JOURNAL OF ORGANIC CHEMISTRY

Supporting Information

for

Tuning the stability of alkoxyisopropyl protection groups

Zehong Liang, Henna Koivikko, Mikko Oivanen and Petri Heinonen

Beilstein J. Org. Chem. **2019,** *15,* 746–751. doi:10.3762/bjoc.15.70

Copies of ^{1}H NMR and ^{13}C NMR spectra

License and Terms: This is a supporting information file under the terms of the Creative Commons Attribution License (http://creativecommons.org/licenses/by/4.0). Please note that the reuse, redistribution and reproduction in particular requires that the authors and source are credited.
The license is subject to the *Beilstein Journal of Organic Chemistry* terms and conditions: (https://www.beilstein-journals.org/bjoc)

^{1}H and ^{13}C NMR spectra

3-(2,2,2-Trifluoroethoxy)but-2-enoic acid (**9**).

^{1}H-NMR (500 MHz, DMSO-d_6)

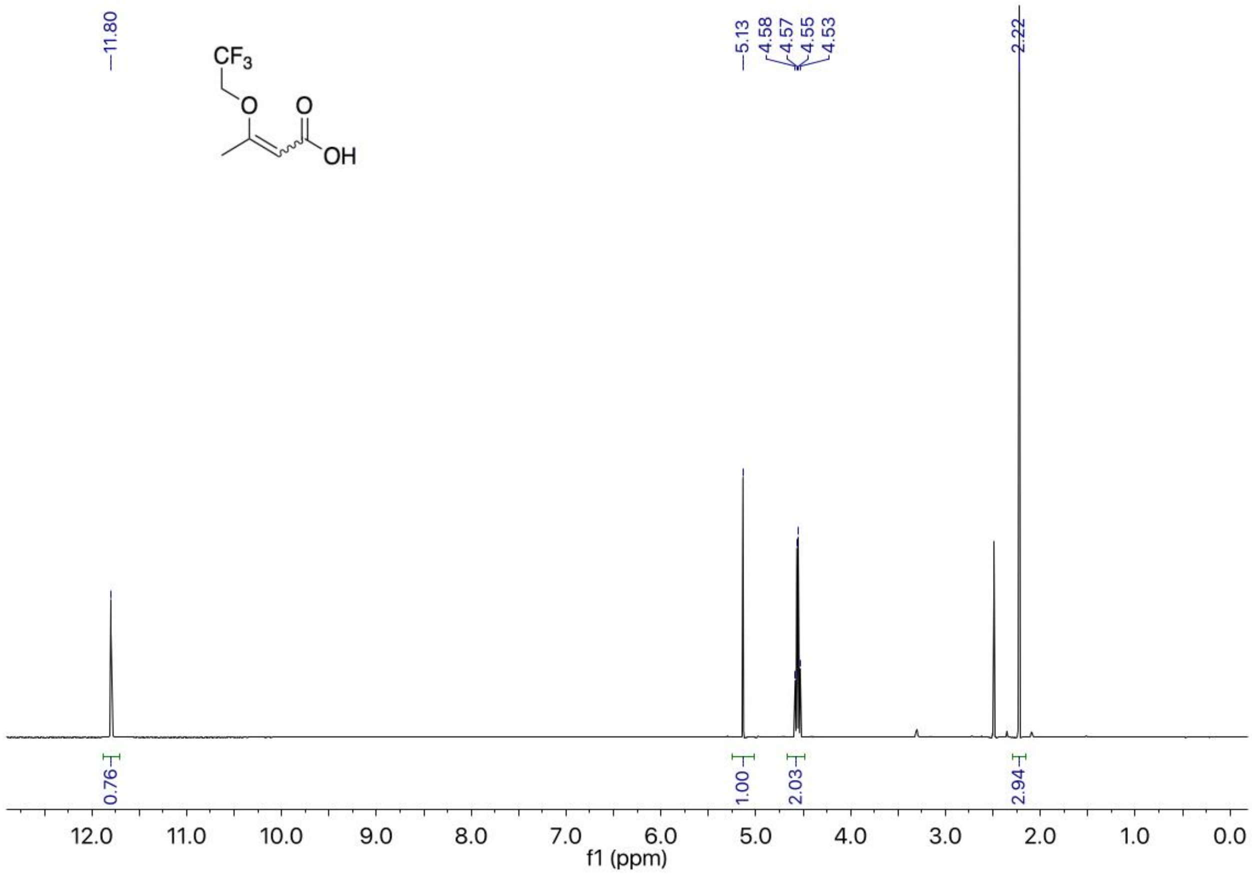

^{13}C NMR (75 MHz, DMSO-d_6)

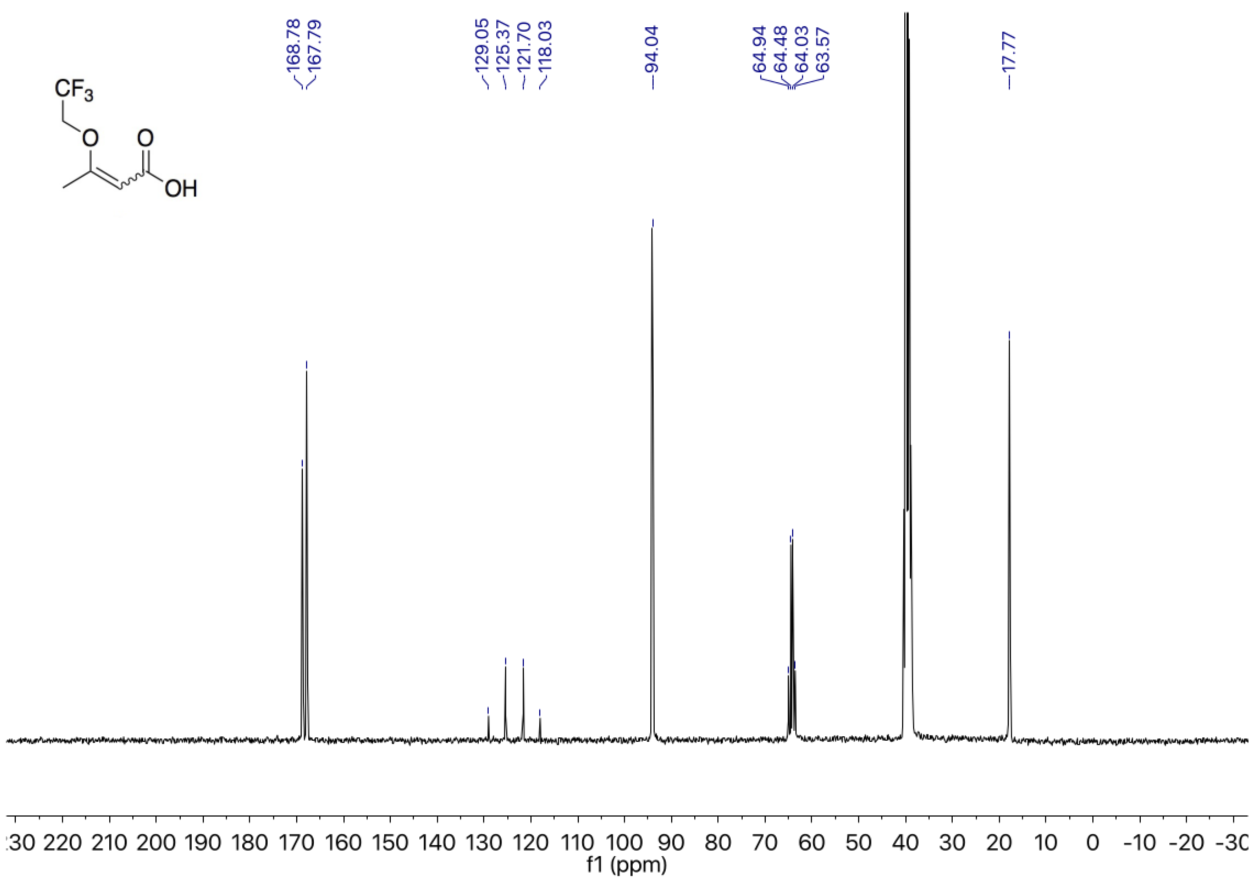

2-(2,2,2-Trifluoroethoxy)prop-1-ene (**1e**).

^{1}H-NMR (500 MHz, DMSO-d_6)

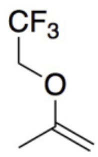

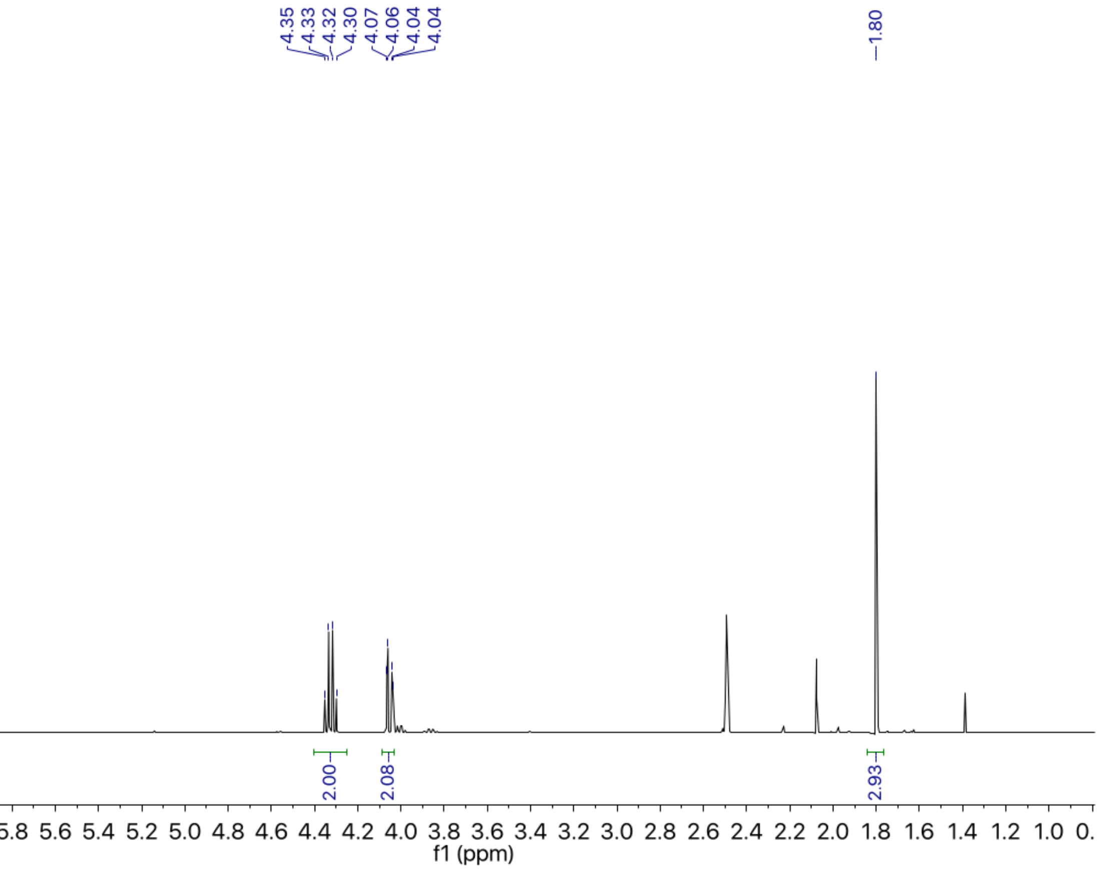

^{13}C-NMR (125 MHz, DMSO-d_6)

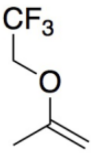

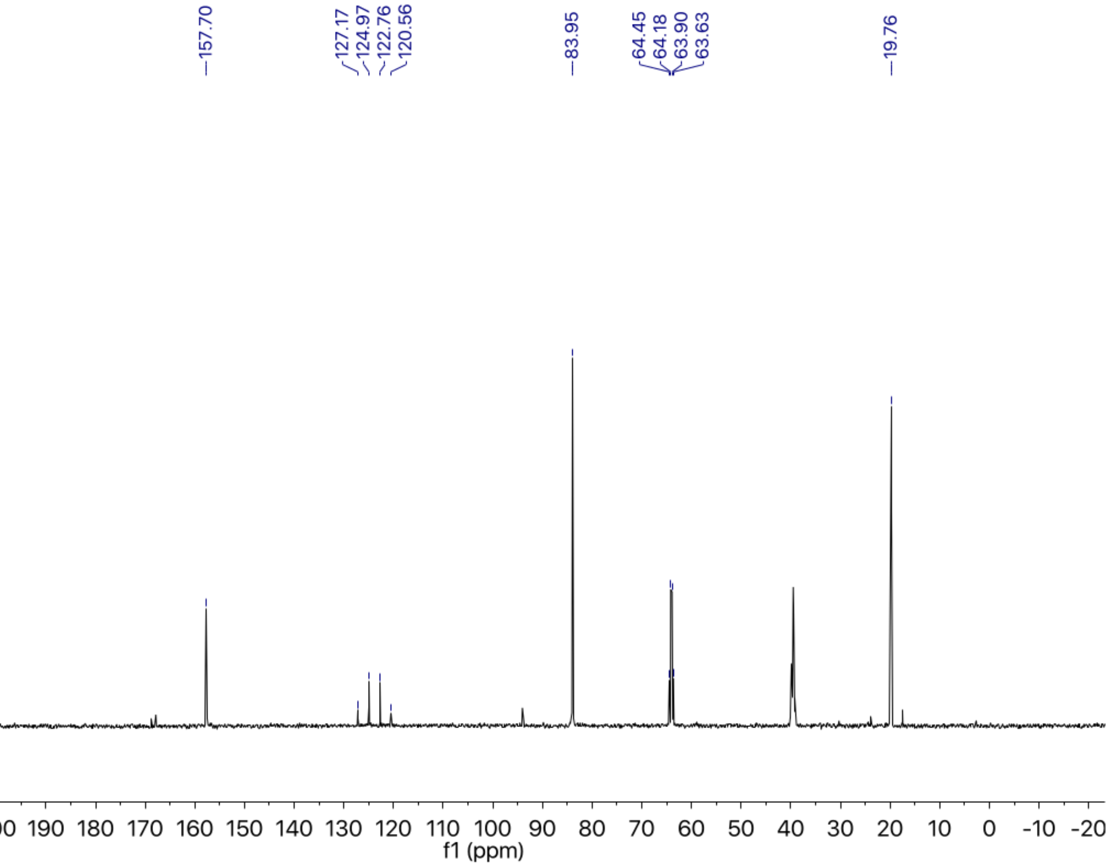

3'-O-Benzoyl-5'-O-methoxypropan-2-yl-2'-deoxythymidine (**3a**).

^{1}H-NMR (300 MHz, $CDCl_3$):

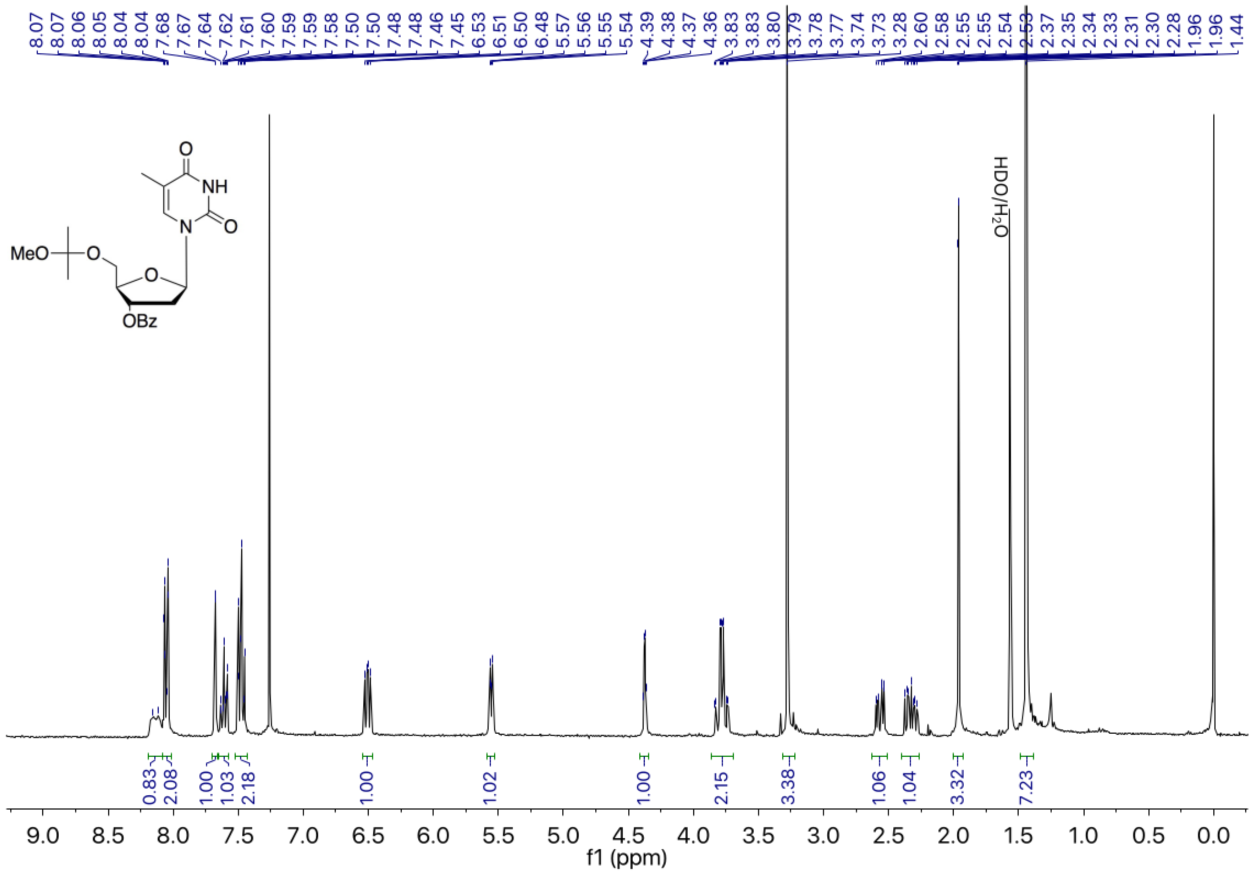

^{13}C-NMR (125 MHz, $CDCl_3$)

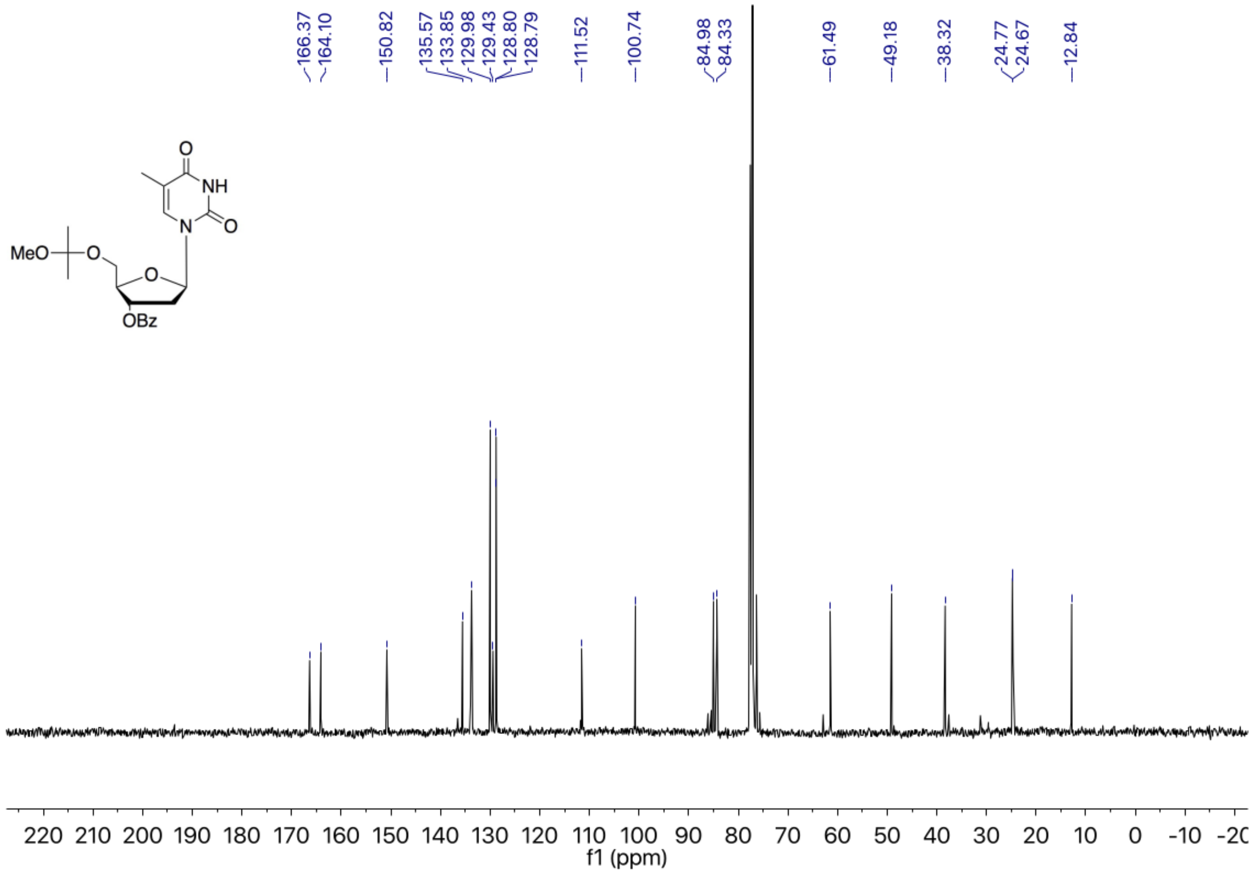

3'-O-Benzoyl-5'-O-benzyloxypropan-2-yl-2'-deoxythymidine (**3b**).

^{1}H-NMR (500 MHz, DMSO-d_6):

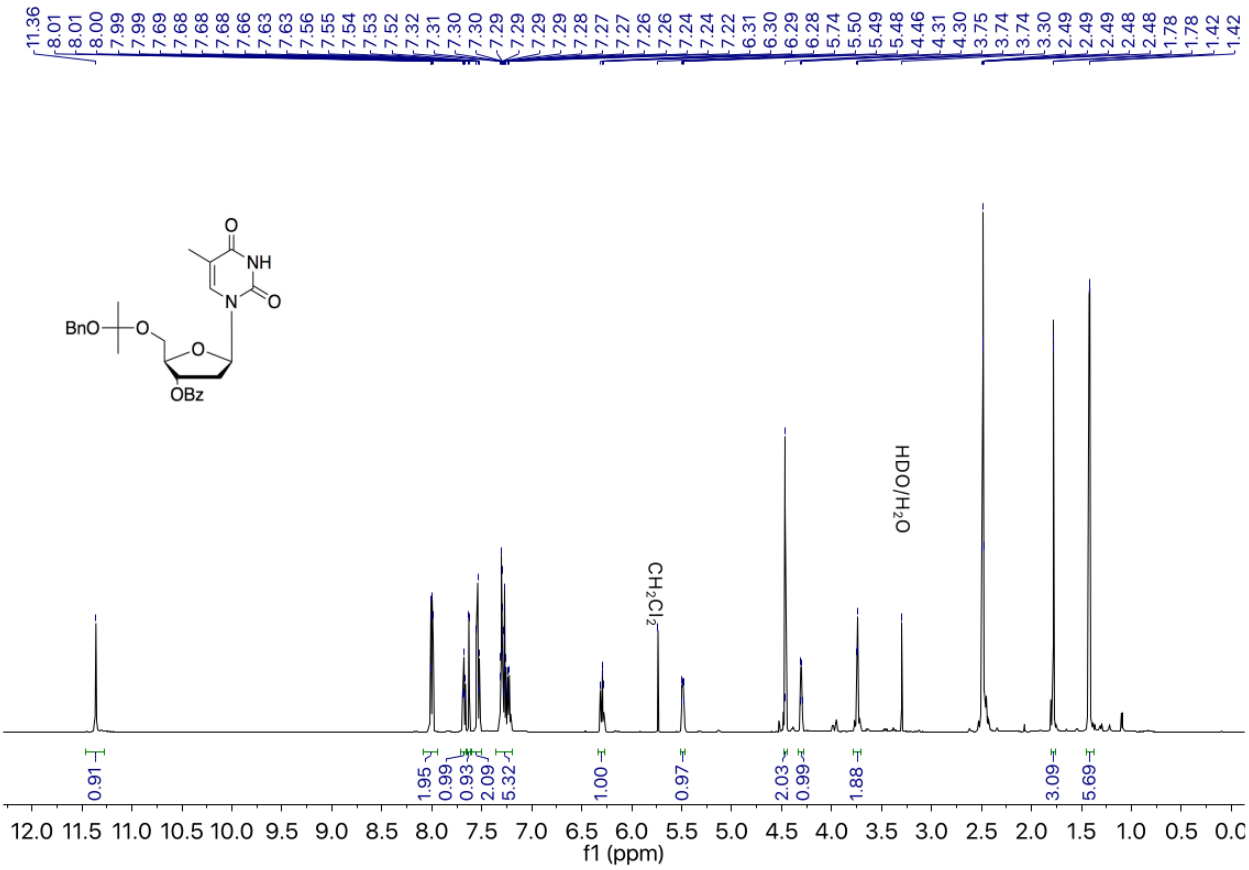

^{13}C-NMR (125 MHz, DMSO-d6)

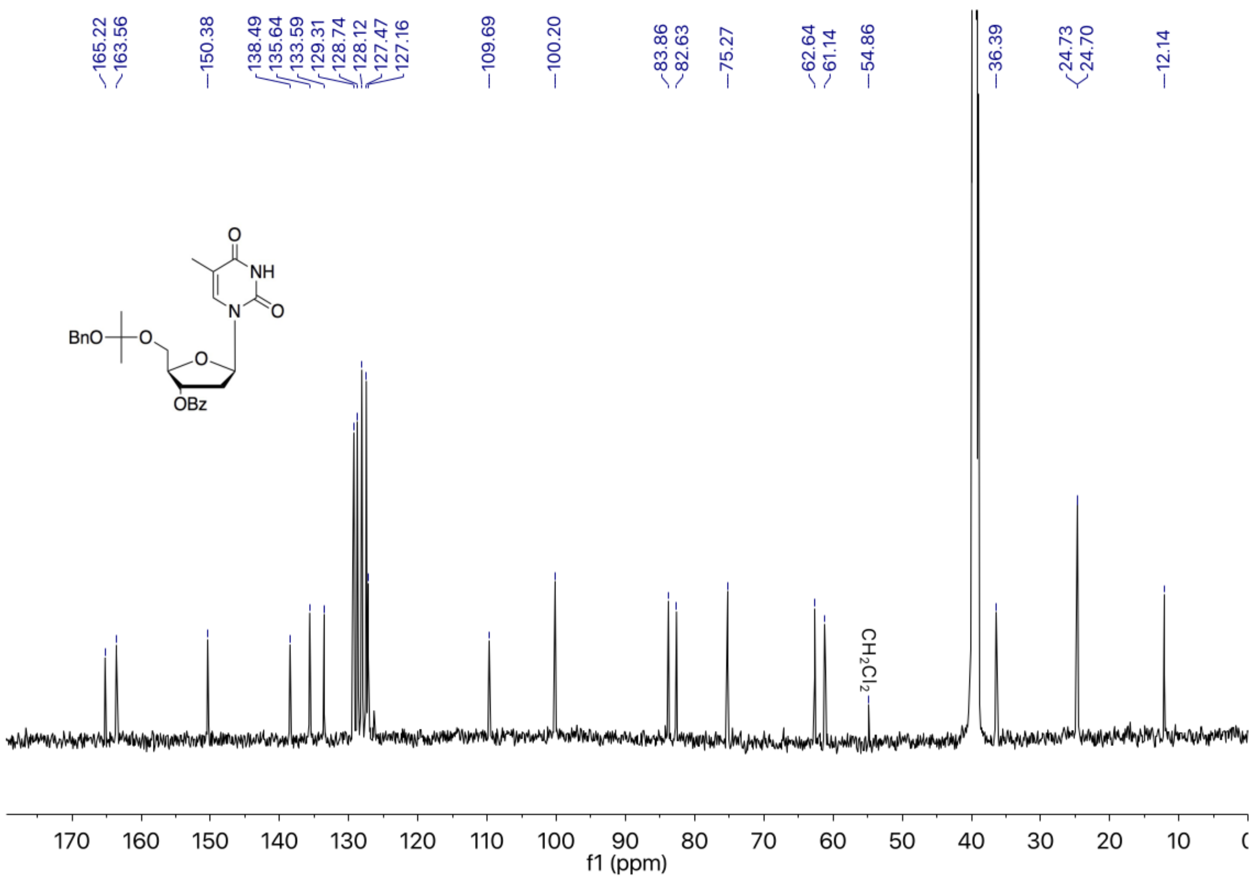

3’-O-Benzoyl-5’-O-cyclohexyloxypropan-2-yl-2’-deoxythymidine (**3c**).

^{1}H-NMR (500 MHz, Acetone-d_6)

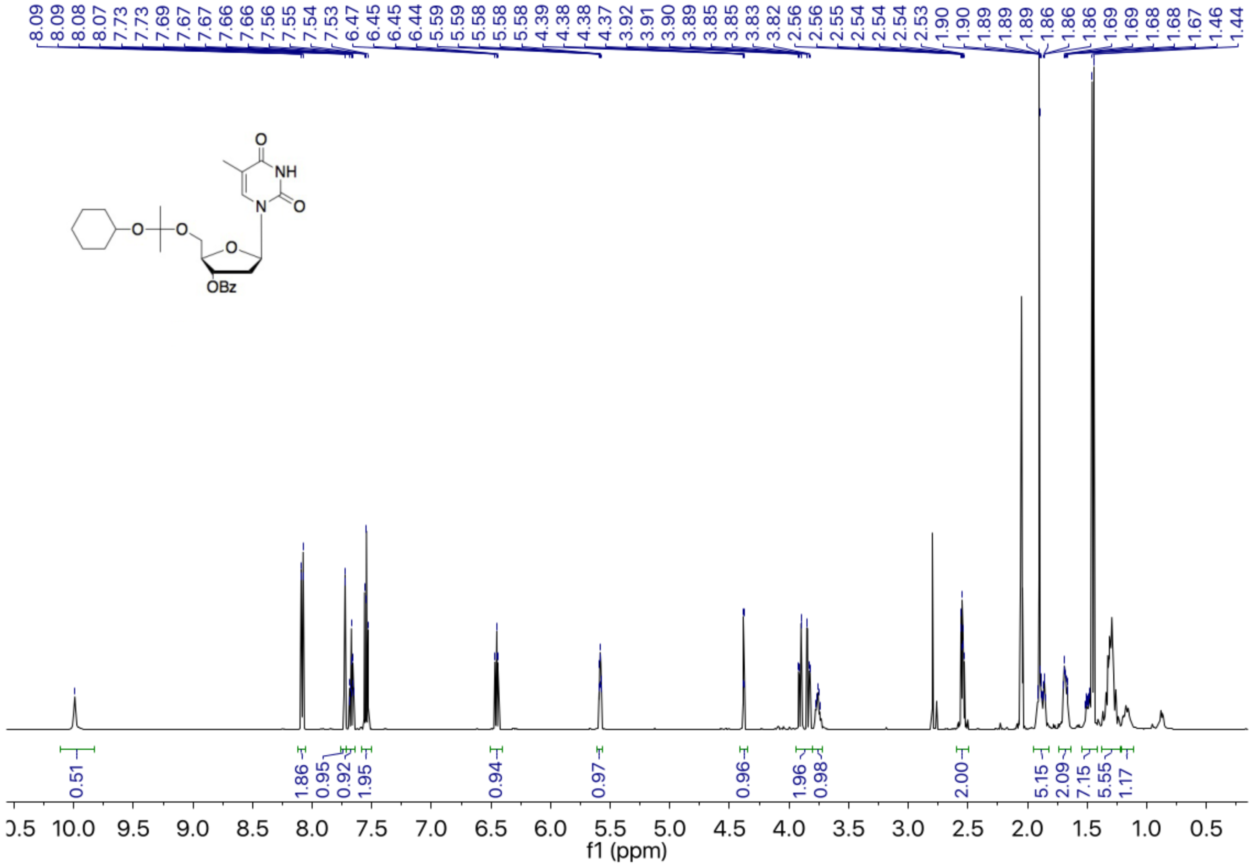

^{13}C-NMR (125 MHz, Acetone-d_6)

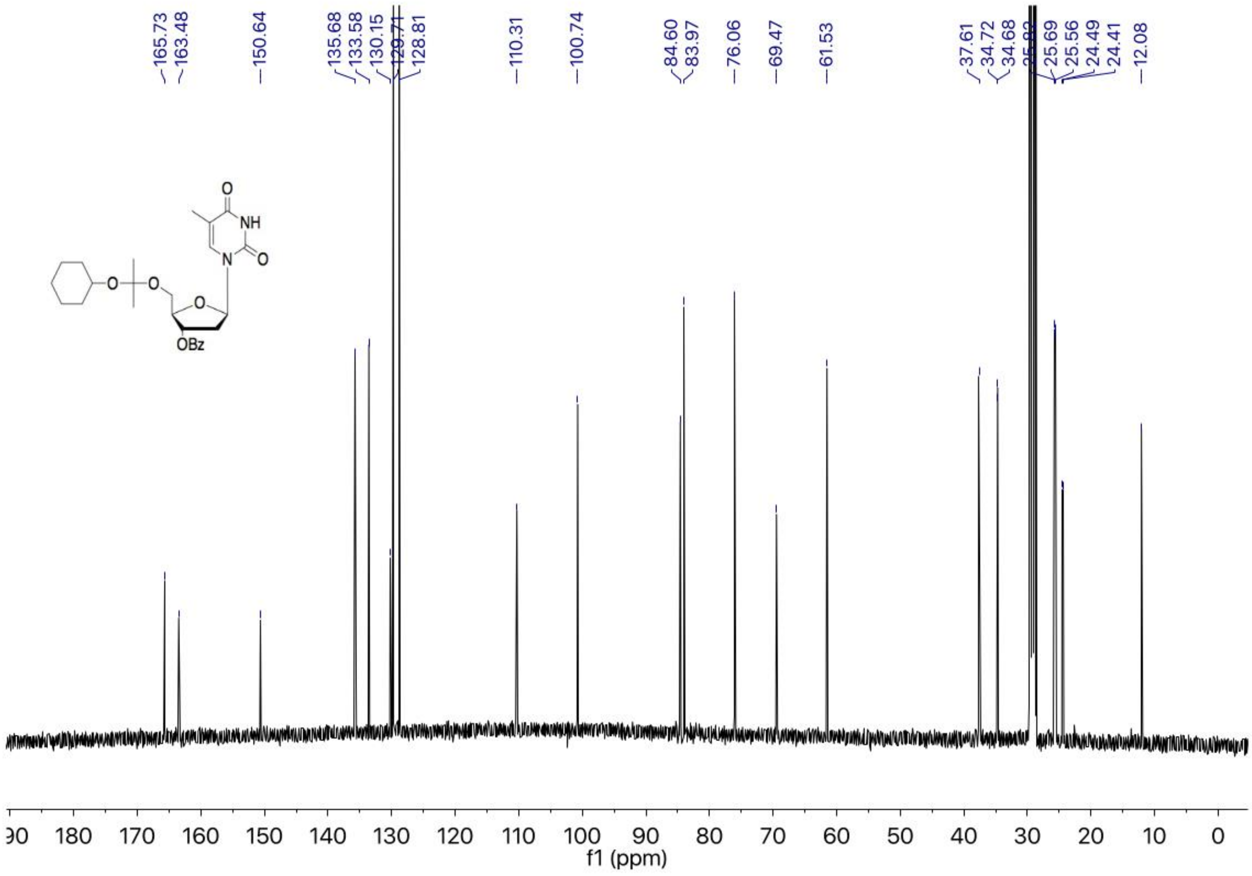

3’-O-Benzoyl-5’-O-isopropyloxypropan-2-yl-2’-deoxythymidine (**3d**).

^{1}H-NMR (500 MHz, DMSO-d_6)

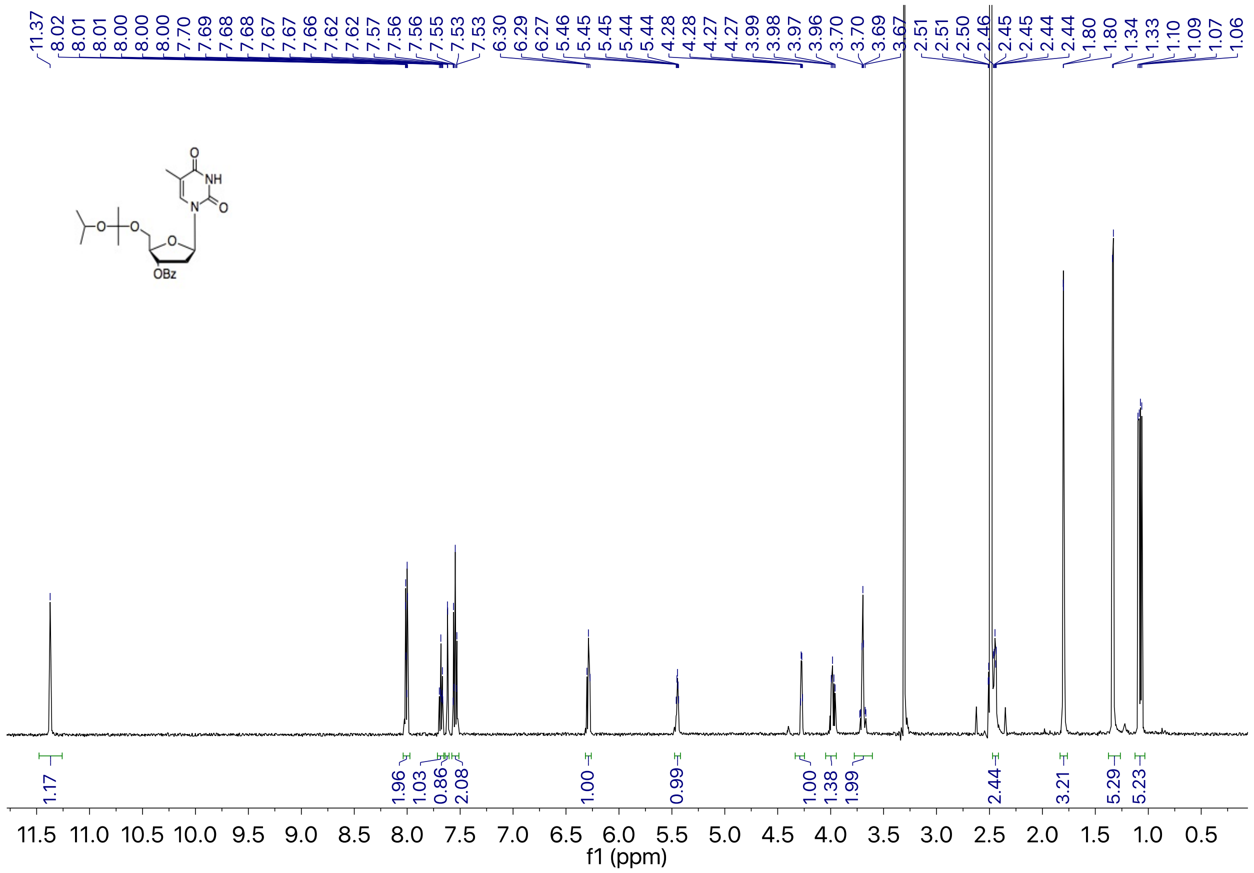

^{13}C-NMR (125 MHz, DMSO-d_6)

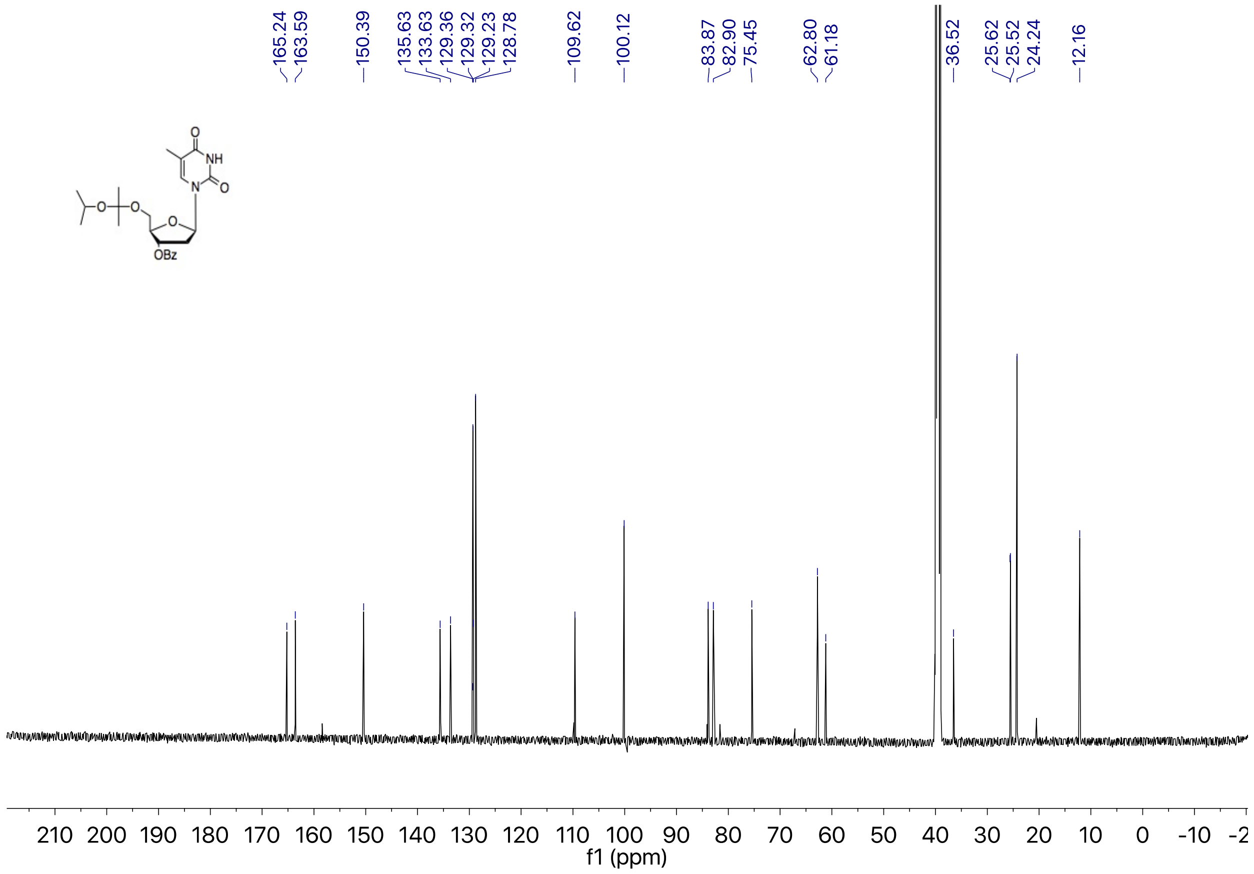

3'-O-Benzoyl-5'-O-(2,2,2-trifluoroethoxy)propan-2-yl-2'-deoxythymidine (**3e**).

^{1}H-NMR (500 MHz, DMSO-d_6)

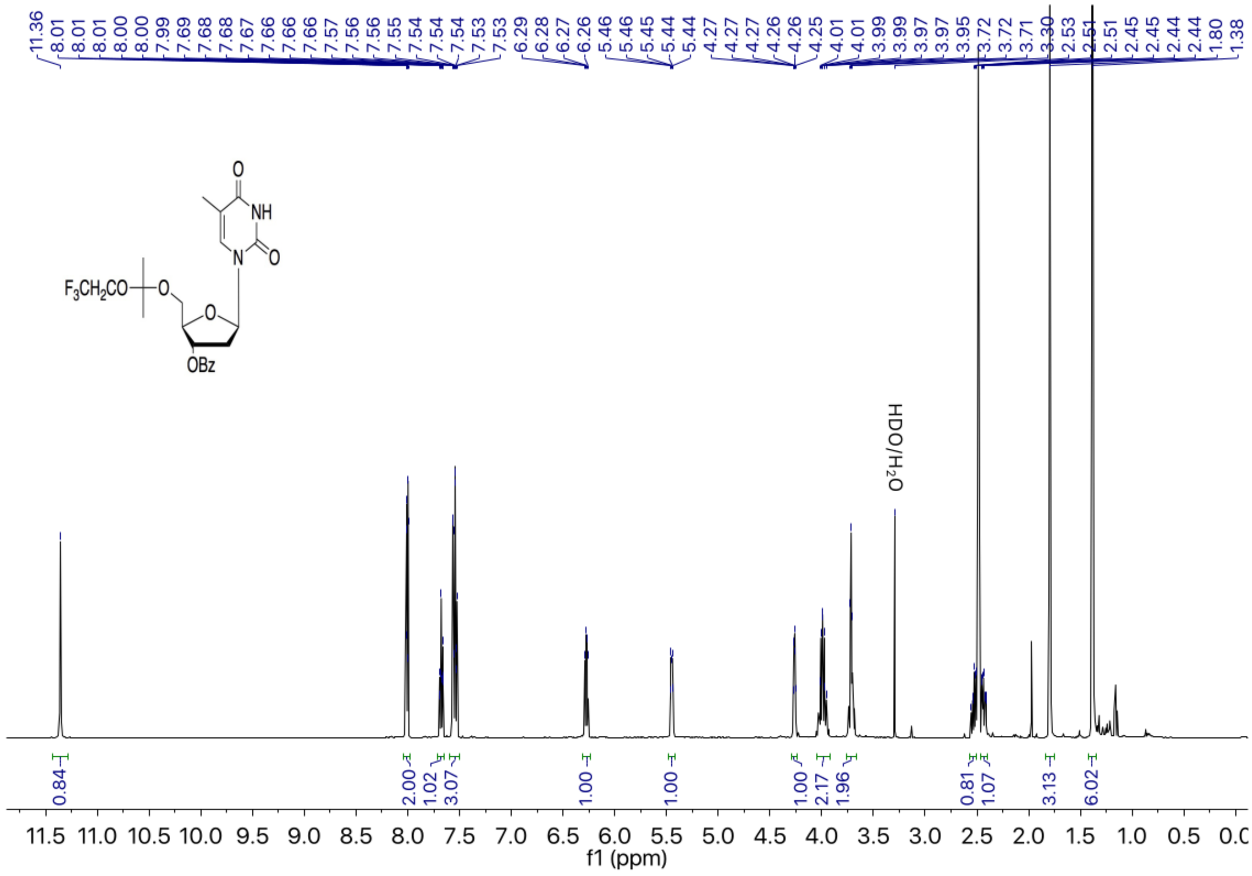

^{13}C-NMR (125 MHz, DMSO-d_6)

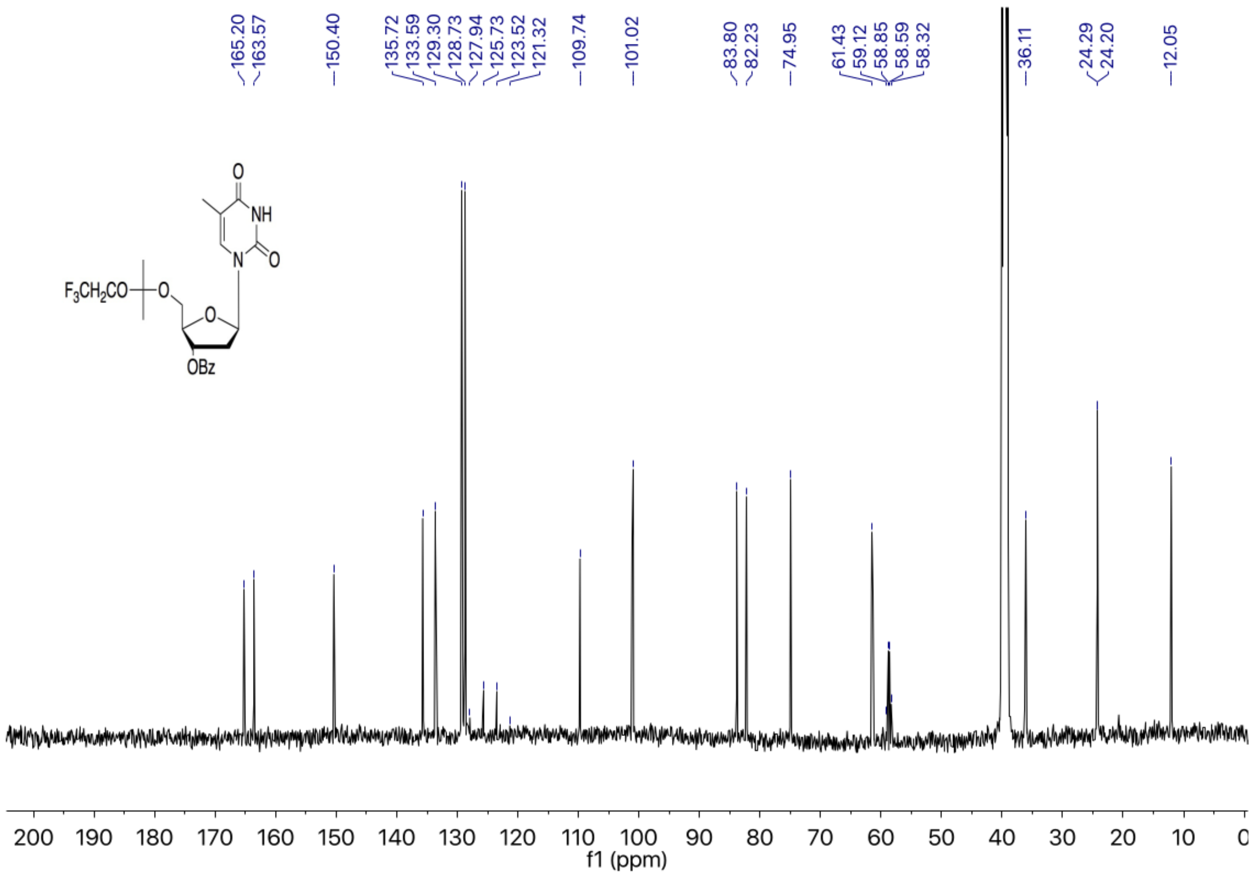

5'-O-Methoxypropan-2-yl-2'-deoxythymidine (**4a**).

^{1}H-NMR (500 MHz, DMSO-d_6)

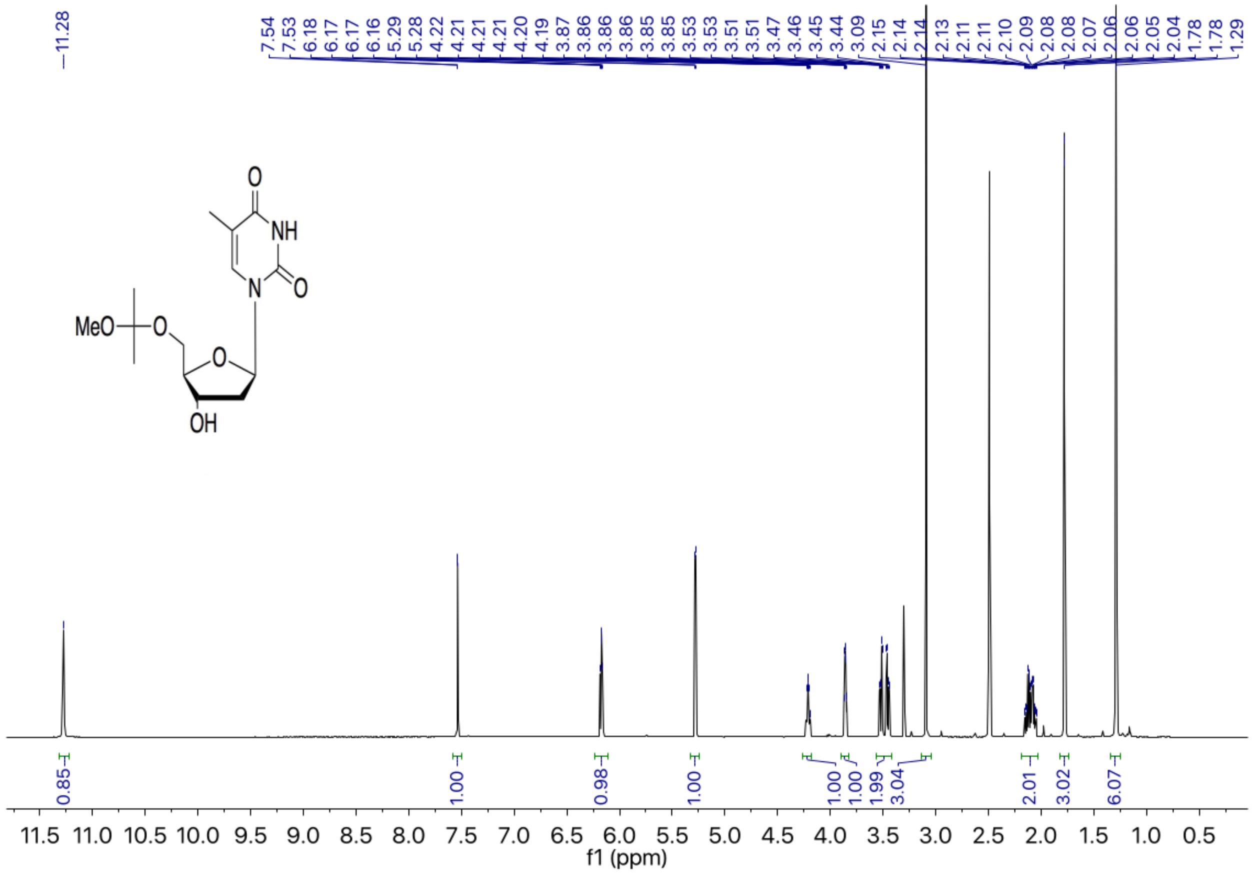

^{13}C-NMR (125 MHz, DMSO-d$_6$)

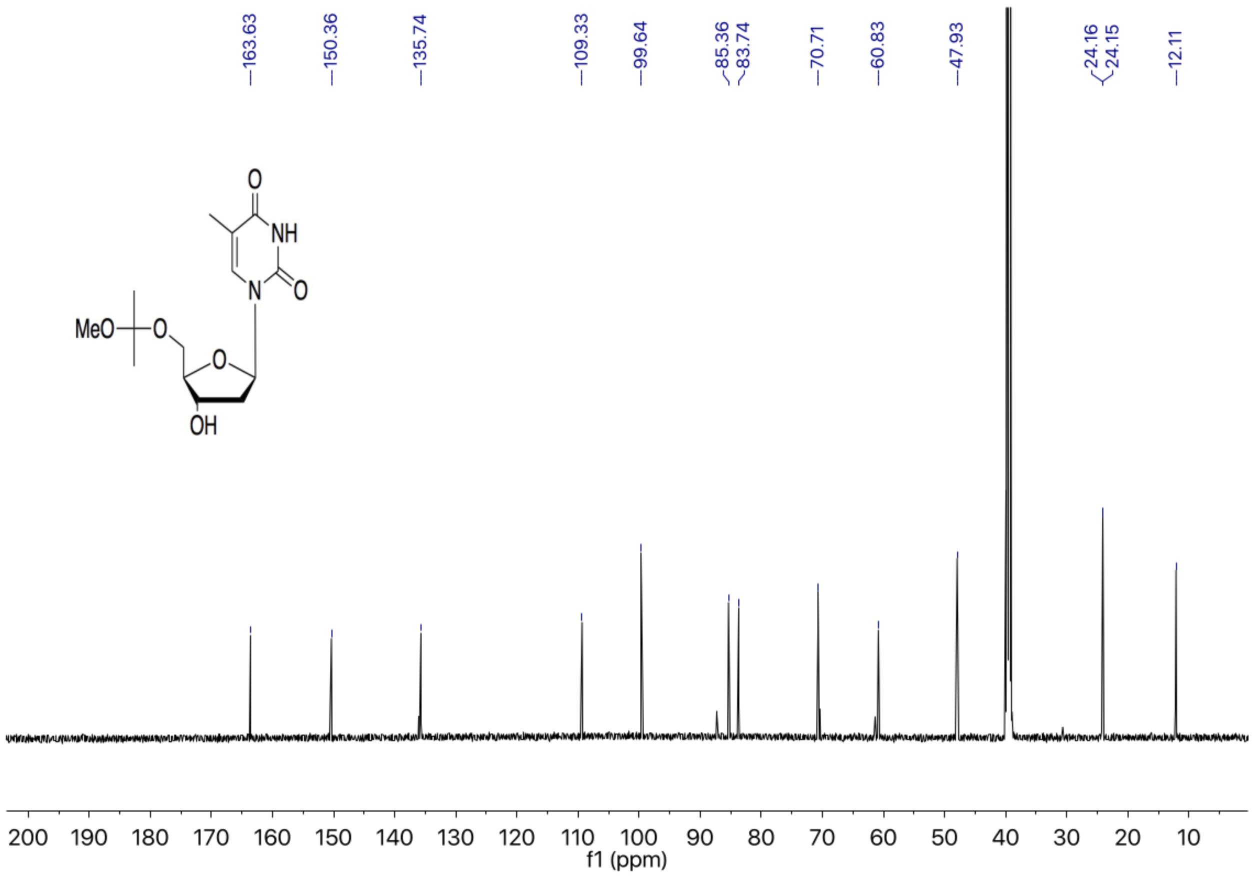

5'-O-Benzyloxypropan-2-yl-2'-deoxythymidine (**4b**).

^{1}H-NMR (500 MHz, DMSO-d_6)

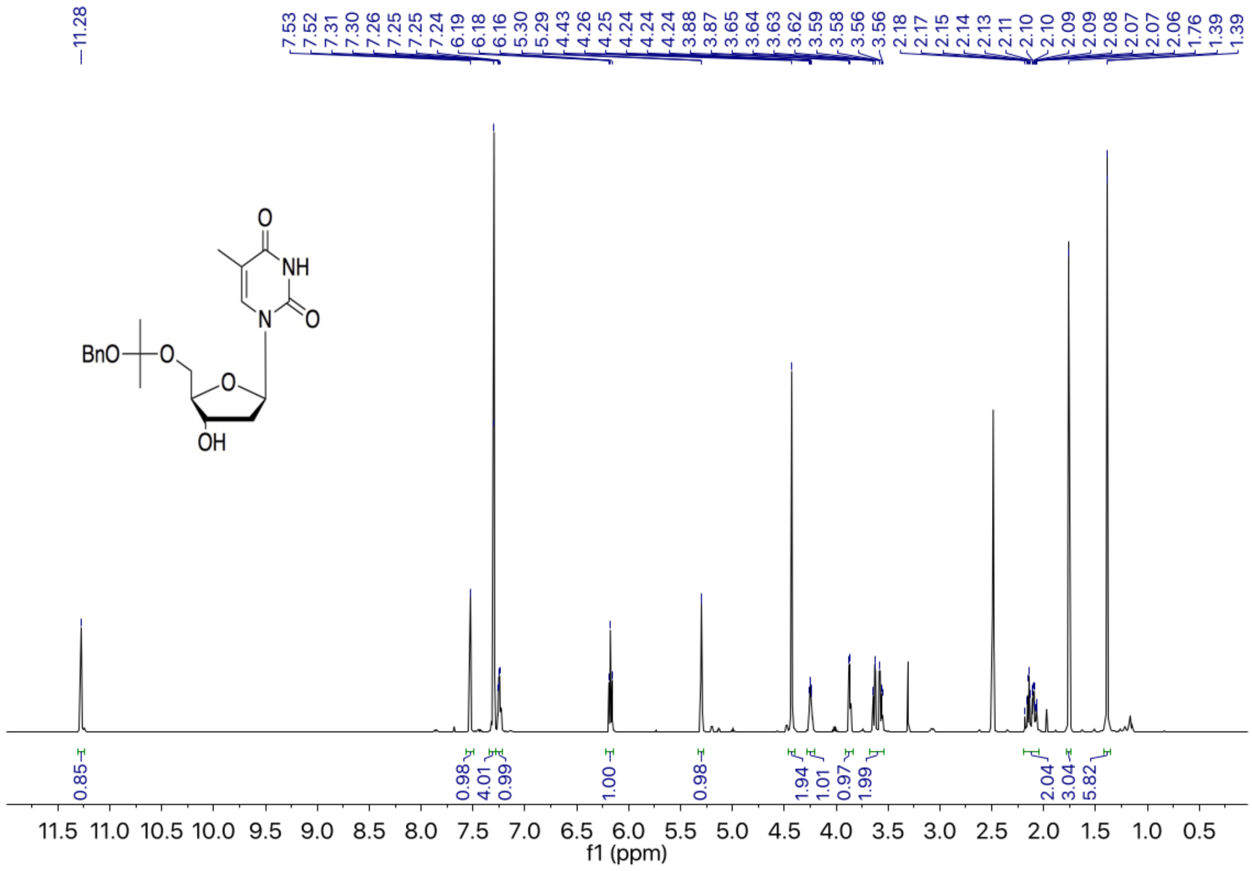

^{13}C-NMR (125 MHz, DMSO-d_6)

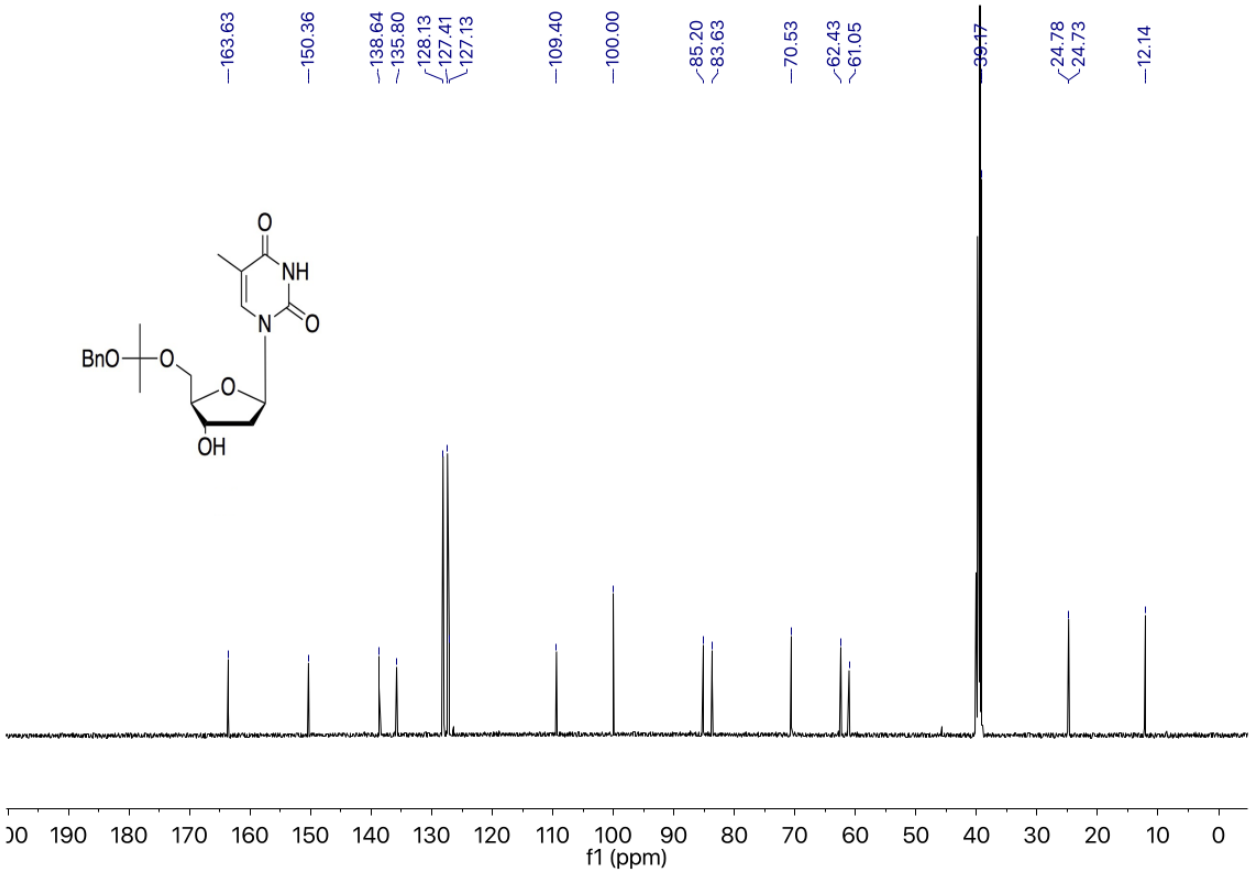

5'-O-Cyclohexyloxypropan-2-yl-2'-deoxythymidine (**4c**).
^{1}H-NMR (500 MHz, DMSO-d$_6$)

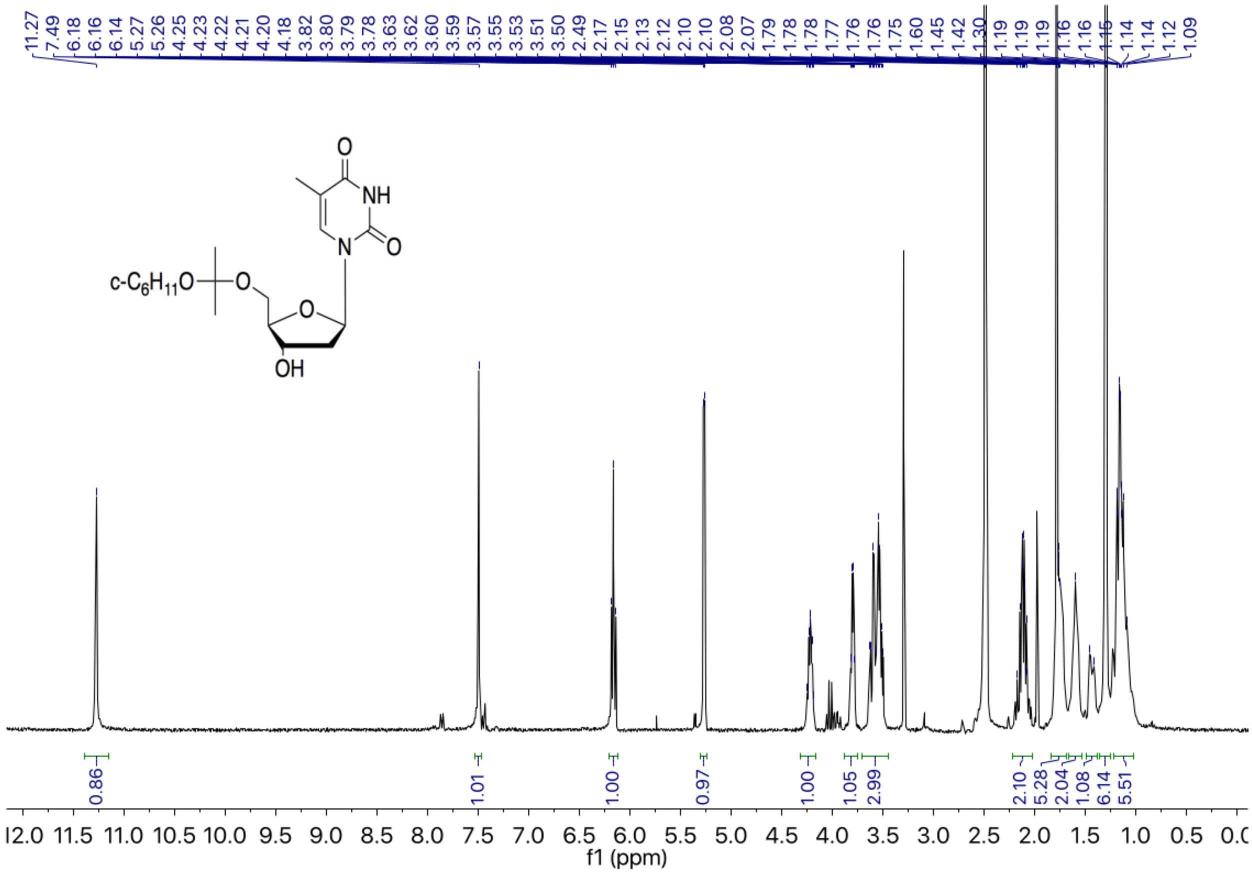

^{13}C-NMR (75 MHz, DMSO-d$_6$)

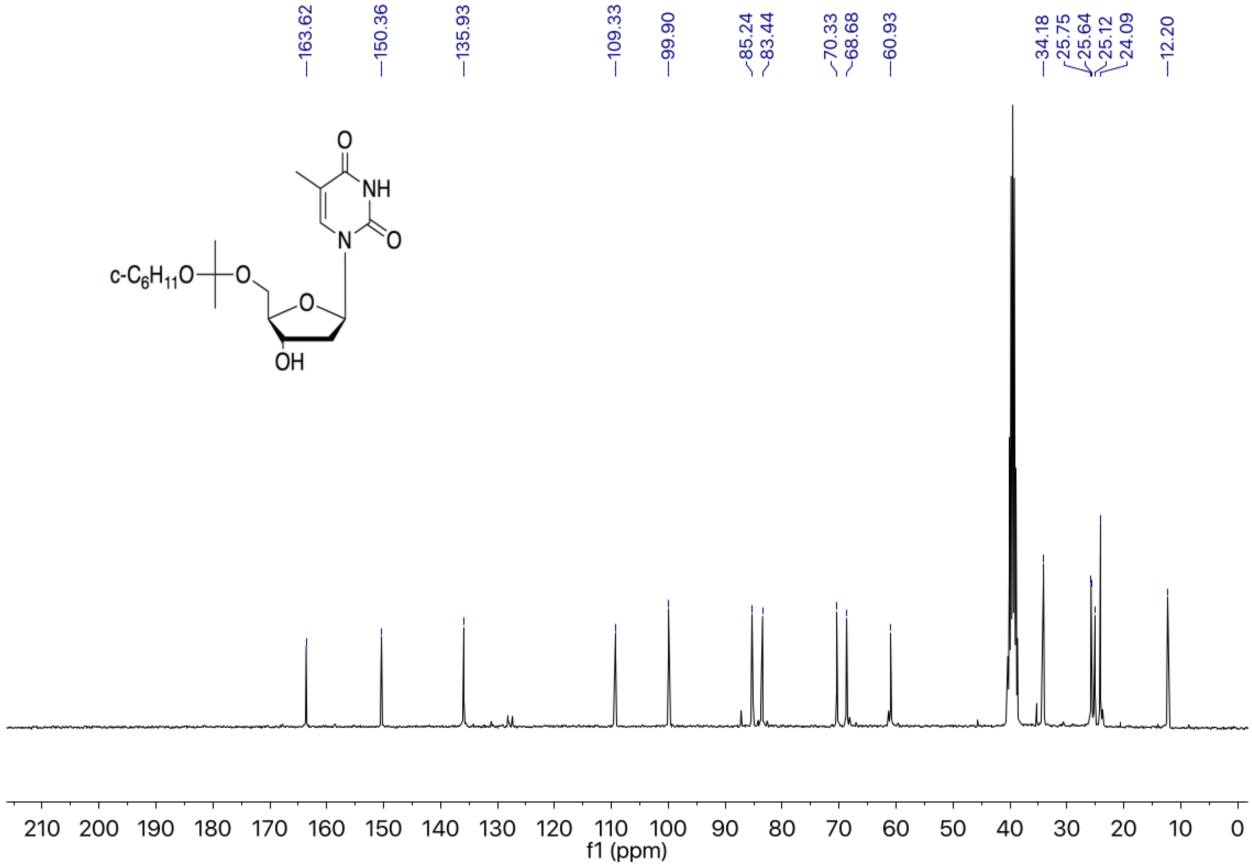

5'-O-Isopropyloxypropan-2-yl-2'-deoxythymidine (**4d**).

^{1}H-NMR (600 MHz, DMSO-d_6)

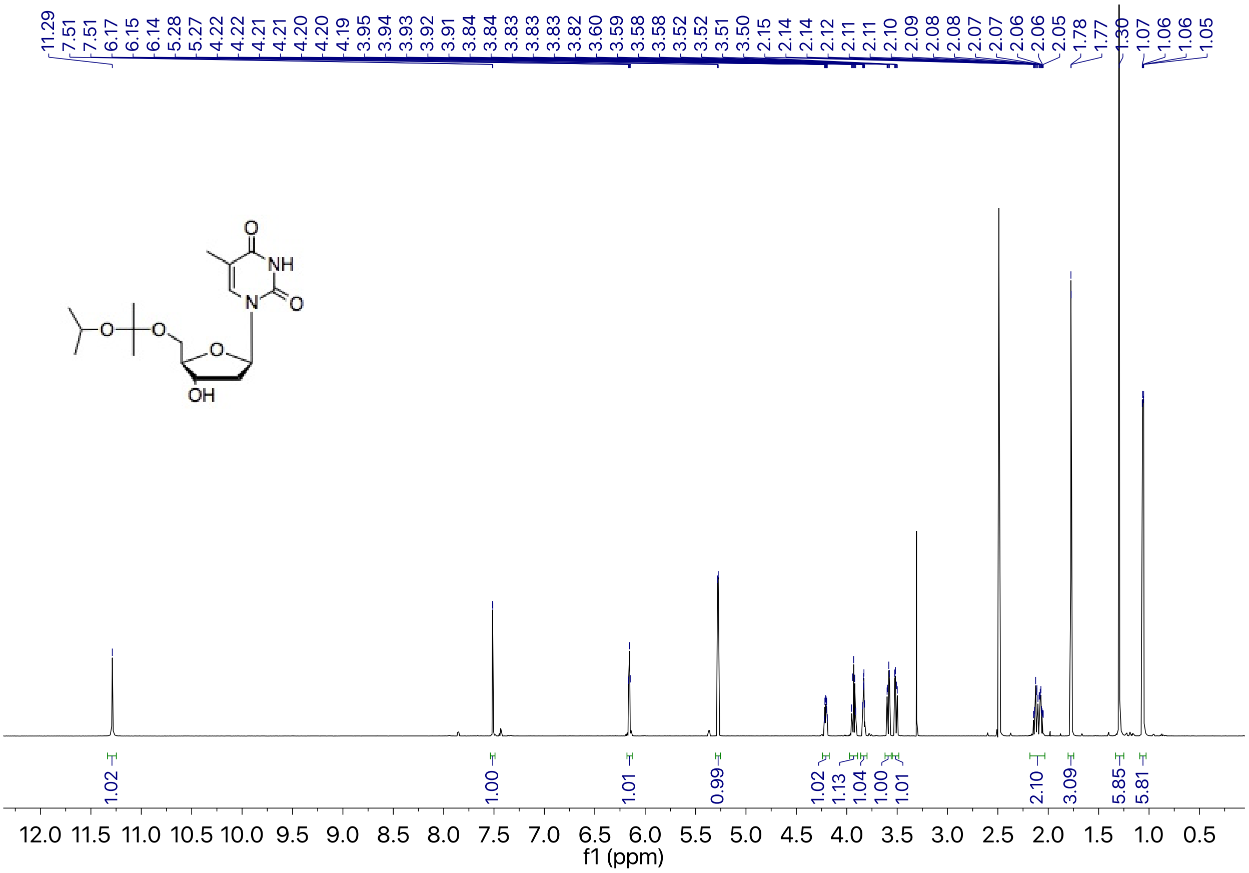

^{13}C-NMR (150 MHz, DMSO-d_6)

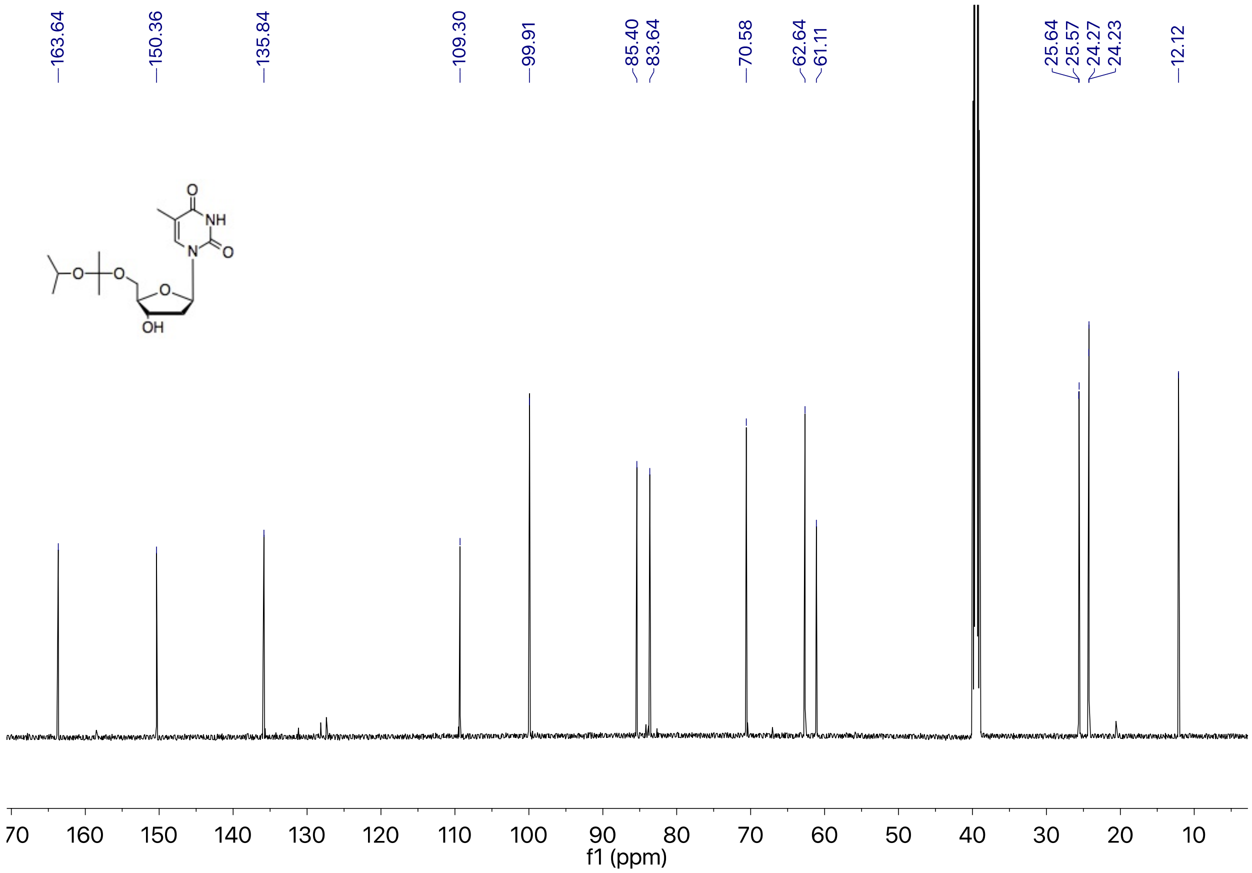

5'-O-(2,2,2-Trifluoroethoxy)propan-2-yl-2'-deoxythymidine (**4e**).

^{1}H-NMR (500 MHz, DMSO-d$_6$)

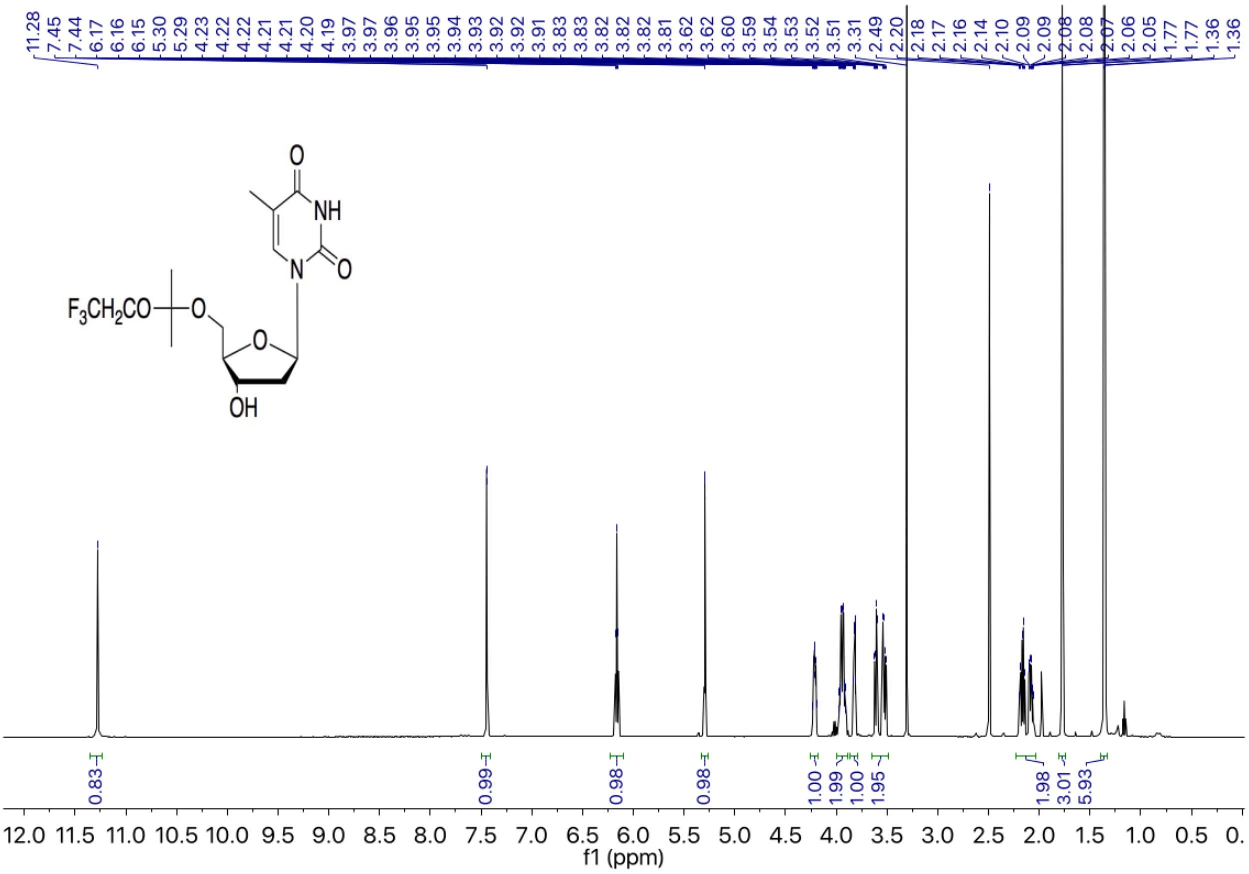

^{13}C-NMR (125 MHz, DMSO-d_6)

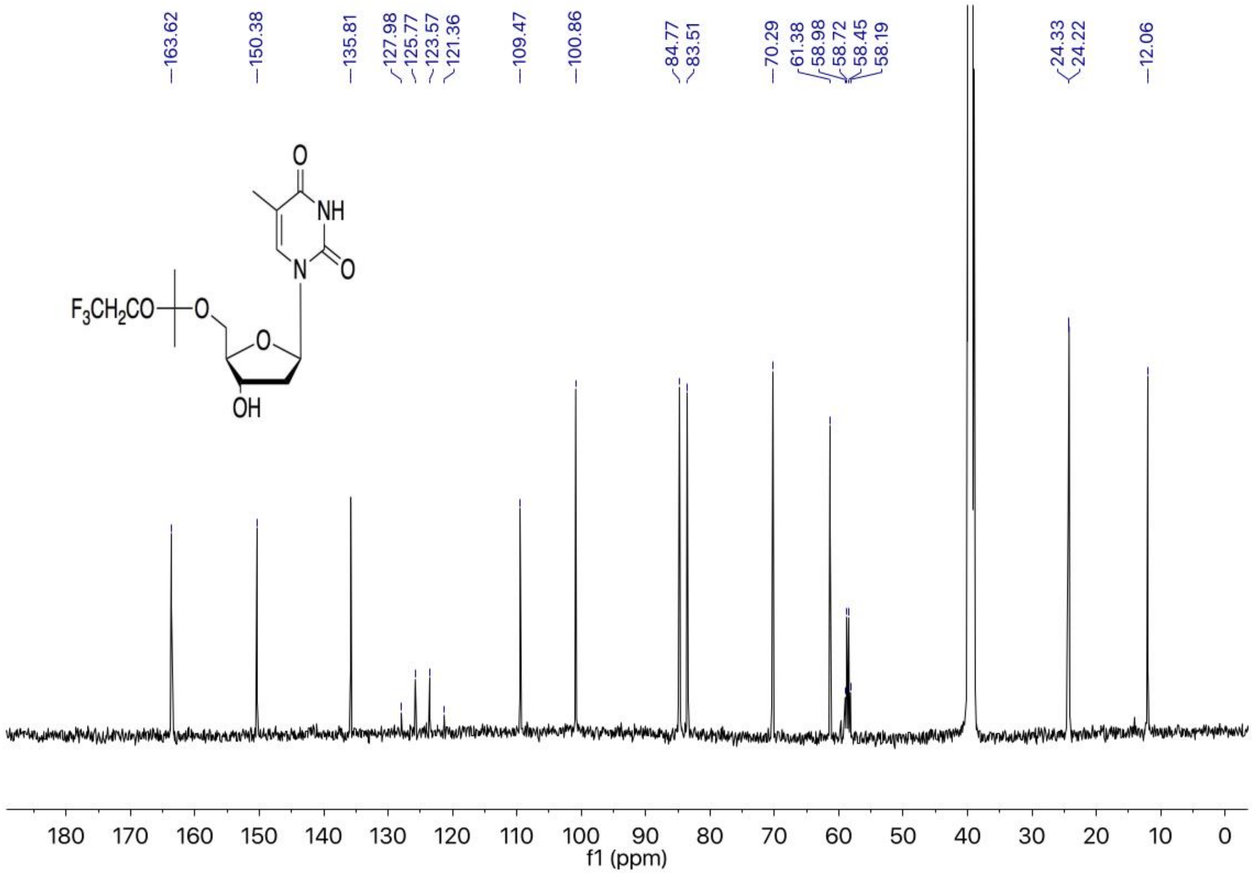

3'-O-Methoxypropan-2-yl-5'-O-tert-butyldimethylsilyl-2'-deoxythymidine (6**a**).

^{1}H-NMR (500 MHz, DMSO-d_6)

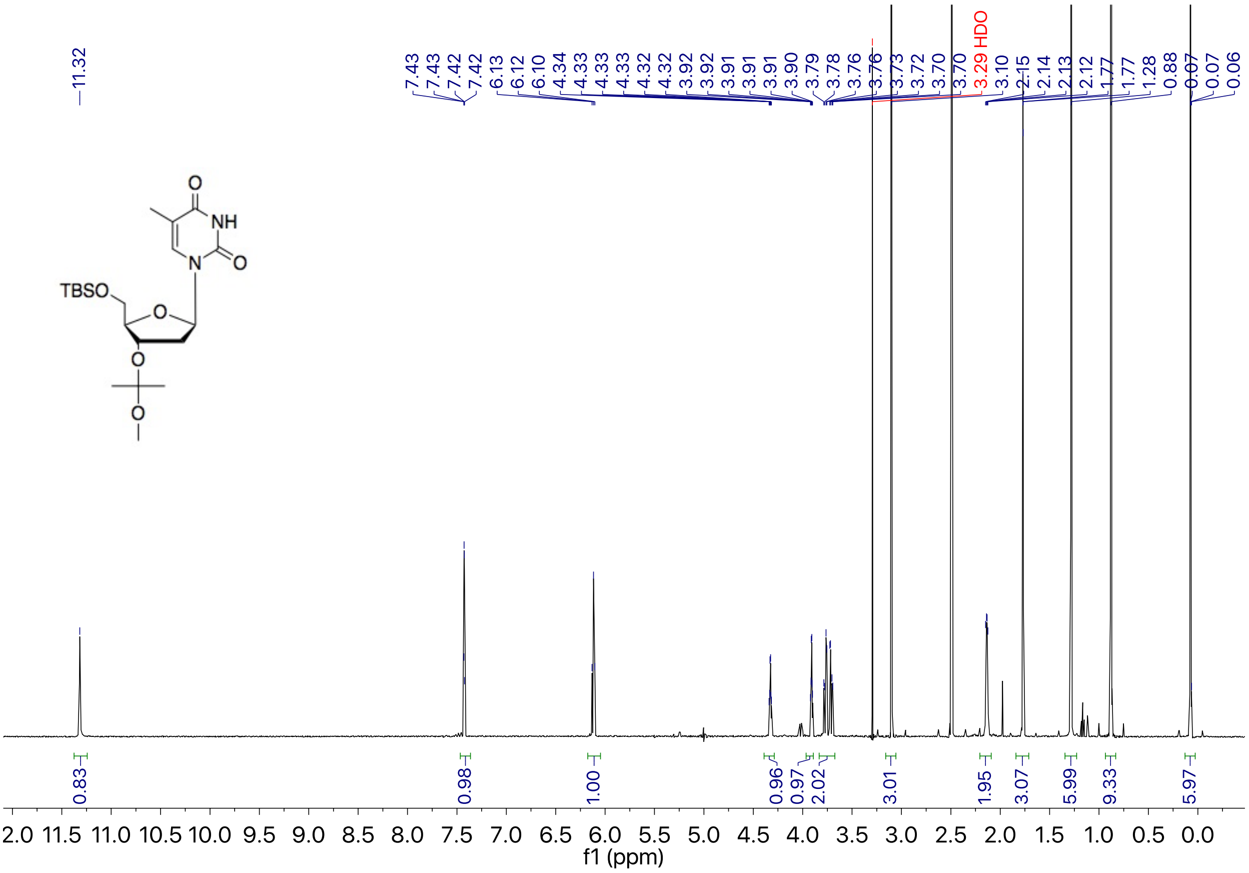

^{13}C-NMR (125 MHz, DMSO-d$_6$)

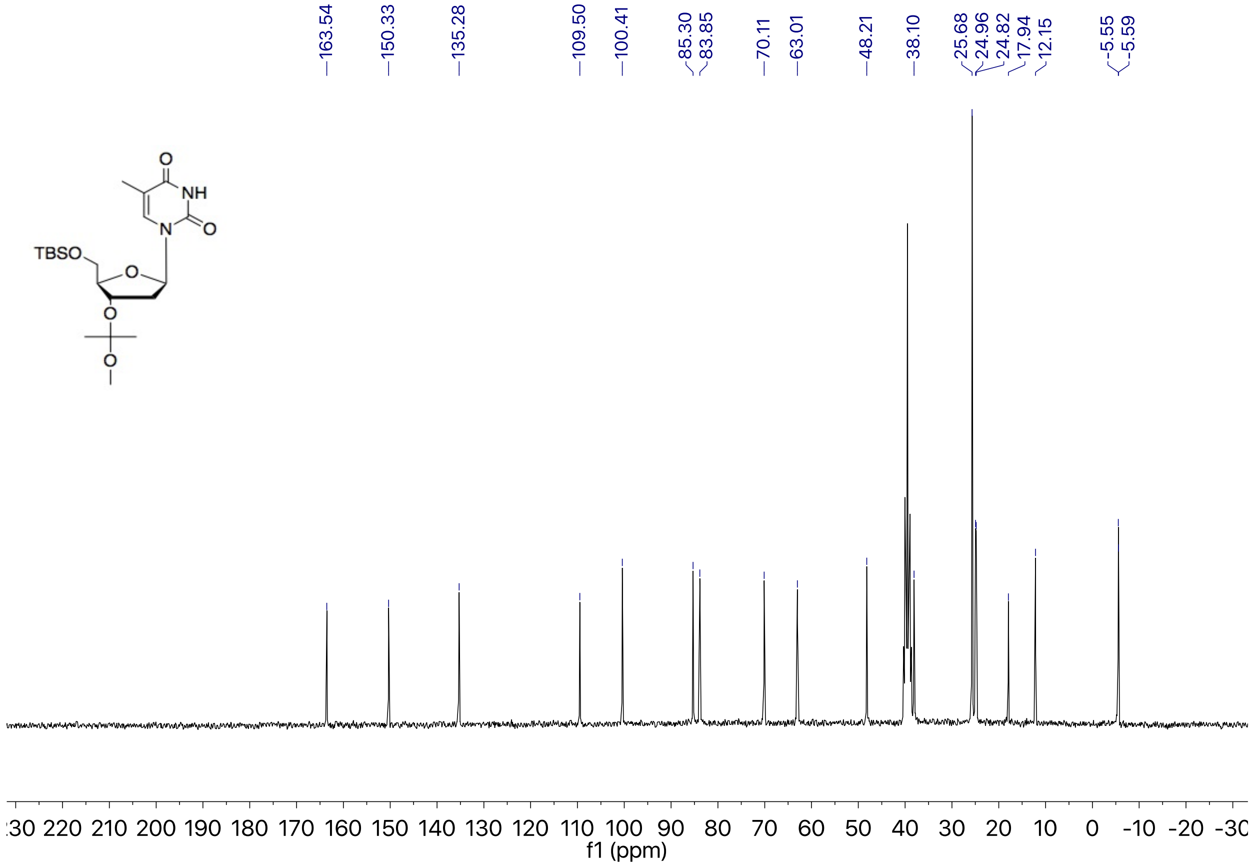

3'-O-Cyclohexyloxypropan-2-yl-5'-O-tert-butyldimethylsilyl-2'-deoxythymidine (**6c**).

^{1}H-NMR (500 MHz, DMSO-d_6)

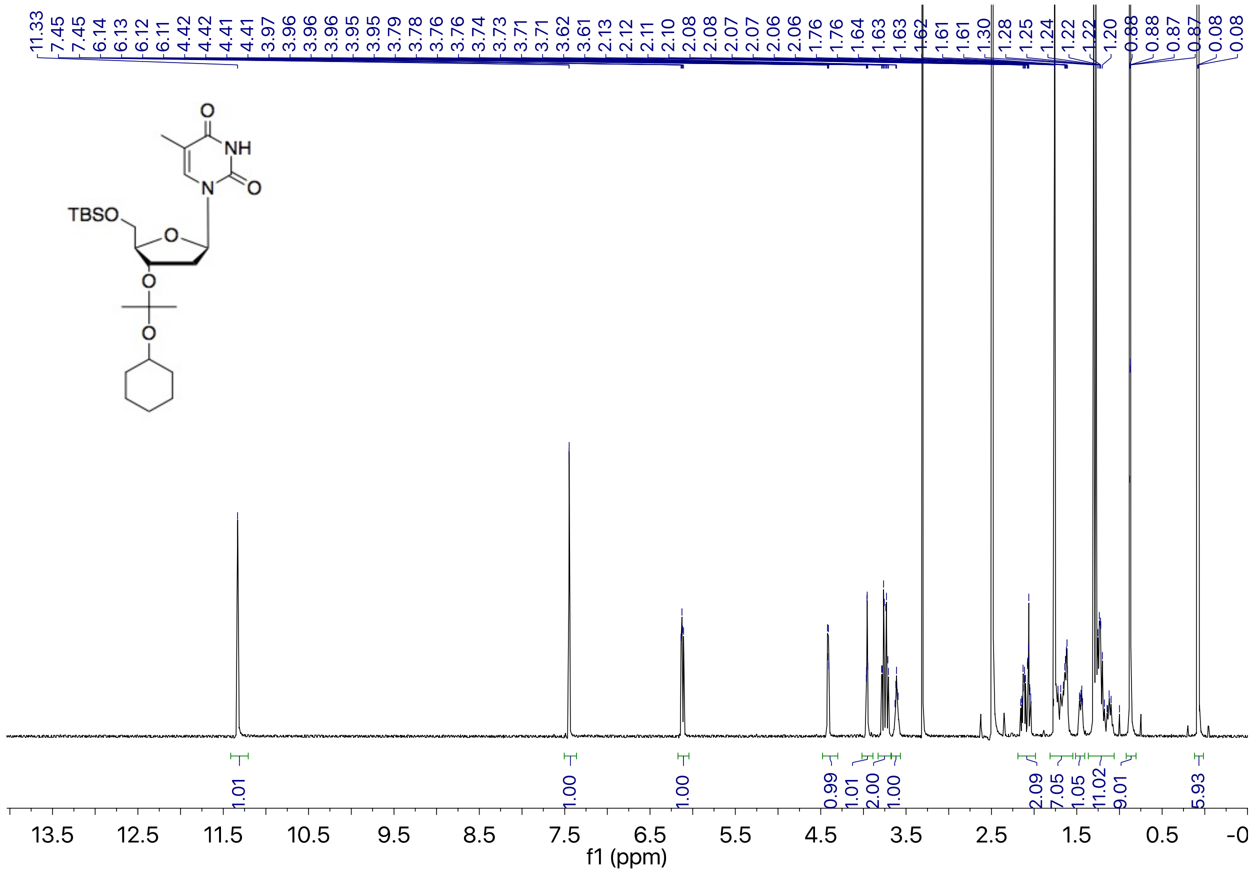

^{13}C-NMR (125 MHz, DMSO-d$_6$)

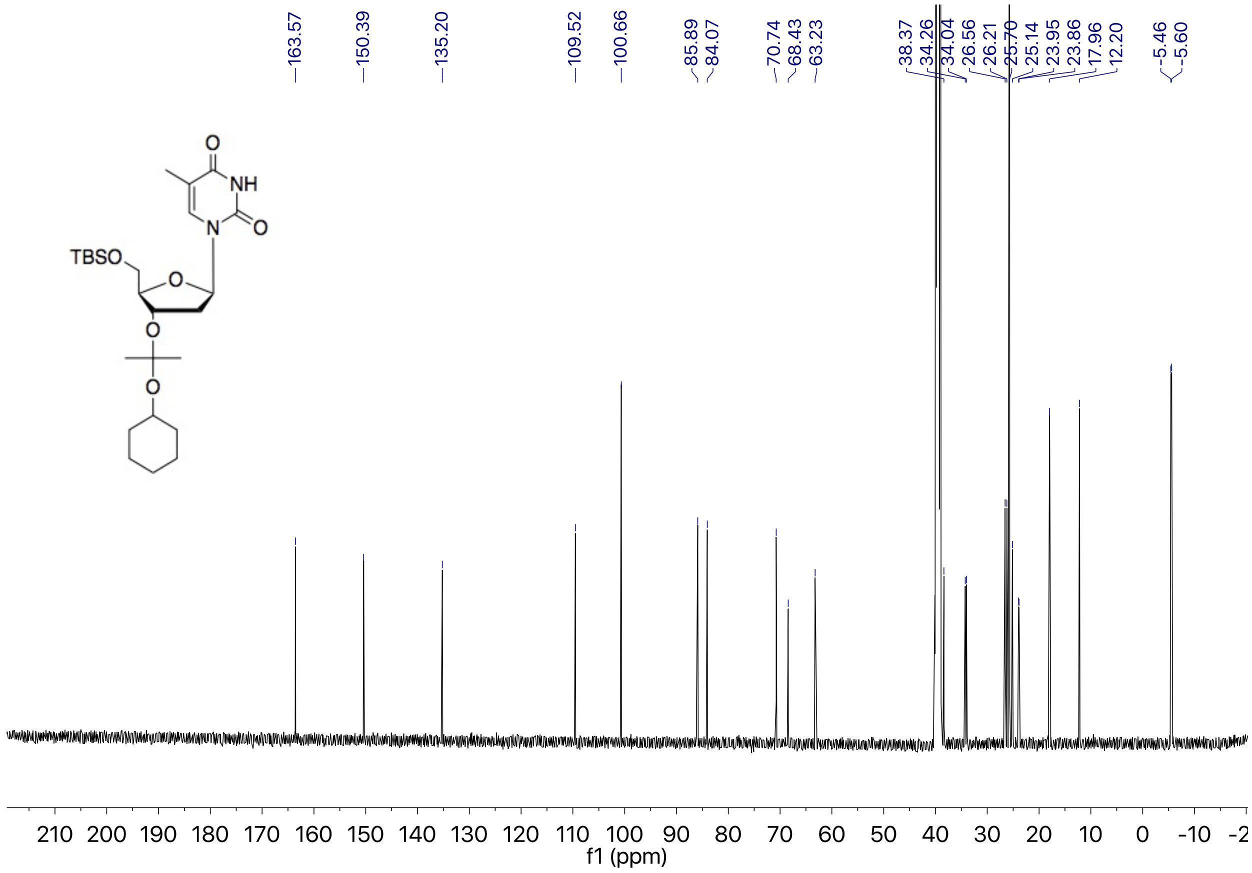

3'-O-Isopropyloxypropan-2-yl-5'-O-tert-butyldimethylsilyl-2'-deoxythymidine (**6d**).

^{1}H-NMR (500 MHz, $CDCl_3$):

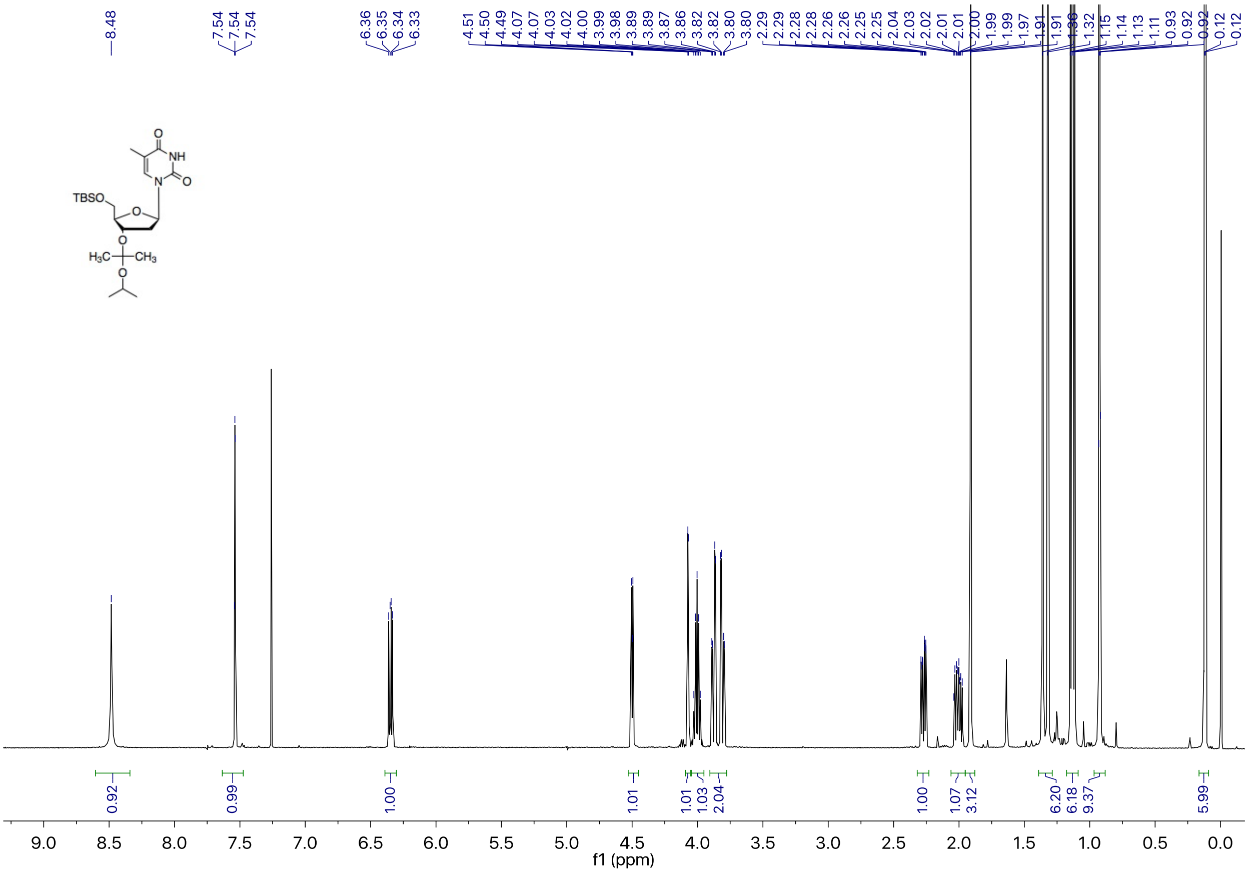

^{13}C-NMR (125 MHz, $CDCl_3$):

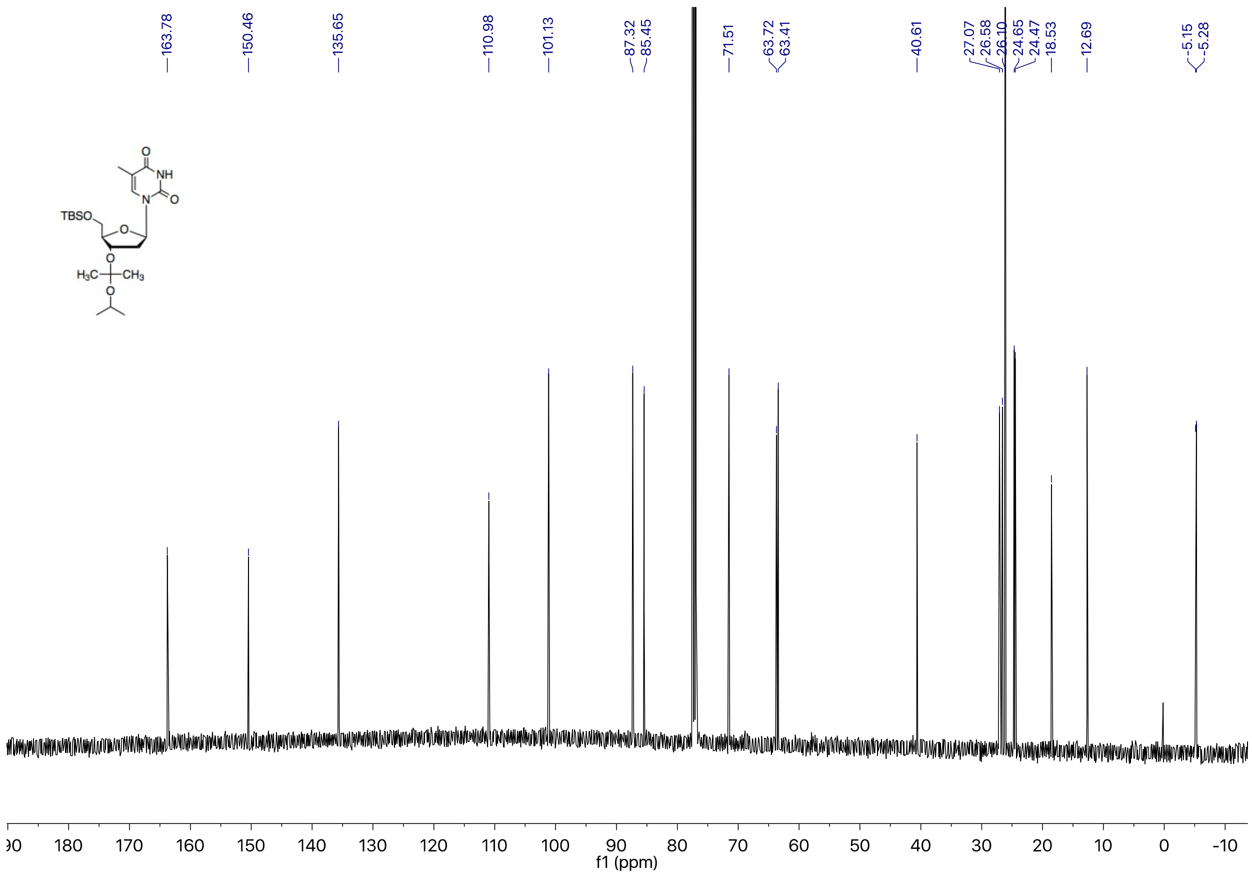

3'-O-(2,2,2-Trifluoroethoxy)propan -2-yl-5'-O-tert-butyldimethylsilyl-2'-deoxythymidine (**6e**).

^{1}H-NMR (600 MHz, DMSO-d6):

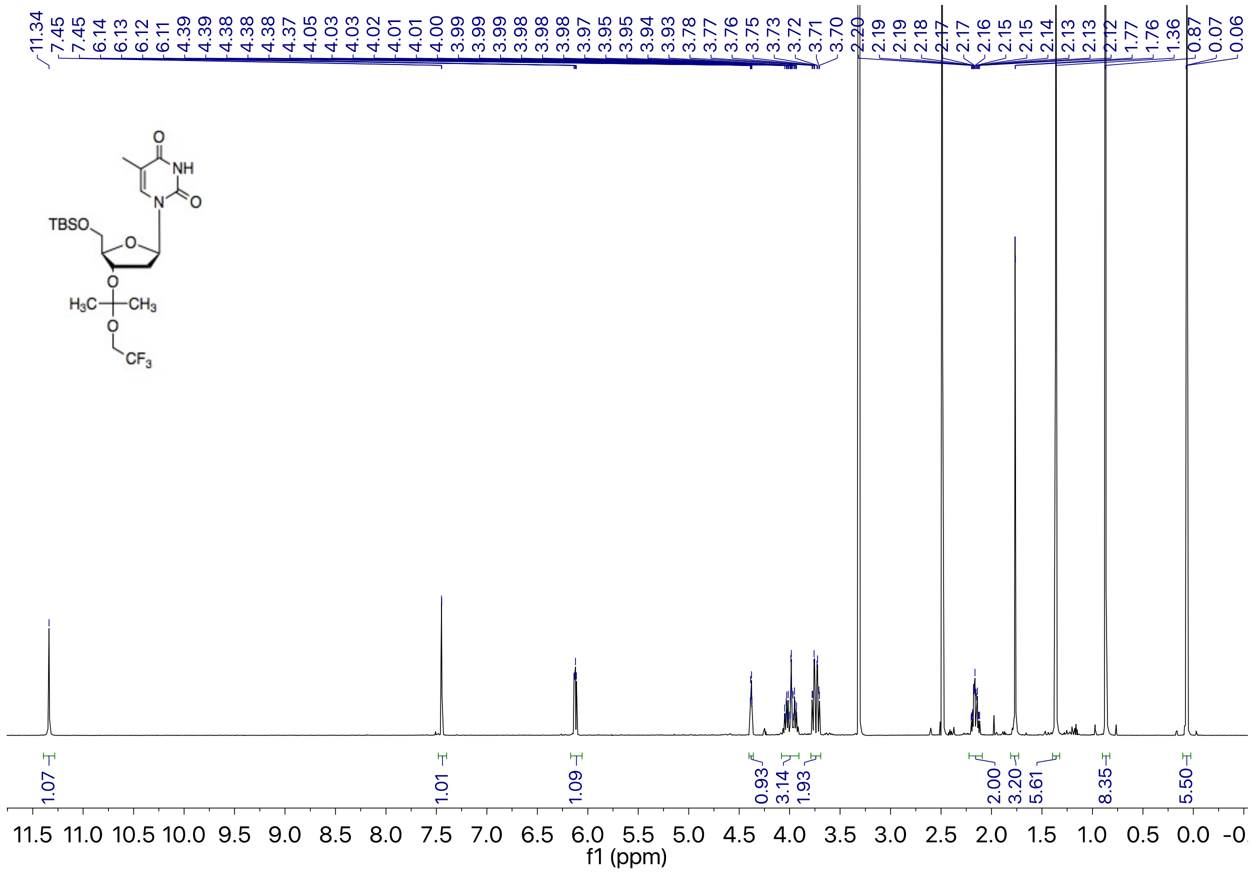

^{13}C-NMR (125 MHz, DMSO-d$_6$)

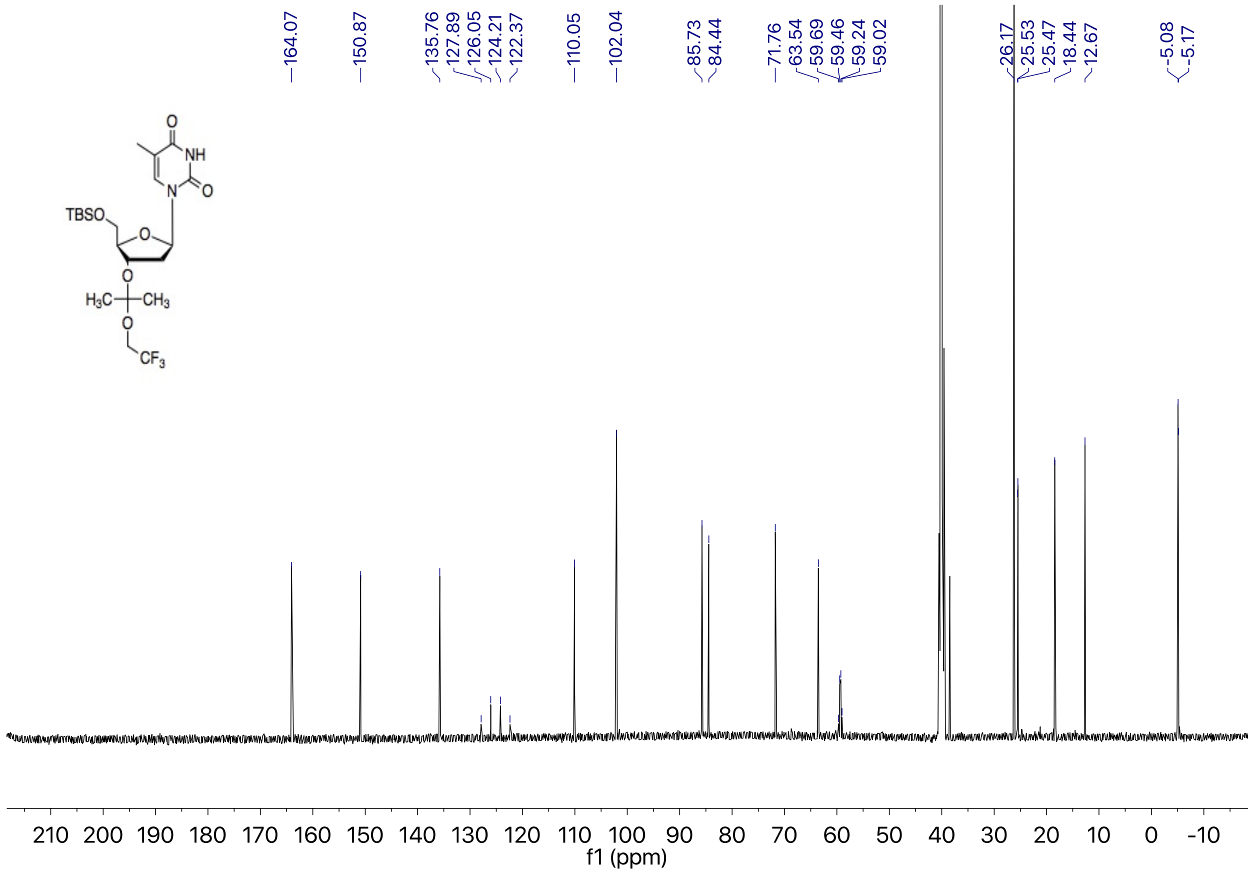

3'-O-Methoxypropan-2-yl-2'-deoxythymidine (**7a**).

^{1}H-NMR (500 MHz, DMSO-d$_6$)

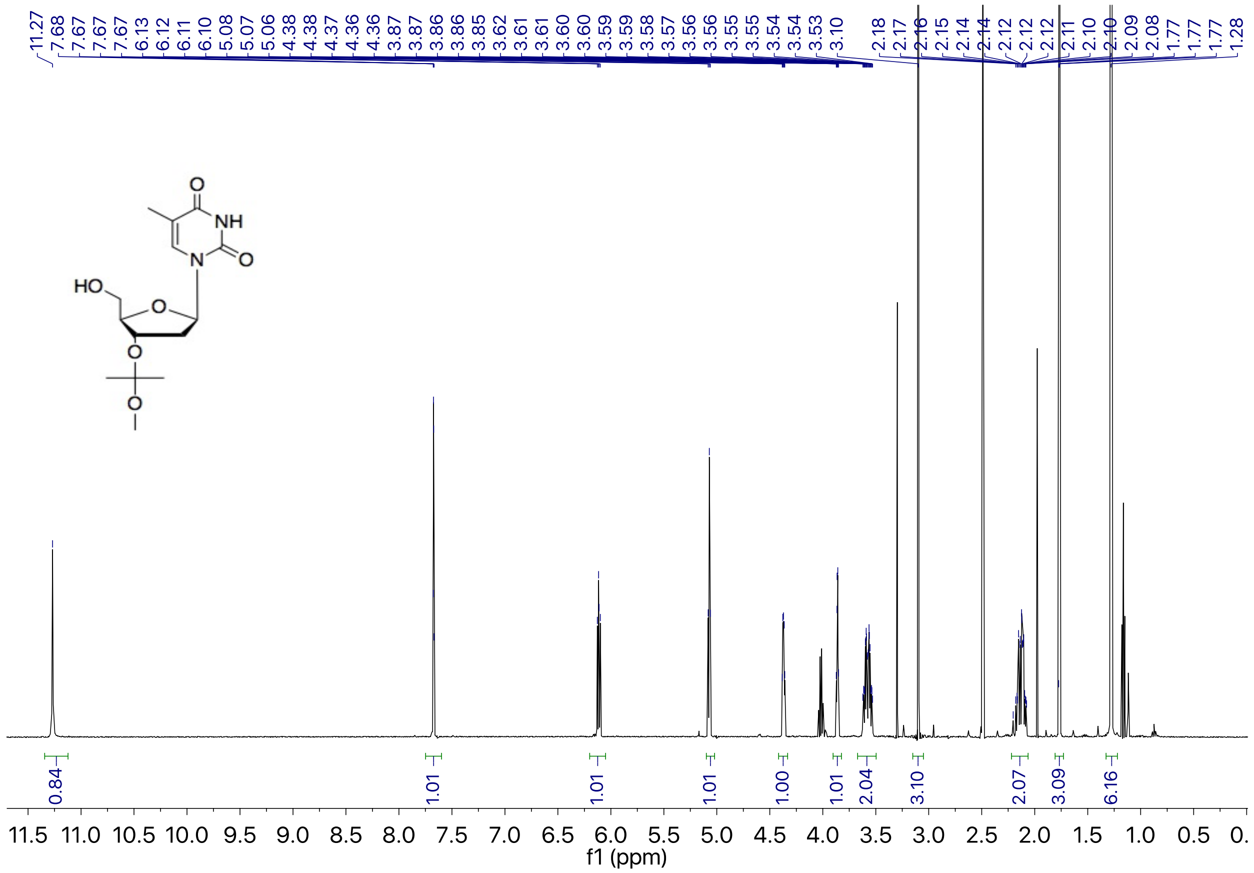

^{13}C-NMR (125 MHz, DMSO-d_6)

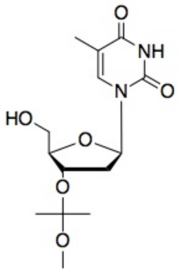

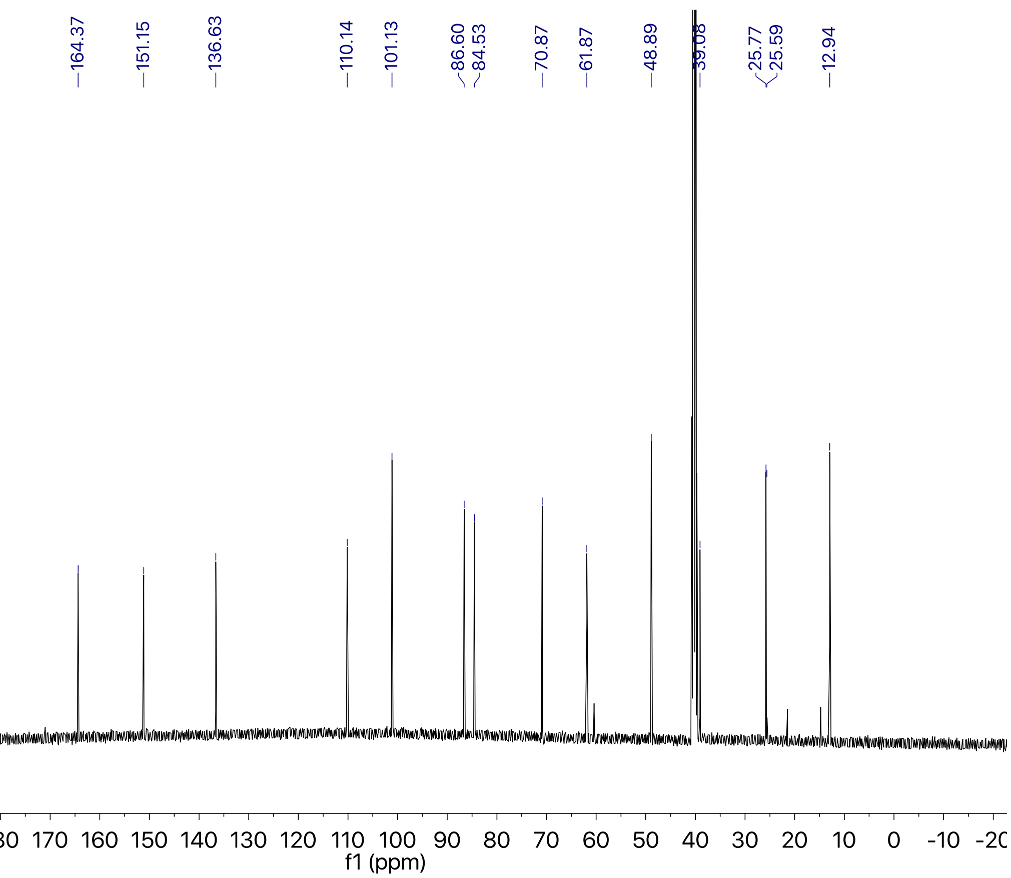

3'-O-Cyclohexyloxypropan-2-yl-2'-deoxythymidine (**7c**).

^{1}H-NMR (500 MHz, Acetone-d$_6$)

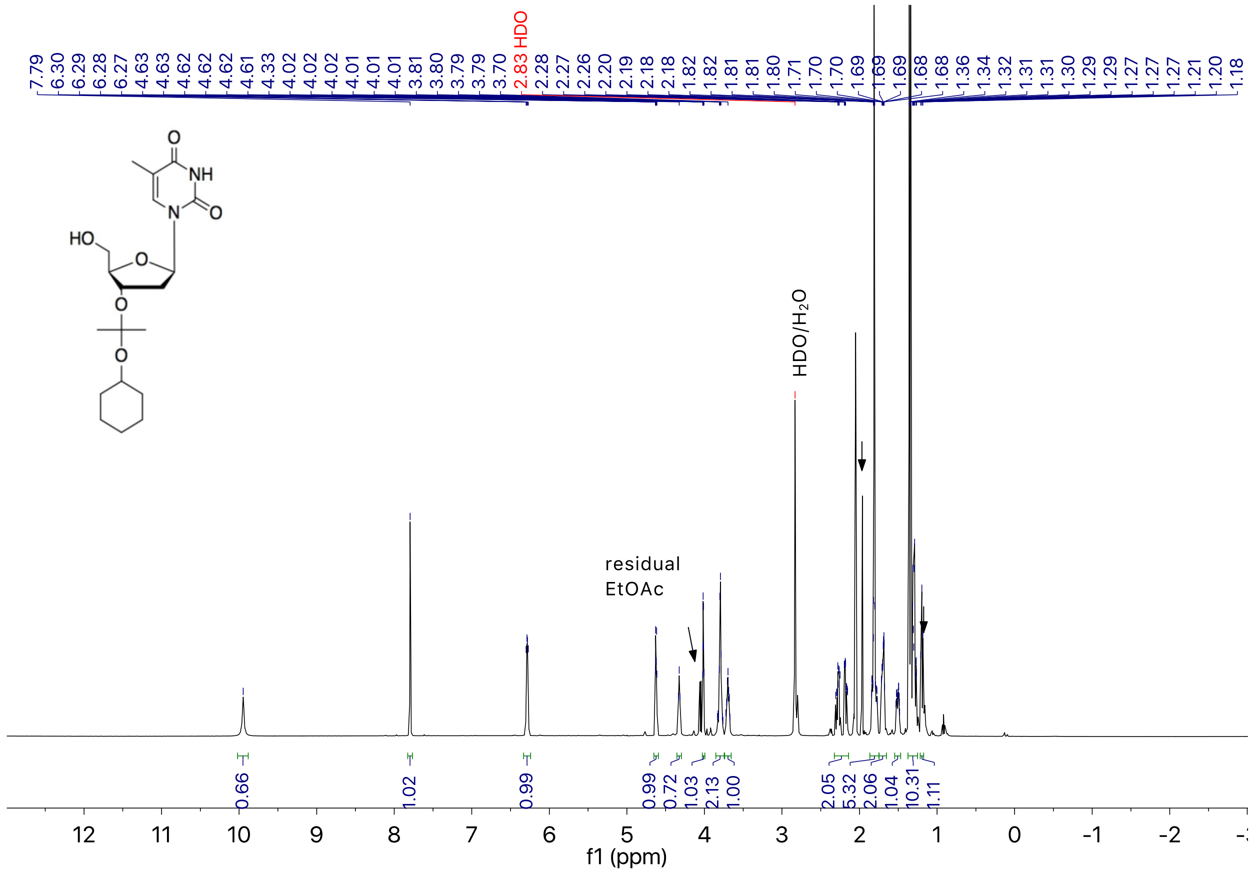

^{13}C-NMR (125 MHz, Acetone-d_6)

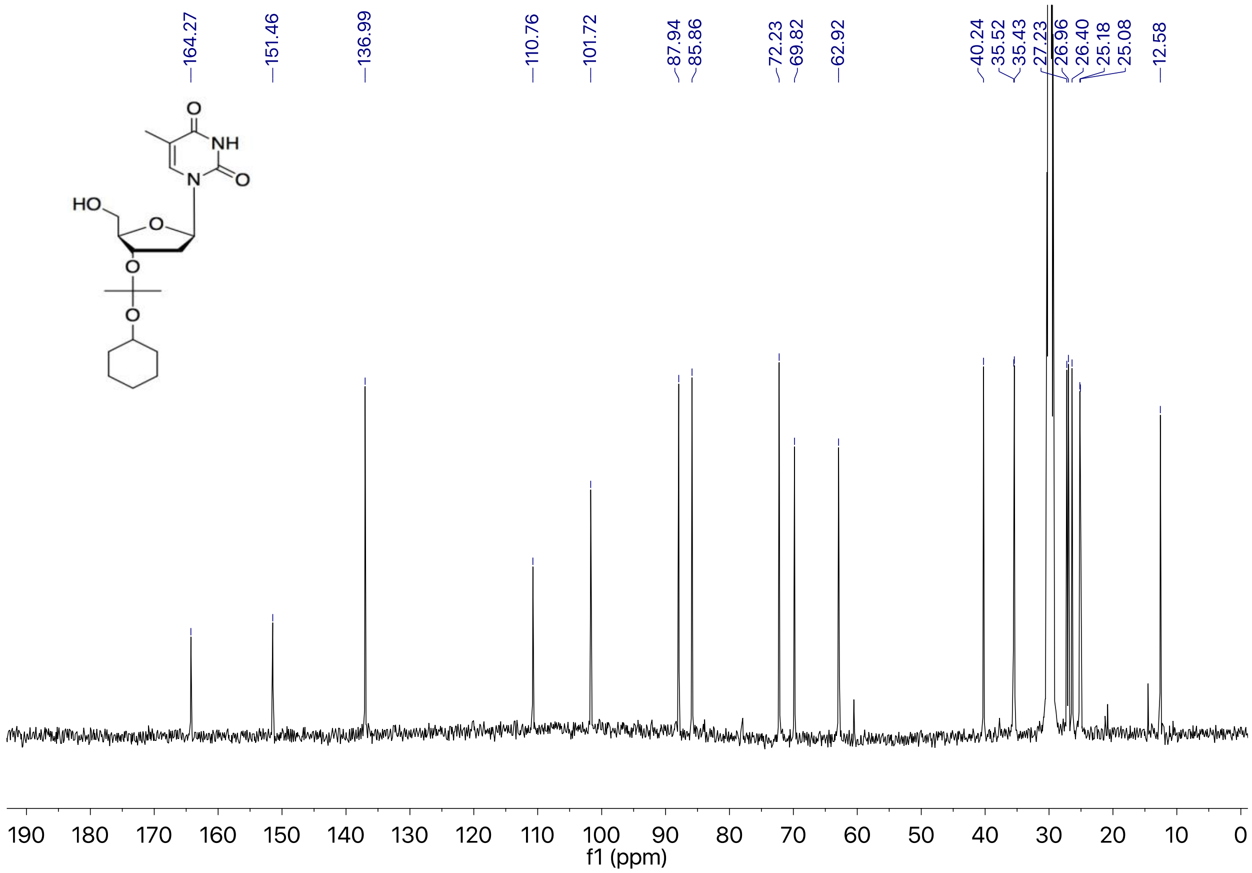

3'-O-Isopropyloxypropan-2-yl-2'-deoxythymidine (**7d**).

^{1}H-NMR (500 MHz, Acetone-d_6)

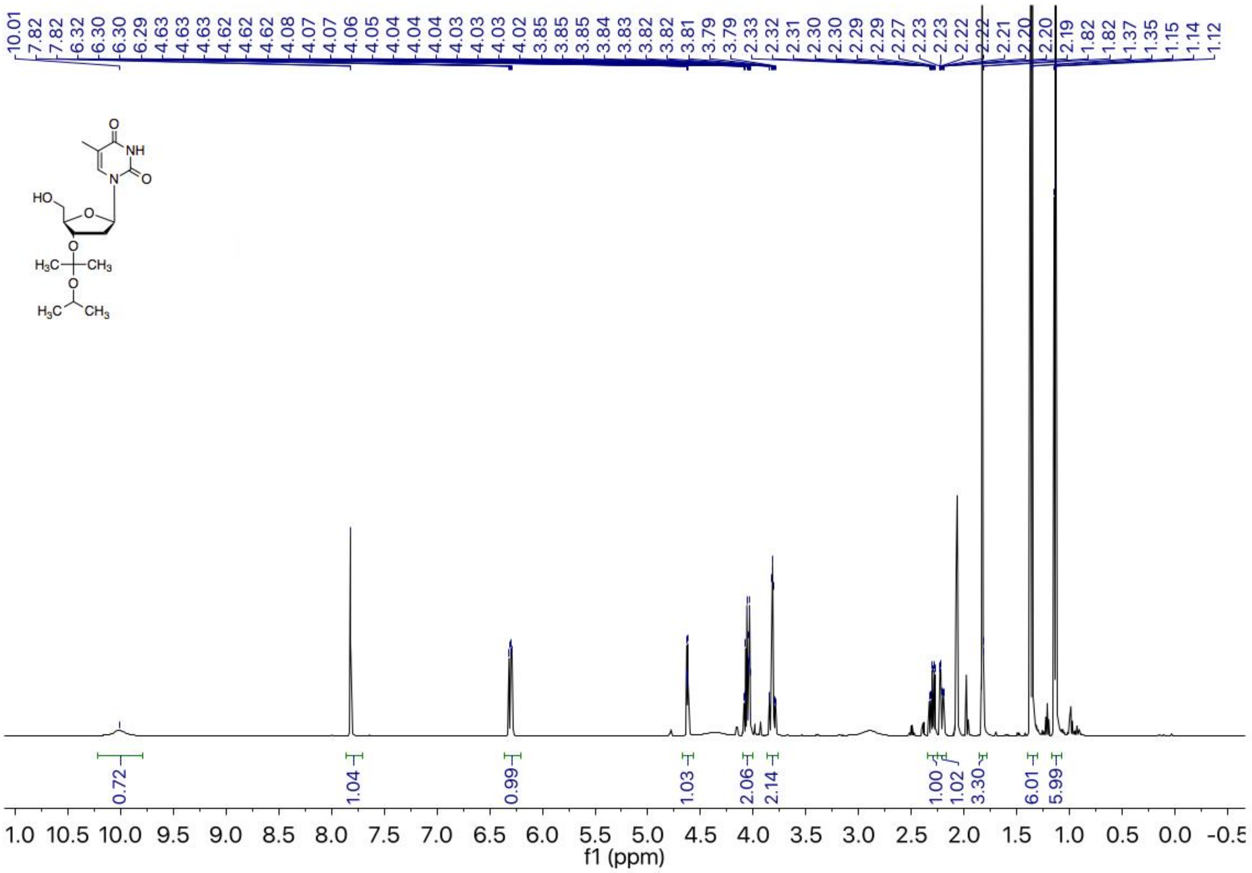

^{13}C-NMR (125 MHz, Acetone-d$_6$)

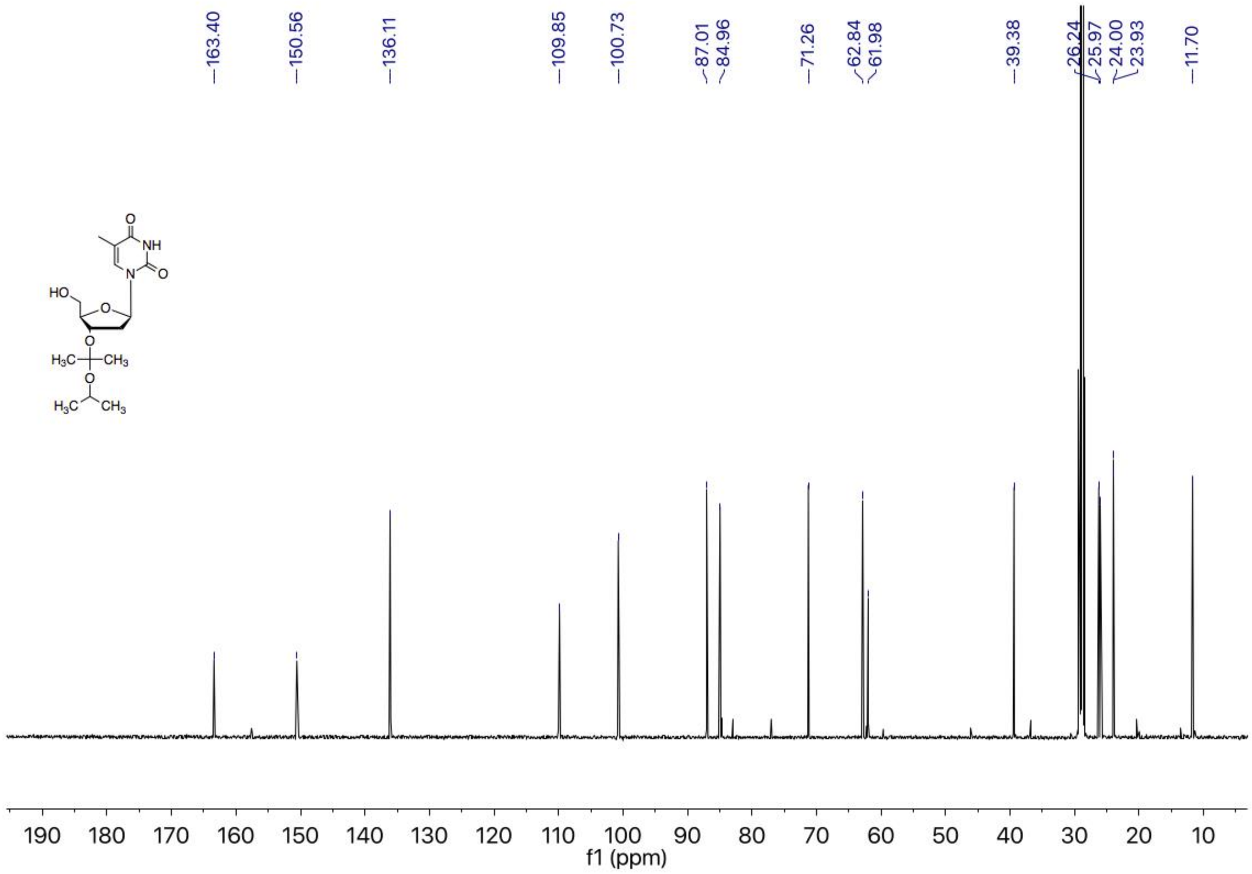

3'-O-(2,2,2-Trifluoroethoxy)propan-2-yl-2'-deoxythymidine (**7e**).

^{1}H-NMR (500 MHz, Acetone-d$_6$)

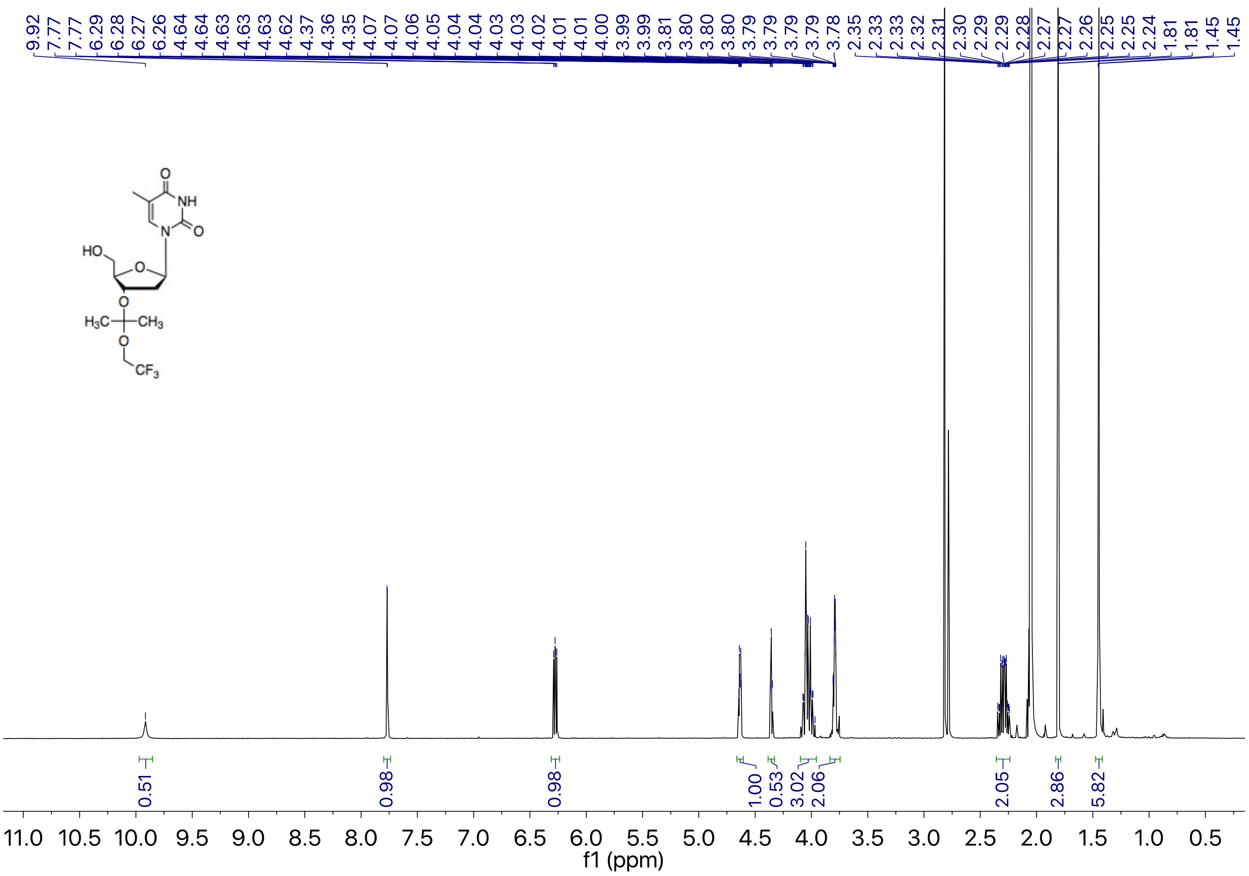

^{13}C-NMR (125 MHz, Acetone-d_6)

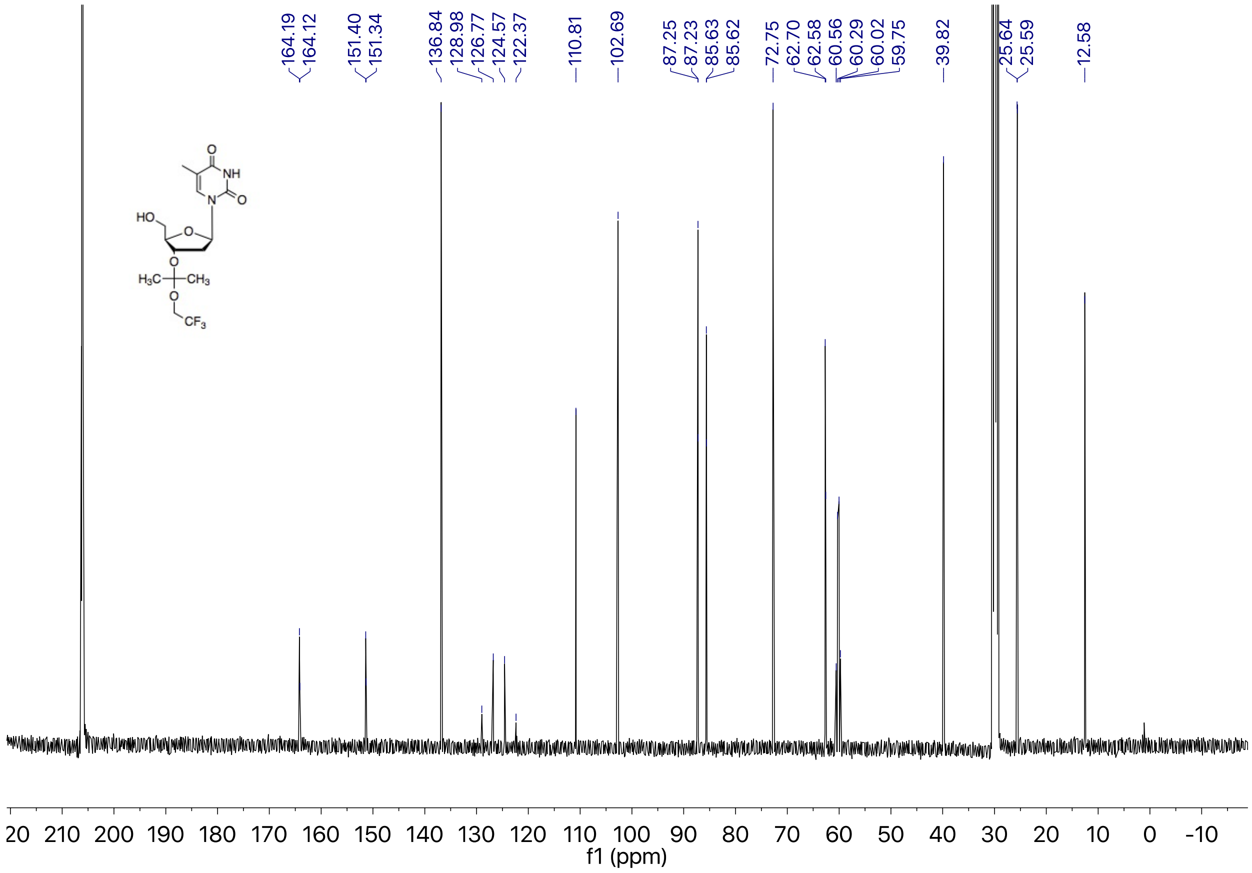

3’-O-Propen-2-yl-5’-O-tert-butyldimethylsilyl-2’-deoxythymidine (**8a**).

^{1}H-NMR (600 MHz, DMSO-d_6)

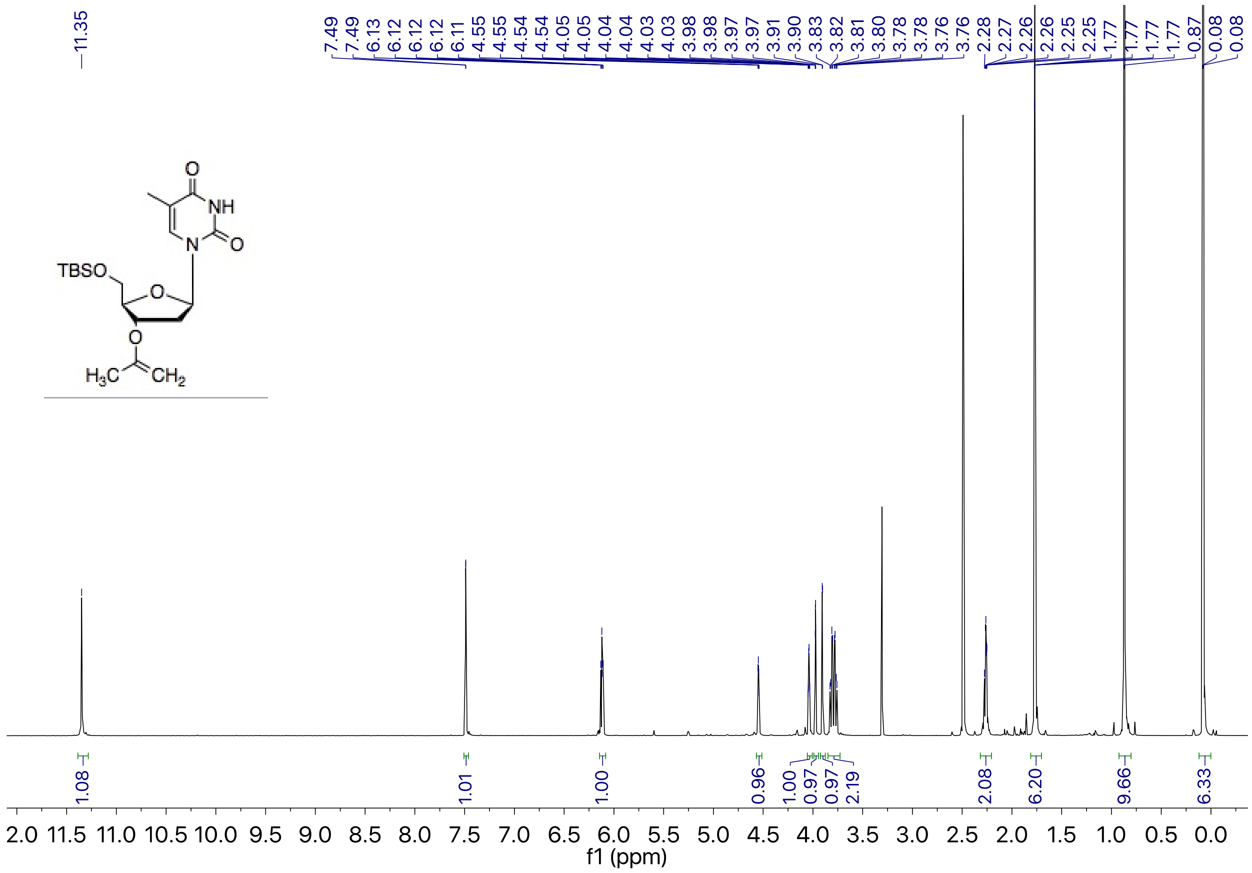

^{13}C-NMR (150 MHz, DMSO-d_6)

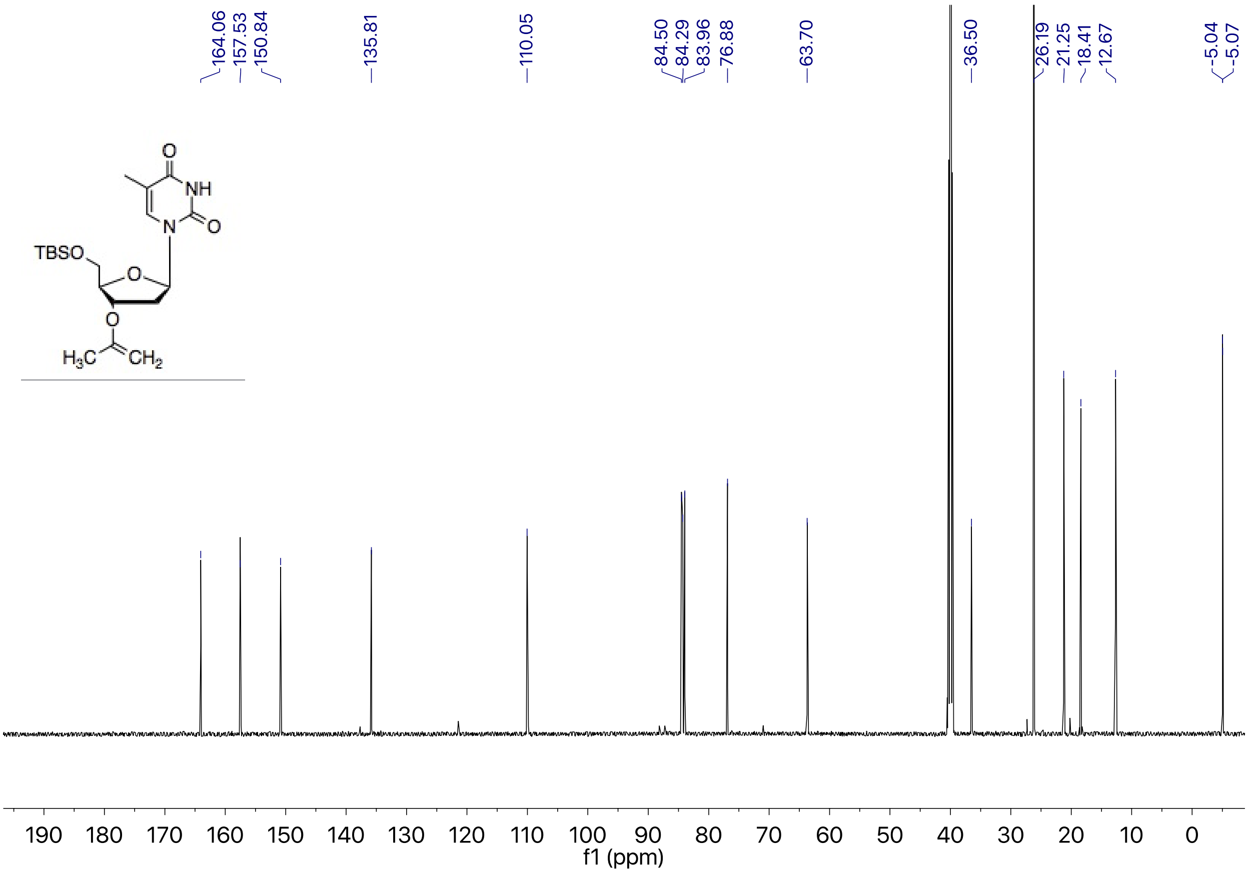

3'-O-Propen-2-yl-2'-deoxythymidine (**8b**).

^{1}H-NMR (600 MHz, DMSO-d$_6$)

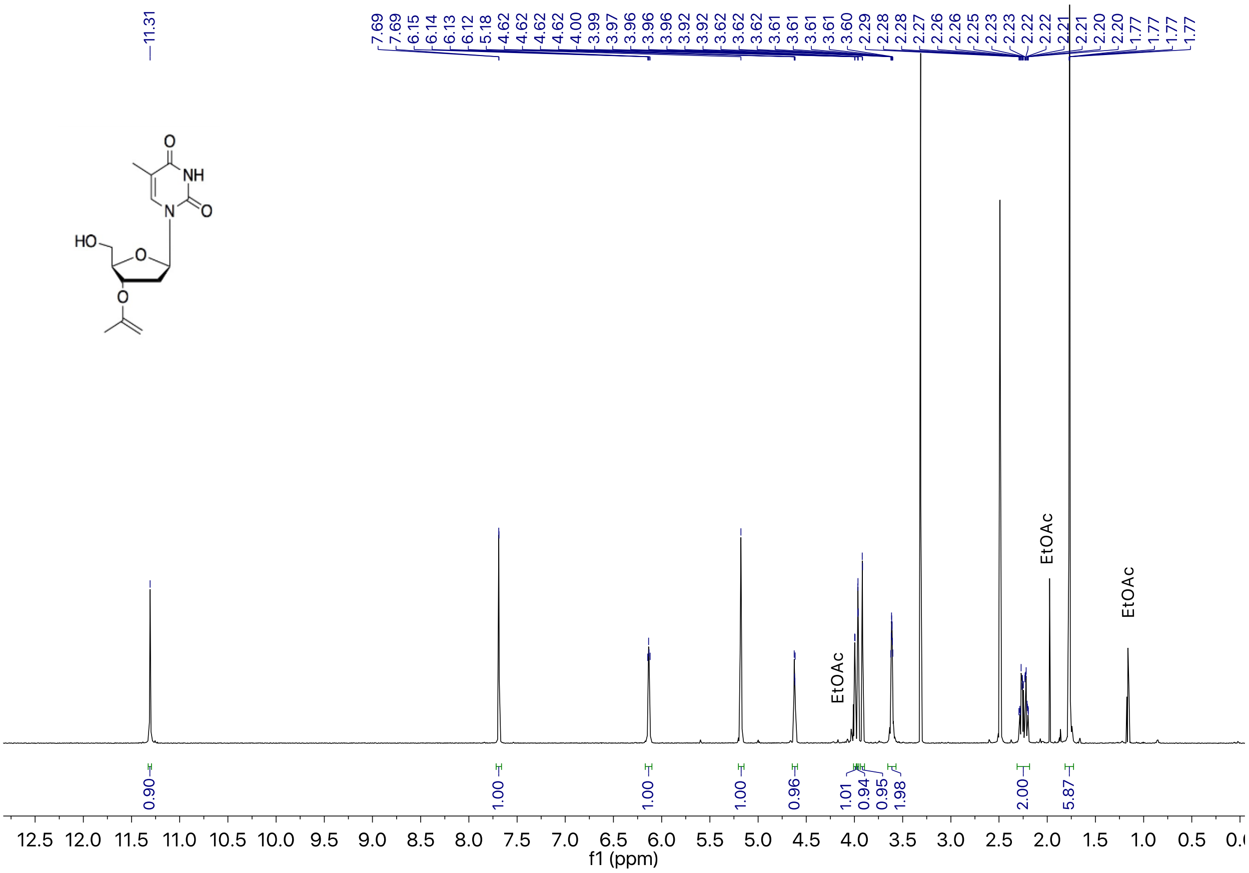

^{13}C-NMR (150 MHz, DMSO-d_6)

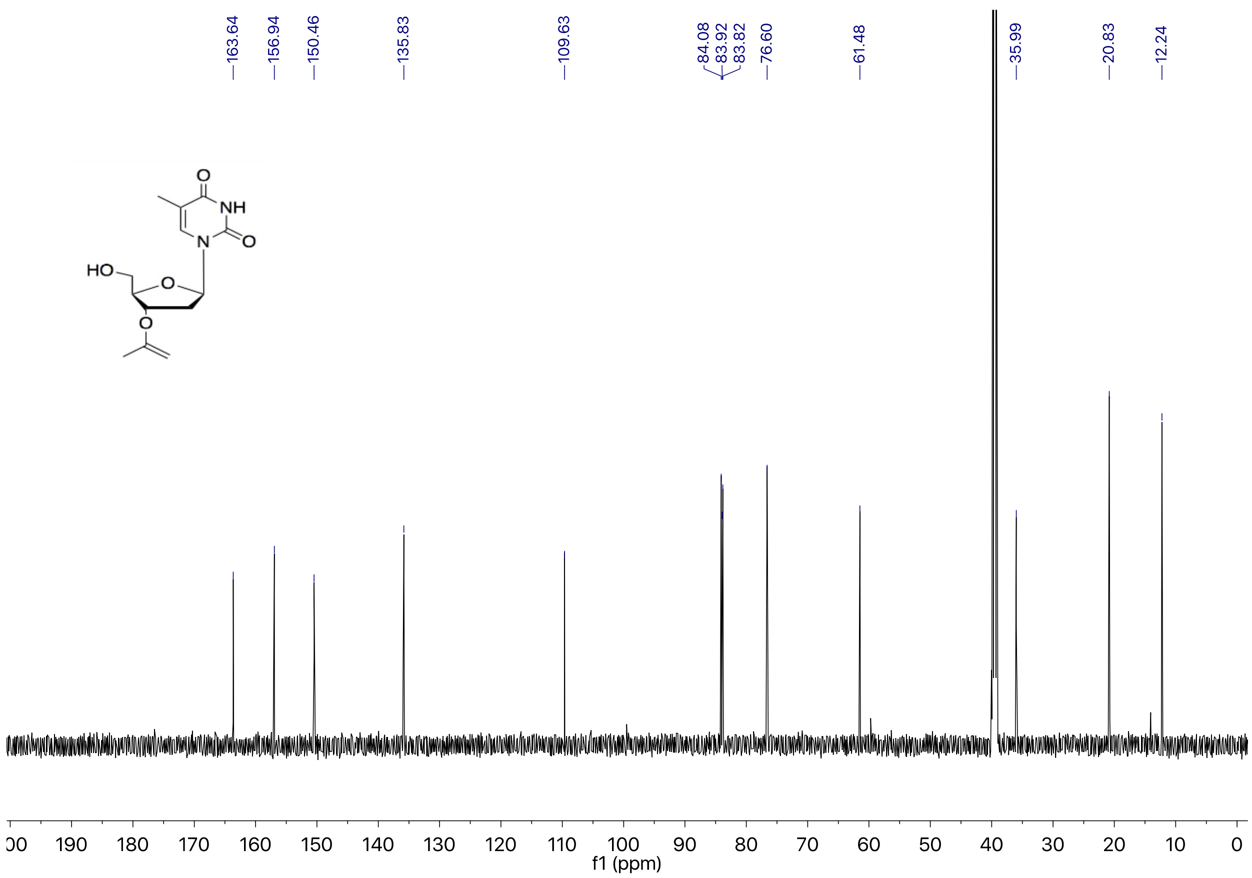

Supplement: File 2 — Copies of 1H NMR and 13C NMR spectra. [file Beilstein_J_Org_Chem-15-746-s002.pdf]
